# Early Eocene deep-sea benthic foraminiferal faunas: Recovery from the Paleocene Eocene Thermal Maximum extinction in a greenhouse world

**DOI:** 10.1371/journal.pone.0193167

**Published:** 2018-02-23

**Authors:** Gabriela J. Arreguín-Rodríguez, Ellen Thomas, Simon D’haenens, Robert P. Speijer, Laia Alegret

**Affiliations:** 1 Departamento de Ciencias de la Tierra, Universidad de Zaragoza, Zaragoza, Spain; 2 Department of Geology and Geophysics, Yale University, New Haven, Connecticut, United States of America; 3 Department of Earth and Environmental Sciences, Wesleyan University, Middletown, Connecticut, United States of America; 4 Department of Earth and Environmental Sciences, KU Leuven, Leuven, Belgium; 5 Instituto Universitario de Ciencias Ambientales, Universidad de Zaragoza, Zaragoza, Spain; Universita degli Studi di Urbino Carlo Bo, ITALY

## Abstract

The early Eocene greenhouse world was marked by multiple transient hyperthermal events. The most extreme was the Paleocene-Eocene Thermal Maximum (PETM, ~56 Ma), linked to the extinction of the globally recognised deep-sea benthic foraminiferal Velasco fauna, which led to the development of early Eocene assemblages. This turnover has been studied at high resolution, but faunal development into the later early Eocene is poorly documented. There is no widely accepted early Eocene equivalent of the Late Cretaceous-Paleocene Velasco fauna, mainly due to the use of different taxonomic concepts. We compiled Ypresian benthic foraminiferal data from 17 middle bathyal-lower abyssal ocean drilling sites in the Pacific, Atlantic and Indian Oceans, in order to characterise early Eocene deep-sea faunas by comparing assemblages across space, paleodepth and time. *Nuttallides truempyi*, *Oridorsalis umbonatus*, *Bulimina trinitatensis*, the *Bulimina simplex* group, the *Anomalinoides spissiformis* group, pleurostomellids, uniserial lagenids, stilostomellids and lenticulinids were ubiquitous during the early Eocene (lower-middle Ypresian). *Aragonia aragonensis*, the *Globocassidulina subglobosa* group, the *Cibicidoides eocaenus* group and polymorphinids became ubiquitous during the middle Ypresian. The most abundant early Ypresian taxa were tolerant to stressed or disturbed environments, either by opportunistic behavior (*Quadrimorphina profunda*, *Tappanina selmensis*, *Siphogenerinoides brevispinosa*) and/or the ability to calcify in carbonate-corrosive waters (*N*. *truempyi*). *Nuttallides truempyi*, *T*. *selmensis* and other buliminids (*Bolivinoides* cf. *decoratus* group, *Bulimina virginiana*) were markedly abundant during the middle Ypresian. Contrary to the long-lived, highly diverse and equitable Velasco fauna, common and abundant taxa reflect highly perturbed assemblages through the earliest Ypresian, with lower diversity and equitability following the PETM extinction. In contrast, the middle Ypresian assemblages may indicate a recovering fauna, though to some extent persistently disturbed by the lower-amplitude Eocene hyperthermals (e.g., Eocene Thermal Maximum 2 and 3). We propose the name ‘Walvis Ridge fauna’ for future reference to these Ypresian deep-sea benthic foraminiferal assemblages.

## 1. Introduction

Benthic foraminifera may constitute more than 50% of the total eukaryotic biomass at oceanic depths >1000 m [1–3], and are the biota most commonly represented in the microfossil record of the deep-sea floor, the largest habitat on Earth (e.g., [4]). Therefore, faunal and trace element/isotopic studies of deep-sea benthic foraminifera have been used extensively to infer past environmental conditions. These studies require detailed knowledge of benthic foraminiferal ecology and correct identification of morphological species to be chemically analysed, because many species show significant offsets in stable isotope and trace elemental signatures (e.g., [5, 6]). However, the taxonomy of benthic foraminifera is problematic, since splitting of species is common, providing different names for morphologically indistinguishable species, especially for different geographic regions and for different age intervals. Compilations of Early Cretaceous-middle Miocene [7], Late Cretaceous-Cenozoic [7, 8], Cenozoic [9], Late Cretaceous-Paleocene (K-Pg; [10]), and Paleocene-Eocene [11] faunas have been published, and benthic foraminiferal deep-sea biozones have been proposed for the Cenozoic [12–16]. In order to standardise descriptions of benthic foraminifera, Holbourn et al. [17] created a database of 300 deep-water species, including mostly taxa with stratigraphic and paleoecological significance, or those used in geochemical analyses. However, no detailed taxonomical global analysis similar to that for the Cretaceous-Paleocene Velasco fauna, as first described by Cushman [18, 19] and White [20–22], updated by Schnitker [23], Tjalsma and Lohmann [11], and Alegret and Thomas [10], has been performed for the lower Eocene. Müller-Merz and Oberhänsli [24] studied Eocene deep-sea benthic foraminifera, but concentrated on the South Atlantic region only.

The Paleocene-Eocene boundary (56 Ma; [25]) was a critical threshold for deep-sea benthic foraminifera, since this group suffered their largest extinction of the Late Cretaceous-Cenozoic [11, 13, 15, 26–30]. The extinction resulted in reorganisation of the assemblages, including the last appearance of about a quarter to half of the species, the first appearance of some species (e.g., *Anomalinoides capitatus* and *Hanzawaia ammophila*; [12]), and migration of species from shallower waters into the deep sea (e.g., [11, 28]). The faunal turnover across the Paleocene-Eocene boundary has been described in detail for many locations (e.g., [14, 29–43]). This turnover was synchronous with the start of the Paleocene-Eocene Thermal Maximum (PETM; e.g., [34, 44–46]), the most extreme hyperthermal event punctuating the long-term global warming trend of the early Paleogene (e.g., [16, 47–52]). Lower amplitude early Eocene hyperthermals have been described and evaluated from a sedimentological, isotopic and orbital-forcing perspective (e.g., [51, 53–59]), but benthic foraminiferal analyses are scarce [43, 60–63].

Despite the detailed faunal analyses across the Paleocene-Eocene transition, most studies do not follow the assemblages later into the early Eocene, and there is no consensus on the taxonomy of the most common early Eocene taxa. It has been suggested that early Eocene assemblages were less globally homogeneous than Paleocene assemblages [31], but this has not been well documented. We present a taxonomic compilation and partial revision of early Eocene deep-sea benthic foraminifera. This study covers almost the whole Ypresian (calcareous nannofossil and planktic foraminiferal biozones NP9b-NP12, CP8b-CP10, P5-P7, E1-E5 [64–68]), identifies the most common taxa and groups of taxa, and determines their paleogeographical and bathymetric distribution. The results of this compilation will improve current knowledge of early Eocene benthic foraminifera, as this is the first attempt to reconcile taxonomic assignments used by diferent authors and for different time intervals (e.g., Late Cretaceous, Early Cenozoic) globally. In addition, this study aims at evaluating variability in diversity, evolution of early Eocene faunas including extinctions and originations, and ecological preferences of Eocene species.

## 2. Materials and methods

### 2.1 Data acquirement

**Fig 1:**
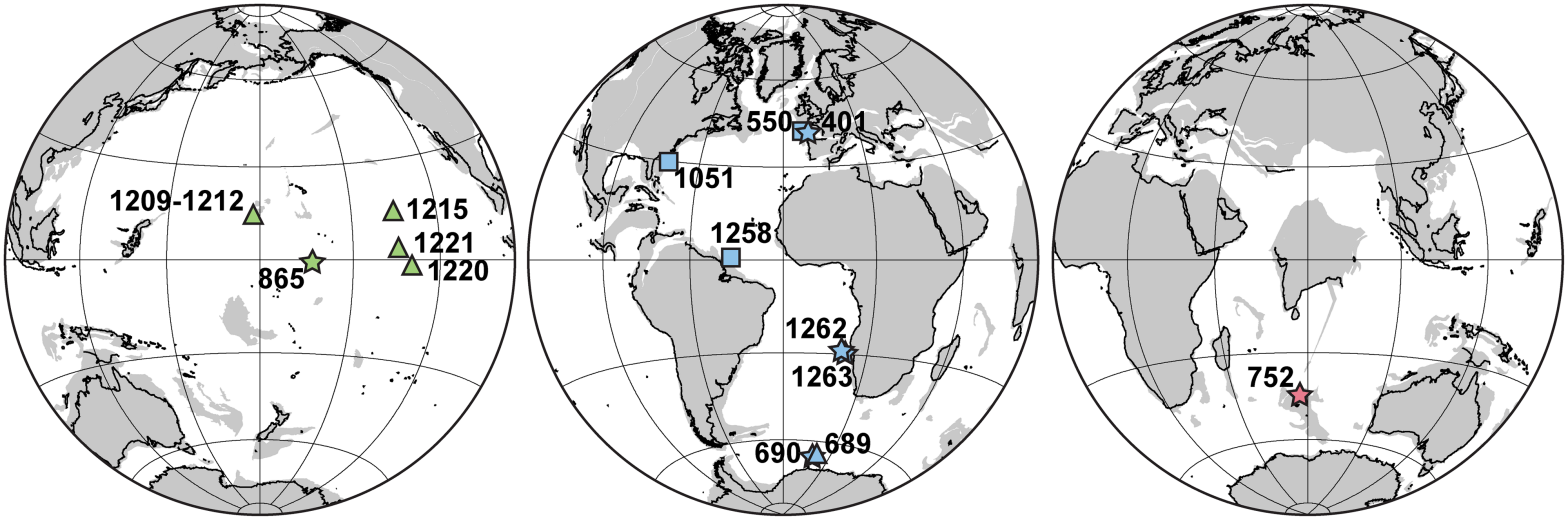
Location of ODP and DSDP sites reviewed in this study. Sites where benthic foraminifera are recorded across both Ypresian intervals (lower and middle) are indicated with stars, those encompassing only the lower Ypresian are marked with triangles, and those covering exclusively the middle Ypresian are shown with squares. Color reference: green-Pacific sites, blue-Atlantic sites, red-Indian site. Paleogeographic reconstruction of ~53 Ma from Hay et al., [69].

We compared benthic foraminiferal assemblages at seventeen sites drilled by the Ocean Drilling Program (ODP) and the Deep Sea Drilling Project (DSDP). The sites have a broad global distribution, covering the Atlantic, Pacific and Indian Oceans (Fig 1; Table 1), allowing the assessment of geographic and bathymetric variations. We confined this study to open ocean drilling sites.

**Table 1:** ODP and DSDP Sites/Holes compared in this study.

Ocean	Hole	Location	Latitude, longitude	Paleodepth	Sediment composition	Studied interval	Hyper-thermals^a^	References
Central Pacific	ODP 865B	Allison Guyot	18°26’N, 179°33’W	~1300–1500 m (lower bathyal)^1^	Foraminiferal-nannofossil ooze	Complete Ypresian (47.81–55.51 Ma)^2^	PETM, ETM3^2^	Sager et al., [70] ^1^Bralower et al., [71], ^2^Arreguín-Rodríguez et al., [43]
	ODP 1209B	Shatsky Rise	32°30’N, 158°30’E	1900 m (lower bathyal)	Clayey nannofossil ooze^3^	Biozone P5 and top of CP8^4^^,^^b^	PETM	Takeda and Kaiho, [36]; ^3^Bralower et al., [72, ^4^,^73^]
	ODP 1210B	Shatsky Rise	32°13’N, 158°15’E	2100 m (upper abyssal)	Nannofossil ooze^5^	Biozone P5 and top of CP8^4^	PETM	Takeda and Kaiho, [36]; ^5^Bralower et al., [74]
	ODP 1211C	Shatsky Rise	32°26’N, 158°42’E	2400 m (upper abyssal)	Nannofossil ooze^6^	Biozone P5 and top of CP8^4^	PETM	Takeda and Kaiho, [36]; ^6^Bralower et al., [75]
	ODP 1212B	Shatsky Rise	32°00’N, 158°51’E	2200 m (upper abyssal)	Nannofossil ooze with clay^7^	Biozone P5 and top of CP8^4^	PETM	Takeda and Kaiho, [36]; ^7^Bralower et al., [76]
	ODP 1215A	Molokai Fracture Zone	26°01.77’N, 147°55.99’W	~3000 m (upper -lower abyssal)	Nannofossil clay and nannofossil ooze^8^	Basal Eocene^8^ (biozones NP9, P5)	PETM^8^	Lyle et al., [77]; ^8^Nomura and Takata, [37]
	ODP 1220B	Clipperton and Clarion Fracture Zones	10°10.600’N, 142°45.503’W	~3000 m (upper—lower abyssal)	Clay and calcareous chalk^8^	Basal Eocene^8^ (biozones CP8b, P5)	PETM^8^	Lyle et al., [78]; ^8^Nomura and Takata, [37]
	ODP 1221C	Clipperton and Clarion Fracture Zones	12°01.999’N, 143°41.572’W	~3000 m (upper—lower abyssal)	Clay and calcareous chalk^8^	Basal Eocene^8^ (biozones CP8b, P5)	PETM^8^	Lyle et al., [79]; ^8^Nomura and Takata, [37]
Northeast Atlantic	DSDP 401	Meriadzek Terrace	47°25.65’N, 08°48.62’W	~1800–2000 m (lower bathyal—upper abyssal)	Calcareous nannofossil chalk	Basal Eocene^12^, up to the base of biozone NP12^9^	ETM2 (named δ)^9^	Montadert et al., [80]; ^9^D’haenens et al., [60]
	DSDP 550	Goban Spur	48°30.91’N, 13°26.37’W	~3900 m (lower abyssal)	Marly nannofossil chalk	Biozone NP11^10^	ETM2, H2, I1^10^^,^ ^11^	De Graciansky et al., [81]; ^10^D’haenens et al., [82]; ^11^Arreguín-Rodríguez and Alegret, [63]
Northwest Atlantic	ODP 1051A	Blake Nose	30°03.1740’N, 76°21.4580’W	1000–2000 m (lower bathyal)	Siliceous nannofossil chalk with clay	Biozone NP11^11^	ETM2, H2^11^^,^ ^12^	Norris et al., [83]; ^11^Nicolo et al., [54]; ^12^This study
Central Atlantic	ODP 1258B	Demerara Rise	9°26.0003’N, 54°43.9825’W	Lower bathyal—abyssal^12^	Nannofossil chalk with foraminifers	Magneto-chron C24r^13^	ETM2^12^^,^ ^13^	Erbacher et al., [84]; ^12^This study; ^13^Westerhold and Röhl, [85]
South Atlantic	ODP 1262	Walvis Ridge	27°11.15’S, 1°34.2’E	3500 m (lower abyssal)	Nannofossil ooze	Basal Eocene, up to ~52 Ma (biozones P7 and CP10)^14^	PETM, ETM2, ETM3^14^^,^ ^15^^,^ ^16^	Zachos et al., [86]; ^14^Röhl et al., [87]; ^15^Foster et al., [88]; ^16^Jennions et al., [62]
	ODP 1263	Walvis Ridge	28°31.98’S, 2°46.77’E	1500 m (lower bathyal)	Nannofossil ooze, chalky nannofossil ooze	Basal Eocene, up to ~52 Ma (P7, CP10)^14^	PETM, ETM2, ETM3^14^^,^ ^15^^,^ ^16^	Zachos et al., [89]; ^14^Röhl et al., [87]; ^15^Foster et al., [88]; ^16^Jennions et al., [62]
Southern Ocean	ODP 689B	Maud Rise	64°31.009’S, 03°05.996’E	1100 m (lower bathyal)^17^	Nannofossil foraminifer ooze	Basal Eocene^18^	PETM^18^	Barker et al., [90]; ^17^Thomas, [14]; ^18^Thomas and Shackleton, [34]
	ODP 690B	Maud Rise	65°9.629’S, 1°12.296’E	1900 m (lower bathyal)^17^	Foraminifer-bearing nannofossil ooze	Biozone CP8^17^, Magneto-chron C24r^18^	PETM^18^	Barker et al., [91]; ^17^Thomas, [14]; ^18^Thomas and Shackleton, [34]
Indian	ODP 752A	Broken Ridge	30°53.475’S, 93°34.652’E	~1000 m (middle -lower bathyal)	Nannofossil calcareous chalk	Biozones CP8, CP9a and CP9b^19^	PETM (identified as benthic extinction event)^19^	Peirce et al., [92]; ^19^Nomura, [93]

^a^ Hyperthermals recognised in the studied interval.

^b^ Biozones (P biozones—planktic foraminiferal zones; CP and NP biozones—calcareous nannoplankton biozones) shown as in the cited reference, independently of the biozone definition followed in each case.

^1–19^ Superscript numbers indicate the reference for specific data.

Most data have been previously published (Table 1), except those from ODP Sites 1258 and 1051 (S1 Table). PETM data from ODP Sites 1262 and 1263 have been reported partially in Foster et al. [88], data across ETM2 from those sites have been published by Jennions et al. [62], and across ETM3 preliminarily in Röhl et al. [87]. Complete data from ODP Sites 1262 and 1263 are included (S2 Table). D’haenens et al. [60] published benthic foraminiferal data across ETM2 from DSDP Site 401, but a taxonomically updated version of those data, including 18 additional samples from the most basal Eocene, is included here (S3 Table). All these samples were provided by the International Ocean Discovery Program (IODP) for the specific purpose of studying benthic foraminifera. No further permits were required for the described study. Sample material of unpublished faunal data from Sites 1051 and 1258 are stored at the Natural Science Museum of the University of Zaragoza (Spain, repository numbers MPZ-2018/2 to MPZ-2018/16), from Site 401 at the Division of Geology, Department of Earth and Environmental Sciences, KU Leuven (Belgium, repository numbers FA-2018-1 to FA-2018-64), and from Sites 1262 and 1263 at the Yale Peabody Museum of Natural History (USA, repository numbers 540338 to 540414, 540417 to 540493, and 540501 to 540609). Complete repository information (e.g., geographic location) and detailed list of the repository numbers are included in S1–S3 Tables. All these samples are accessible in the corresponding repositories.

**Fig 2:**
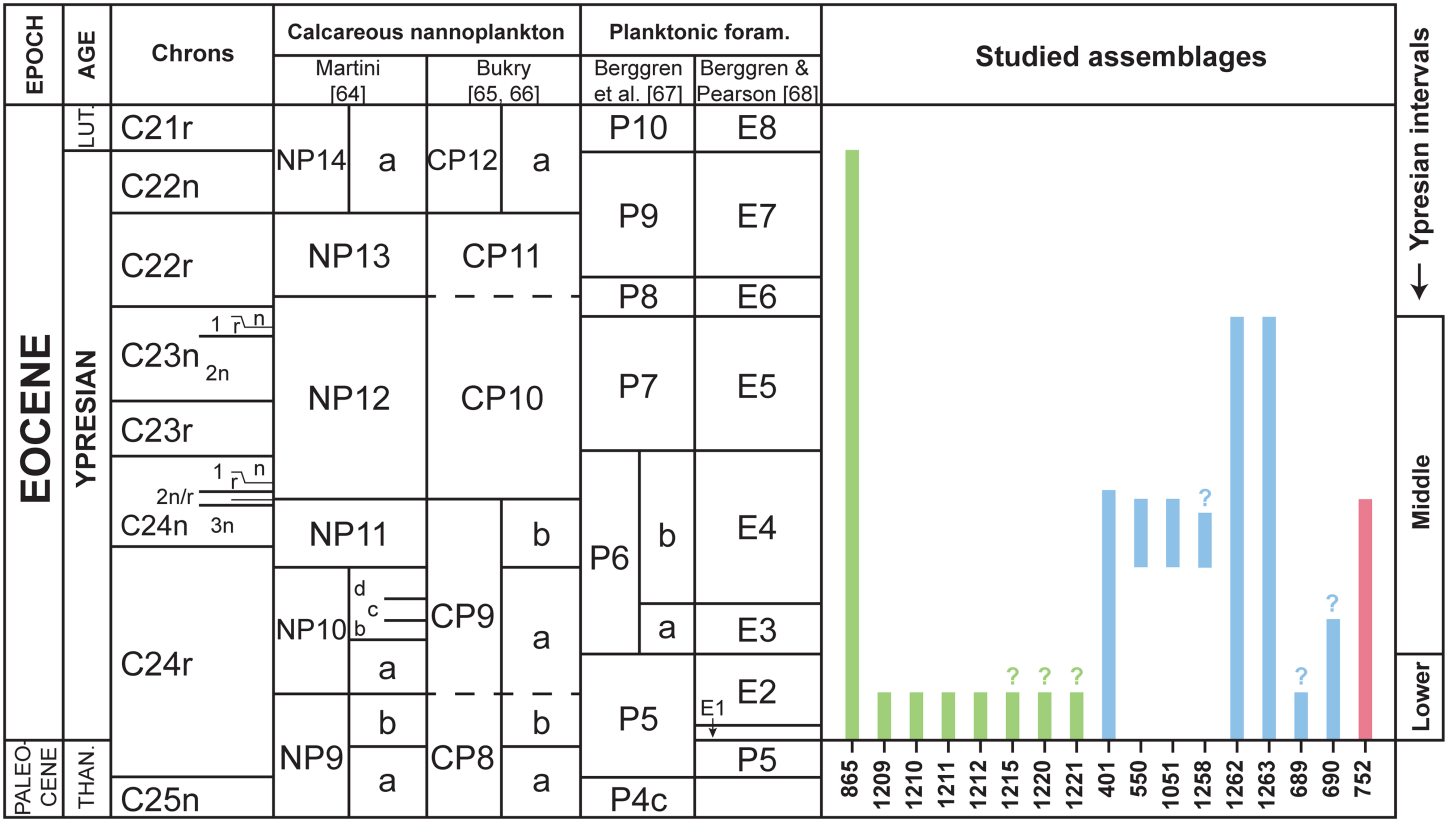
Stratigraphic interval represented at each ODP/DSDP site included in this study. Lower Eocene zonation scheme from Berggren and Pearson [68], references of each drilling site included in Table 1. Color reference: green-Pacific sites, blue-Atlantic sites, red-Indian site.

In order to accurately compare early Eocene benthic foraminiferal assemblages, the records were split (whenever possible) into two time intervals, after careful examination of the biostratigraphy at each site. The first interval corresponds to the lower Ypresian (~56–55.2 Ma [25]), from immediately above the Paleocene-Eocene boundary up to the upper limit of planktonic foraminiferal zone P5 as defined by Berggren et al. [67]. Biozone P5 was frequently used in older literature, and covers the uppermost Paleocene and basal Eocene. This zone corresponds to biozones P5, E1 and E2 in the revised zonation of Berggren and Pearson [68] (Fig 2). In this revised zonation, the E-zones are Eocene in age [68]. The second interval (~55.2–50.7 Ma [25]), here called middle Ypresian, comprises the stratigraphic interval from the base of planktonic foraminiferal zone P6 up to the top of P7 of Berggren et al. [67]. According to Berggren and Pearson [68], Biozone P6 is equivalent to biozones E3 and E4, whereas Biozone P7 corresponds to Biozone E5 (Fig 2).

The relative abundance of species (percentage) in the literature is typically presented by sample, but in order to estimate the abundance of each species across the two study intervals (lower and middle Ypresian) by site, the percentage of each species relative to the total number of specimens counted per site was calculated. When the number of specimens counted was not available from the published data (ODP Sites 1209, 1210, 1211 and 1212), we calculated the average percentage of every species by site, and then rescaled these averages to obtain an actual representative percentage (i.e., the fraction that such species represents within the total assemblage). We thus focused on the total relative abundance of each species across each study interval by site (S4–S6 Tables). These numbers depend upon the number of samples studied per time slice, but we consider them as representative of observations at each site.

In order to compare benthic foraminiferal assemblages from different sites as documented by different authors, it was necessary to standardise the data, because different authors use different taxonomic concepts, a major problem in compiling taxonomic data (e.g., [17]). After establishing informal morphological supra-generic groups to simplify the taxonomy (Table 2; S4 and S5 Tables), we performed a careful bibliographic revision and examined type specimens at the American Museum of Natural History, New York (AMNH) and at the Smithsonian National Museum of Natural History, Washington DC (NMNH). We then grouped species with similar morphologies and/or specimens that may belong to the same morphological species, but have been identified under different names (Table 3).

**Table 2:** Taxa included into supra-generic groups of benthic foraminifera, based on their main morphological characteristics.

Lenticulinids	Pleurostomellids	Polymorphinids	Stilostomellids	Unilocular taxa	Uniserial lagenids
*Astacolus*	*Nodosarella*	*Ellipsodimorphina*	*Siphonodosaria*	*Buchnerina*	*Chrysalogonium*
*Lenticulina*	*Obesopleurostomella*	*Ellipsoglandulina*	*Stilostomella*	*Fissurina*	*Dentalina*
*Saracenaria*	*Pleurostomella*	*Ellipsoidella*	*Strictocostella*	*Lagena*	*Glandulonodosaria*
	*Paleopleurostomella*	*Ellipsoidina*		*Oolina*	*Laevidentalina*
		*Ellipsopolymorphina*		*Palliolatella*	*Laterohiatus*
		*Globulina*			Nodosariids
		*Paleopolymorphina*			*Pseudonodosaria*
		Polymorphinids			
		*Pseudopolymorphina*			
		*Pyrulina*			
		*Pyrulinoides*			

**Table 3:** Informal taxonomic groups of similar taxa observed within the lower Eocene (lower and middle Ypresian) at ODP and DSDP sites analysed in this study.

***Bolivinoides* cf. *decoratus* group**	***Bolivina huneri* group**	***Siphogenerinoides brevispinosa* group**	***Fursenkoina fusiformis* group**
*B*. *decoratus*	*B*. *crenulata/huneri*	*S*. *brevispinosa*	*F*. *fusiformis*
*B*. cf. *decoratus*	*B*. *huneri*	*S*. cf. *brevispinosa*	*Stainforthia fusiformis*
*B*. *decorata*	*Bolivinoides huneri*		
***Buliminella beaumonti* group**	***Bulimina elongata* group**	***Bulimina simplex* group**	**Triangular buliminids group**
*B*. *beaumonti*	*B*. *elongata*	*B*. *simplex*	*Bulimina prolixa*
*B*. cf. *beaumonti*	*B*. *thanetensis*	*B*. *bradburyi*	*Pyramidina rudita*
	*B*. cf. *thanetensis*	*B*. *tuxpamensis*	
	*B*. cf. *simplex*		
**Costate buliminids group**	**Fusiform buliminids group**	***Globocassidulina subglobosa* group**	***Nonion havanense* group**
*Bulimina jarvisi*	*P*. *reussi*	*G*. *subglobosa*	*N*. *havanense*
*B*. *semicostata*	*Bulimina* sp. 1	*G*. *globosa*	*N*. cf. h*avanense*
*B*. cf. *semicostata*	*B*. *kugleri (ovula)*		
	*B*. *kugleri*		
	*B*. *trihedra*		
	*Fursenkoina* sp. 2		
	*Fursenkoina* sp. 3		
***Pullenia jarvisi* group**	***Abyssamina incisa*-*poagi* group**	***Eilohedra weddellensis* group**	***Anomalinoides spissiformis* group**
*P*. *jarvisi*	*A*. *incisa*	*E*. *weddellensis*	*A*. *spissiformis*
*P*. *subcarinata*	*A*. *poagi*	*Alabaminella weddellensis*	*A*. *praespissiformis*
*P*. *americana*			*A*. *ammonoides/spissiformis*
			*A*. *praeacuta*
			*A*. *praeacutus*
***Anomalinoides capitatus* group**	**Small *Cibicidoides / Anomalinoides* group**	***Cibicidoides* with an umbo group**	***Cibicidoides mundulus* group**
*A*. *capitatus*	*Cibicidoides micrus*	*C*. *alleni*	*C*. *mundulus*
*A*. *capitatus*/*danicus*	*C*. *subcarinatus*	*C*. *dayi*	*C*. *praemundulus*
*A*. *rubiginosus*	*C*. *ungerianus*	*Anomalinoides trinitatensis*	*C*. *pseudoperlucidus*
	*Anomalinoides* cf. *acutus*		*C*. *proprius*
	*A*. cf. acutus (small)		*C*. *howelli*
***Cibicidoides eocaenus* group**	**Flat *Gyroidinoides* group**	***Nuttallides umbonifera* group**	
*C*. *eocaenus*	*G*. *planulatus*	*N*. *umbonifera*	
*C*. *tuxpamensis*	*G*. *planulatus (complanata)*	*N*. *umbonifera*?	
*C*. *perlucidus*	*G*. *depressus*	*Nuttallides* sp. 2	
*C*. *eocaenus tuxpamensis*	*Valvalabamina depressa*	*Osangularia* sp. 1	
*C*. *eocaenus perlucidus*		*Osangularia* sp.	
		*Eponides elevatus*	

The names included here correspond to the names used by the original authors.

#### 2.1.1 Sample preparation and picked specimens

We report new taxonomic data across the lower Eocene from ODP Sites 1051, 1258, 1262, 1263 and DSDP Site 401 (S1–S3 Tables). The very low numbers of benthic foraminifera at Sites 1051 and 1258 did not allow us to perform a detailed quantitative analysis of the assemblages, but we selected eight (Hole 1051A) and nine samples (Hole 1258B) from these sites to represent faunal composition in the central and tropical western Atlantic, respectively.

Samples were cleaned by removing the edges of the core in contact with the liner to avoid contamination, soaked in water with detergent, washed over a 63 μm sieve and the residue was dried at ~50°C. Benthic foraminifera were picked from the >63 μm fraction. An average of 386 specimens per sample were picked for DSDP Site 401, whereas an average of 120 specimens per sample was picked from ODP Site 1051, and 70 specimens per sample from ODP Site 1258 (S1 Fig). The low abundance numbers of the two latter sites are mostly due to strong dilution of benthic foraminifera by other components in the sediment. They are well below the ~300 specimens that are usually picked for quantitative analyses (e.g., [30, 94]), and necessary to fully represent diversity. However, since in this case we are considering the relative abundance across the entire site, we considered that these numbers may reflect the overall diversity and abundance at Sites 1051 and 1258. For most samples at Sites 1262 and 1263, several hundreds of specimens were obtained, with the exception of an interval of strong dissolution at the base of the PETM that did not contain any benthic foraminifera, not even agglutinated forms.

#### 2.1.2 Paleobathymetry

**Fig 3:**
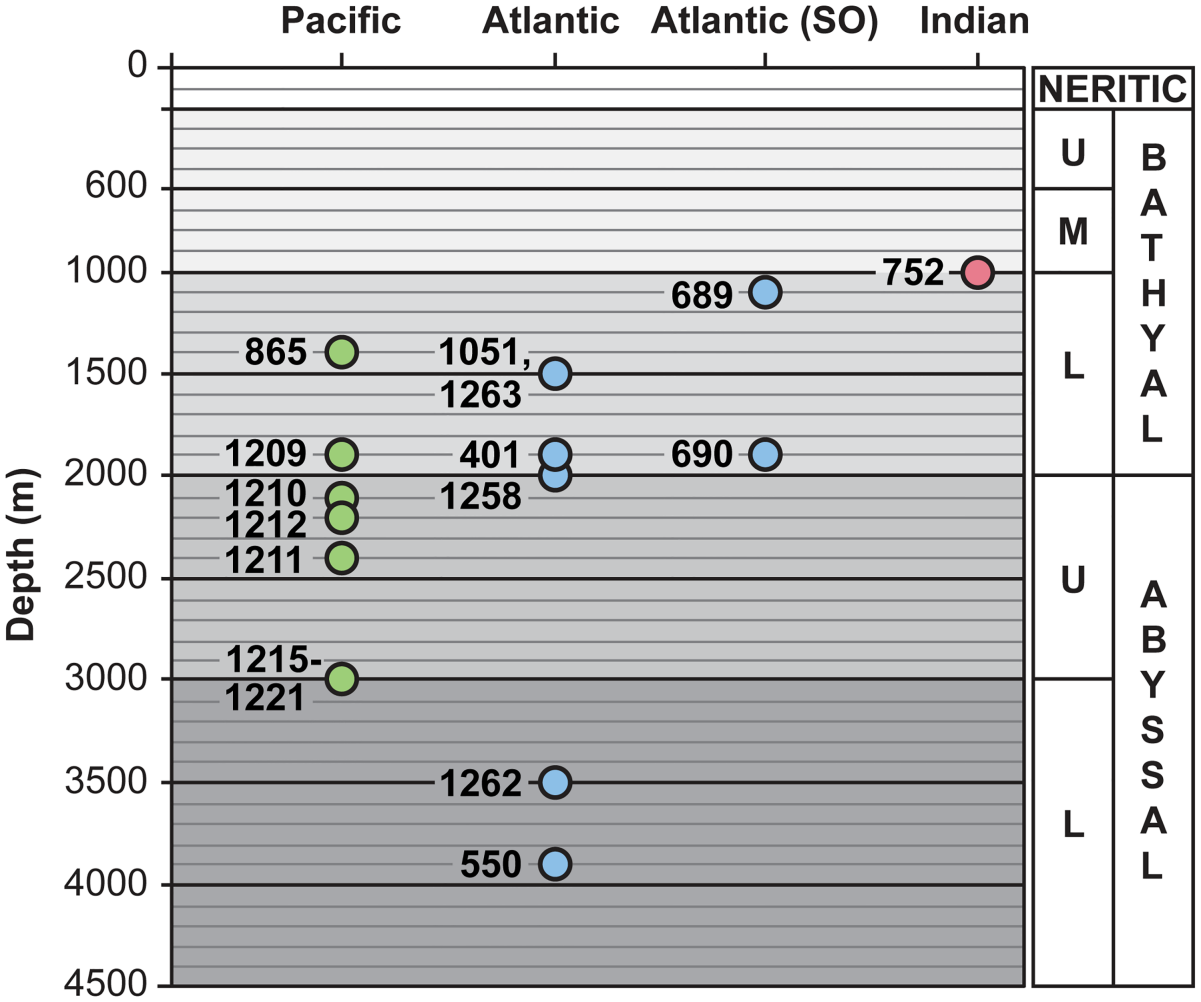
Paleobathymetry of ODP and DSDP sites. The circles represent the average of the depth range estimates, full data are shown in Table 1. Abbreviations: SO = Southern Ocean; U = upper; M = middle; L = lower.

We use bathymetric divisions as defined in Van Morkhoven et al. [9]: upper bathyal (200–600 m), middle bathyal (600–1000 m), lower bathyal (1000–2000 m), upper abyssal (2000–3000 m) and lower abyssal (>3000 m). The early Eocene paleodepth of the studied sites (with the exception of ODP Site 1258) has been previously documented (Table 1; Fig 3) [31, 95], mainly based on benthic foraminiferal assemblages. The original depth assignments of the assemblages are generally based on backtracking information (e.g., [10]) and/or comparison of assemblages as they occur along depth transects (e.g. [11]). We inferred the paleodepth at Site 1258 based on benthic foraminiferal evidence, as compared to asemblages at the other sites.

Benthic foraminiferal assemblages at ODP Site 1258 are dominated by calcareous taxa, suggesting deposition well above the calcite compensation depth. Most of the abundant and common species from this site have been described from lower bathyal to abyssal depths, and include *Quadrimorphina profunda*, *Clinapertina subplanispira*, *C*. *inflata*, *Anomalinoides spissiformis*, *Nonion havanense*, *Nuttallides umbonifera* and stilostomellid species [11, 96]. Other common species such as *Nuttallides truempyi*, *Aragonia aragonensis*, *Oridorsalis umbonatus*, pleurostomellids and *Bulimina trinitatensis* have been reported across wider depth ranges, varying from bathyal or middle bathyal to abyssal (e.g., [9, 11, 96]). Some common species such as *Globocassidulina subglobosa* and *Bulimina tuxpamensis* have a reported upper depth limit at upper bathyal depths [9]. However, some species such as *A*. *aragonensis*, *G*. *subglobosa* and occasionally even *N*. *truempyi* have been reported in outer neritic parts of the Tethyan shelves during the early Eocene (e.g., [97, 98]), and *Osangularia plummerae*, another common species, typically inhabits a wide range of depths, from lower sublitoral to upper bathyal depths [99]. Thus we infer a lower bathyal to abyssal paleodepth for Site 1258 as most probable during the Ypresian.

Almost all sites for which data are presented here range from lower bathyal to upper abyssal paleodepths, except for the shallower Indian ODP Site 752, and the deepest Atlantic ODP Site 1262 and DSDP Site 550 (Fig 3). The vertical range of paleodepths of Pacific sites is narrower than that of the Atlantic Ocean sites, with paleodepths for Pacific sites ranging from lower bathyal to upper abyssal (~1300–3000 m), whereas paleodepth of Atlantic sites ranges from lower bathyal to lower abyssal (~1100–3900 m).

#### 2.1.3 Microhabitats

The microhabitat distribution of benthic foraminifera was used to interpret the ecology of the assemblages following the TROX model (e.g., [100, 101]), with the infaunal-epifaunal (or endobenthic-epibenthic) ratio used as a proxy for some combination of oxygen levels and trophic conditions at the seafloor. According to this model, a high relative abundance of infaunal taxa is indicative of a high food supply and/or low oxygen availability, and a low percentage of infaunal taxa is indicative of oligotrophic and well-oxygenated conditions at the seafloor. This model in its simplest form, however, may not be valid under all circumstances. For instance, calcareous infaunal taxa may be advantaged relative to epifaunal taxa during carbonate dissolution at the seafloor [88], or bottom currents may lead to trophic focusing, so that relatively high abundances of suspension feeding, infaunally living taxa occurred even at low primary productivity in the overlying waters [43].

Benthic foraminiferal taxa were assigned to microhabitats based on their test morphology: epifaunal species are characterised by milioline coiling, planoconvex and biconvex trochospiral tests, biconvex planispiral, rounded and flat tests, tubular, and leaf-like forms, and infaunal taxa mainly have cylindrical, spherical, ovoid, unilocular globose, rounded planispiral and streptospiral shapes, and also include taxa with a neck (e.g., [102–104]). However, the relation between morphology of the test and microhabitat is not straightforward. There are many exceptions among living taxa (e.g., [105]), so that assignments may be accurate only about 75% of the time [106]. Despite these complications, this index provides a basis for evaluating the relationship between benthic foraminifera and environmental conditions at the seafloor, as controled by primary productivity at the sea-surface through bentho-pelagic coupling [107], but influenced by variability in remineralization of organic matter during transport to the seafloor, as well as potential lateral influx of refractory organic matter (e.g., [108, 109]).

### 2.2 Statistical analyses

Multivariate analyses, including cluster and non-metric multidimensional scaling (NMDS), have been performed to assess the early Eocene benthic foraminiferal assemblages, using PAST software v. 3.04 [110]. These techniques were applied in order to identify the main assemblages and the most typical benthic foraminifera across the Ypresian in distinct regions. Hierarchical cluster analysis is an explorative technique applied to identify groups of sites with similar benthic foraminiferal assemblages, using the unweighted pair group average algorithm (UPGMA) and the Pearson correlation as similarity coefficient. NMDS is an ordination method to visualise trends or groupings, and useful when the dataset shows null values, here represented by the absence of species. Considering that the values of the relative abundance (percentages) of the species may depend on the size and number of samples studied per site, we chose NMDS as an optimal technique, because it does not compare actual abundance percentages and transforms the distance measures into ranks, comparing these ranked distances with the rank of the Euclidean distances in the ordination plot [111]. The NMDS plots represent the data considering their rank order (based on abundances), but discarding absolute percentage values. The Euclidean distance was used as the similarity index because it is sensitive to high abundances.

Both analyses were carried out for the intervals corresponding to the lower and middle Ypresian. The lower Ypresian dataset includes 29 species groups from 14 sites (Table 4), and the middle Ypresian dataset consists of 42 species groups from 9 sites (Table 5). Tables 4 and 5 were constructed considering those taxa or groups of taxa that are common (≥2 to <5%), abundant (≥5 to <15%) or very abundant (≥15%) in at least one site, but they do not include species identified in one publication only or by one author only, in order to avoid author-biased results.

**Table 4:** Relative abundance of benthic foraminifera from ODP and DSDP sites across the lower Ypresian.

Species	ODP 865B	ODP 1209B	ODP 1210B	ODP 1211C	ODP 1212B	ODP 1215A	ODP 1220B	ODP 1221C	DSDP 401	ODP 1262	ODP 1263	ODP 689B	ODP 690B	ODP 752A
*Abyssamina quadrata* (Aqua)	0.39	0.00	0.00	0.00	0.00	1.88	2.21	4.01	2.22	7.02	6.42	0.34	3.51	0.00
*Anom*. *capitatus* group (Acap)	0.00	0.00	0.00	0.00	0.00	0.00	0.00	0.00	0.09	0.00	0.00	0.00	0.02	15.51
*Anom*. *spissiformis* group (Aspi)	0.00	0.00	0.00	0.78	0.00	7.53	5.19	7.93	2.59	4.06	1.10	0.69	1.51	0.69
*Aragonia aragonensis*(Aara)	1.86	0.00	0.00	0.00	0.00	0.00	0.00	0.05	0.64	1.69	6.65	2.36	1.15	0.00
*Boli*. cf. *decoratus* group (Bdec)	9.31	0.00	0.00	0.00	0.00	0.00	0.00	0.00	0.00	3.24	0.49	3.22	7.57	0.00
*Bulimina elongata* group (Belo)	0.20	1.29	1.84	1.06	2.48	0.00	0.00	0.00	6.25	0.03	0.43	3.31	1.99	0.00
*Bulimina simplex* group (Bsim)	6.53	8.64	5.84	3.98	6.69	0.20	2.06	2.05	5.42	0.90	10.10	14.39	8.25	5.10
*Bulimina trinitatensis* (Btri)	1.31	1.33	0.00	0.00	0.00	0.00	0.00	0.00	0.39	0.25	3.02	2.75	1.20	3.59
*Bulim*. *beaumonti* group (Bbea)	2.03	1.88	1.49	0.00	0.00	0.00	0.48	0.01	0.00	0.00	0.26	0.00	0.05	0.00
*Cib*. *mundulus* group (Cmun)	2.39	0.00	0.00	0.00	0.00	0.00	0.00	0.00	8.02	0.45	1.07	1.72	0.27	0.00
*Clinap*. *subplanispira* (Csubp)	0.00	0.00	0.00	0.00	0.00	0.00	0.00	0.00	0.00	8.80	2.57	0.34	0.59	0.00
Costate bulim. group (CoBul)	13.66	0.00	0.00	0.00	0.00	0.00	0.73	0.00	0.02	0.00	0.00	0.00	0.00	0.00
*Epistominella exigua* (Eexi)	0.00	0.00	0.00	0.00	0.00	0.00	0.00	0.00	11.46	0.14	0.40	0.00	0.00	0.00
Flat *Gyroidinoides* group (FlGyr)	0.22	0.00	0.00	0.00	0.00	0.02	0.00	0.27	2.13	0.96	0.33	0.21	0.65	0.00
Fusiform bulim. group (FuBul)	0.00	8.41	8.51	7.93	10.82	0.53	3.40	1.15	5.17	8.06	6.61	3.18	8.39	0.00
*Glob*. *subglobosa* group (Gsub)	0.27	2.62	3.34	1.67	3.11	1.18	9.26	6.30	5.48	1.97	2.23	0.17	0.07	0.00
Lenticulinids (Lent)	4.98	0.49	0.53	0.00	0.00	0.02	0.12	0.13	0.23	0.00	3.13	2.79	1.02	8.91
*Nonionella robusta* (Nrob)	0.02	0.00	0.00	0.00	0.00	0.00	0.00	0.00	0.00	2.14	1.12	0.56	0.57	0.00
*Nuttallides truempyi* (Ntru)	0.73	7.78	8.47	8.59	7.33	40.67	15.28	17.42	1.20	14.86	14.36	1.20	2.33	14.81
*Nutt*. *umbonifera* group (Numb)	0.44	0.00	3.48	5.99	2.09	0.02	3.50	3.50	11.13	0.00	0.00	3.99	1.77	0.00
*Oridorsalis umbonatus* (Oumb)	3.46	1.51	1.11	0.62	0.71	1.84	5.40	4.51	1.49	2.85	3.45	2.36	1.11	10.65
Pleurostomellids (Pleu)	6.82	7.03	3.85	3.77	3.79	0.61	3.16	1.20	3.73	5.19	4.94	3.44	3.05	0.69
*Pullenia jarvisi* group (Pjar)	0.12	0.00	0.00	0.00	0.00	13.52	3.33	2.78	1.24	0.00	0.01	0.21	0.13	1.62
*Quadrim*. *profunda* (Qpro)	0.00	18.57	16.58	7.76	19.63	23.05	32.09	30.83	1.10	8.46	1.73	0.90	0.62	0.00
*Sip*. *brevispinosa* group (Sbre)	1.11	0.78	5.19	10.17	5.78	0.00	0.00	0.00	1.06	4.20	3.71	9.45	23.22	0.00
Stilostomellids (Stil)	5.25	0.22	0.47	0.74	2.00	0.00	0.00	0.03	0.37	0.11	0.88	5.41	5.19	0.58
*Tappanina selmensis* (Tsel)	12.04	1.02	1.21	3.34	1.69	6.43	6.07	8.07	7.19	6.54	7.25	24.79	12.37	0.00
*Turrilina brevispira* (Tbre)	0.31	0.00	0.00	0.00	0.00	0.00	0.00	0.00	0.54	1.52	1.56	2.88	1.41	0.12
Uniserial lagenids (UnLag)	4.86	0.90	6.21	1.00	6.03	0.04	0.39	0.44	1.06	0.54	1.09	1.76	1.07	0.81

**Table 5:** Relative abundance of benthic foraminifera from ODP and DSDP sites across the middle Ypresian.

Species	ODP 865B	DSDP 401	DSDP 550	ODP 1051A	ODP 1258B	ODP 1262	ODP 1263	ODP 690B	ODP 752A
*Abys*. *incisa*-*poagi* group (Ainc)	0.10	0.17	2.27	1.04	0.16	5.87	5.12	0.47	0.00
*Alabamina dissonata* (Adis)	0.00	0.00	0.00	0.00	0.00	0.52	0.70	0.00	5.22
*Eilo*. *weddellensis* group (Ewed)	0.02	2.42	0.00	0.00	0.00	0.25	0.04	0.00	0.00
*Anom*. *capitatus* group (Acap)	0.00	0.09	0.09	0.00	0.00	0.00	0.00	0.00	10.95
*Anom*. *spissiformis* group (Aspi)	0.14	4.21	1.20	0.00	2.67	4.22	1.76	2.64	1.23
*Aragonia aragonensis* (Aara)	3.69	0.47	0.28	2.90	3.45	1.89	2.46	0.00	0.05
*Bolivina huneri* group (Bhun)	0.00	3.34	0.01	0.00	0.00	1.43	0.78	0.00	0.00
*Boli*. cf. *decoratus* group (Bdec)	5.99	0.00	12.52	1.86	1.10	0.00	0.00	12.89	0.00
*Bulimina elongata* group (Belo)	0.43	5.75	0.03	0.21	0.00	0.70	3.47	0.62	0.00
Costate bulim. group (CoBul)	0.65	1.06	0.00	3.42	0.47	0.01	2.03	0.00	0.87
Fusiform bulim. group (FuBul)	0.00	4.07	3.03	2.69	7.06	4.32	3.10	6.68	0.00
*Bulimina simplex* group (Bsim)	1.08	2.28	0.98	6.00	4.08	1.37	6.04	4.66	6.55
*Bulimina trinitatensis* (Btri)	0.98	0.47	0.70	2.38	3.14	0.27	1.15	2.02	2.30
*Bulimina virginiana* (Bvir)	0.00	5.80	0.22	33.95	0.00	0.00	0.00	0.00	0.00
*Bulim*. *beaumonti* group (Bbea)	2.30	0.00	0.04	0.21	2.35	0.00	0.15	0.00	0.00
*Buliminella grata* (Bgra)	0.00	3.11	0.00	0.00	0.00	0.03	0.29	0.00	0.00
*Cib*. *mundulus* group (Cmun)	2.97	9.28	0.60	0.10	0.00	1.71	1.14	0.16	0.00
Small *Cib*./*Anom*. group (SmCib)	0.00	0.60	4.67	0.10	0.00	0.95	0.11	0.00	0.00
*Cib*. *subspiratus* (Csubs)	0.00	0.01	0.00	0.00	0.00	0.00	0.00	0.00	9.10
*Cib*. *eocaenus* group (Ceoc)	0.24	0.00	1.96	1.45	0.63	0.01	0.04	0.00	4.25
*Clinap*. *complanata* (Ccom)	0.00	0.00	0.01	0.00	0.00	5.34	4.19	0.00	0.00
*Clinapertina inflata* (Cinf)	0.53	0.10	1.94	1.45	2.98	2.06	1.92	0.00	0.00
*Epistominella exigua* (Eexi)	0.00	8.60	0.00	0.00	0.00	2.87	0.37	0.00	0.00
*Fursen*. *fusiformis* group (Ffus)	0.12	0.00	0.00	0.00	0.00	2.73	3.91	2.33	0.00
*Glob*. *subglobosa* group (Gsub)	1.37	6.36	5.07	3.93	4.55	2.16	2.72	0.47	0.92
Flat *Gyroidinoides* group (FlGyr)	1.52	1.93	6.90	1.13	2.67	0.37	0.60	0.00	0.00
*Hanzawaia ammophila* (Hamm)	3.28	0.00	0.00	0.00	0.00	0.00	0.00	0.00	0.00
Lenticulinids (Lent)	5.42	0.09	0.18	0.31	1.57	0.07	2.79	1.55	9.67
*Nonion havanense* group (Nhav)	1.05	1.06	0.64	0.10	2.20	2.23	1.75	3.88	0.00
*Nonionella robusta* (Nrob)	0.05	0.00	0.00	0.00	0.47	2.32	1.58	0.78	0.00
*Nuttallides truempyi* (Ntru)	13.91	1.72	14.06	4.66	13.19	11.08	10.65	10.56	6.96
*Nutt*. *umbonifera* group (Numb)	2.45	9.49	7.60	3.21	2.20	5.92	1.10	0.93	0.00
*Oridorsalis umbonatus* (Oumb)	3.62	1.76	5.27	0.83	4.55	4.36	4.71	2.48	13.40
Pleurostomellids (Pleu)	6.02	2.63	1.14	1.86	4.08	5.74	6.09	6.06	0.15
Polymorphinids (Poly)	2.28	0.07	0.39	0.31	0.94	1.76	1.56	1.40	0.31
Triangular bulim. group (TrBul)	3.33	0.00	0.68	0.82	0.32	0.00	0.00	0.00	0.00
*Quadrim*. *profunda* (Qpro)	0.96	0.42	6.89	2.28	8.16	8.72	1.02	1.24	0.00
*Seabrookia rugosa* (Srug)	1.61	0.24	0.01	0.00	0.94	1.55	6.48	0.00	0.00
*Sip*. *brevispinosa* group (Sbre)	0.60	0.34	0.00	0.00	0.16	1.71	1.00	6.68	0.00
Stilostomellids (Stil)	14.03	3.47	0.37	1.66	4.24	1.67	4.91	7.14	0.20
*Tappanina selmensis* (Tsel)	0.12	0.32	3.72	1.55	0.94	3.81	0.39	15.84	0.00
Uniserial lagenids (UnLag)	7.24	0.80	0.34	0.52	1.88	1.11	3.86	2.17	0.82

## 3. Results

### 3.1 Taxonomic remarks

**Fig 4:**
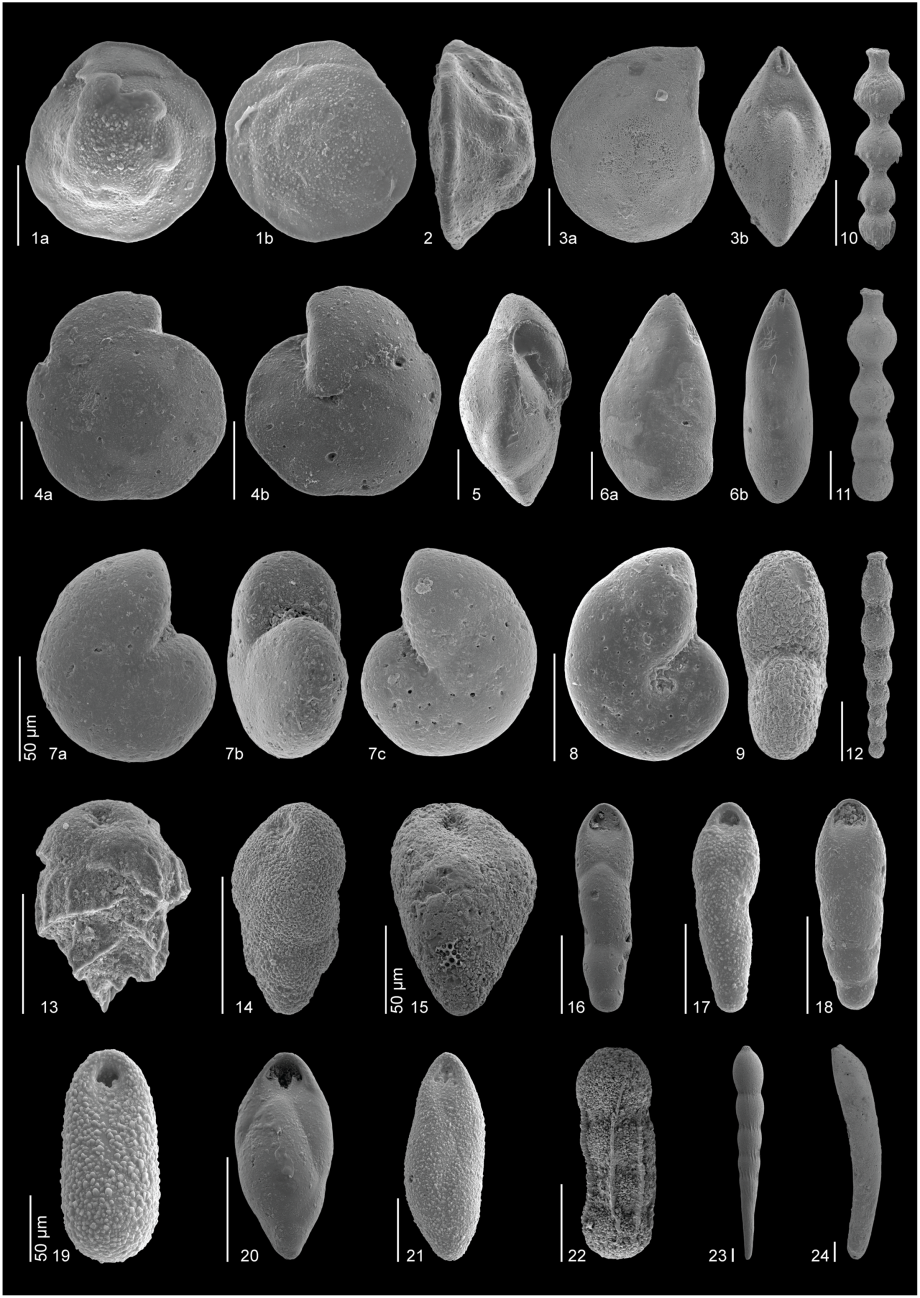
SEM images of some ubiquitous Ypresian (lower Eocene) species. All scale bars represent 100 μm, unless otherwise indicated. 1, *Nuttallides truempyi* (sample 865B-10-4, 120–125 cm): a) ventral, b) dorsal. 2, *Nuttallides truempyi* (sample 865B-10-5, 111–116 cm): apertural. 3, *Lenticulina* sp. (sample 865B-10-4, 120–125 cm): a) lateral, b) apertural. 4, *Oridorsalis umbonatus* (sample 865C-12-4, 6–8 cm): a) dorsal, b) ventral. 5, *Oridorsalis umbonatus* (sample 865C-12-4, 6–8 cm): apertural. 6, *Astacolus* sp. (sample 550-29-1, 8–10 cm): a) lateral, b) apertural. 7, *Anomalinoides praespissiformis* (sample 401-13-4, 130–132 cm): a) dorsal, b) apertural, c) ventral. 8, *Anomalinoides ammonoides*/*spissiformis* (sample 550-29-4, 145–147 cm): ventral. 9, *Anomalinoides ammonoides*/*spissiformis* (sample 550-29-1, 52–54 cm): apertural. 10–12, Stilostomellids: 10, *Siphonodosaria lepidula* (sample 550-29-5, 126 cm), 11, *Siphonodosaria pomuligera* (sample 401, 12-CC), 12, *Strictocostella matanzana* (sample 401-13-1, 91.5–92.5 cm). 13, *Bulimina trinitatensis* (sample 690-8A). 14, *Bulimina simplex* (sample 550-29-4, 65–67 cm). 15, *Bulimina tuxpamensis* (sample 1051-48-2, 135–137 cm). 16–21, Pleurostomellids: 16, *Pleurostomella* sp. 2 (sample 1258-14-4, 44–46 cm), 17, *Pleurostomella* sp. B (sample 865B-10-3, 4–6 cm), 18, *Pleurostomella* sp. 2 (sample 550-29-4, 35–37 cm), 19, *Pleurostomella* sp. D (sample 865B-10-3, 4–6 cm), 20, *Pleurostomella acuta* (sample 1258-14-4, 14–16 cm), 21, *Pleurostomella* sp. A (sample 865B-10-3, 60–62 cm). 22–24, Uniserial lagenids: 22, *Chrysalogonium* sp. 2 (sample 550-29-4, 95–97 cm), 23, *Chrysalogonium ciperense* (sample 550-29-4, 105–107 cm), 24, *Dentalina* sp. (sample 401-13-1, 84.5–85.5 cm).

**Fig 5:**
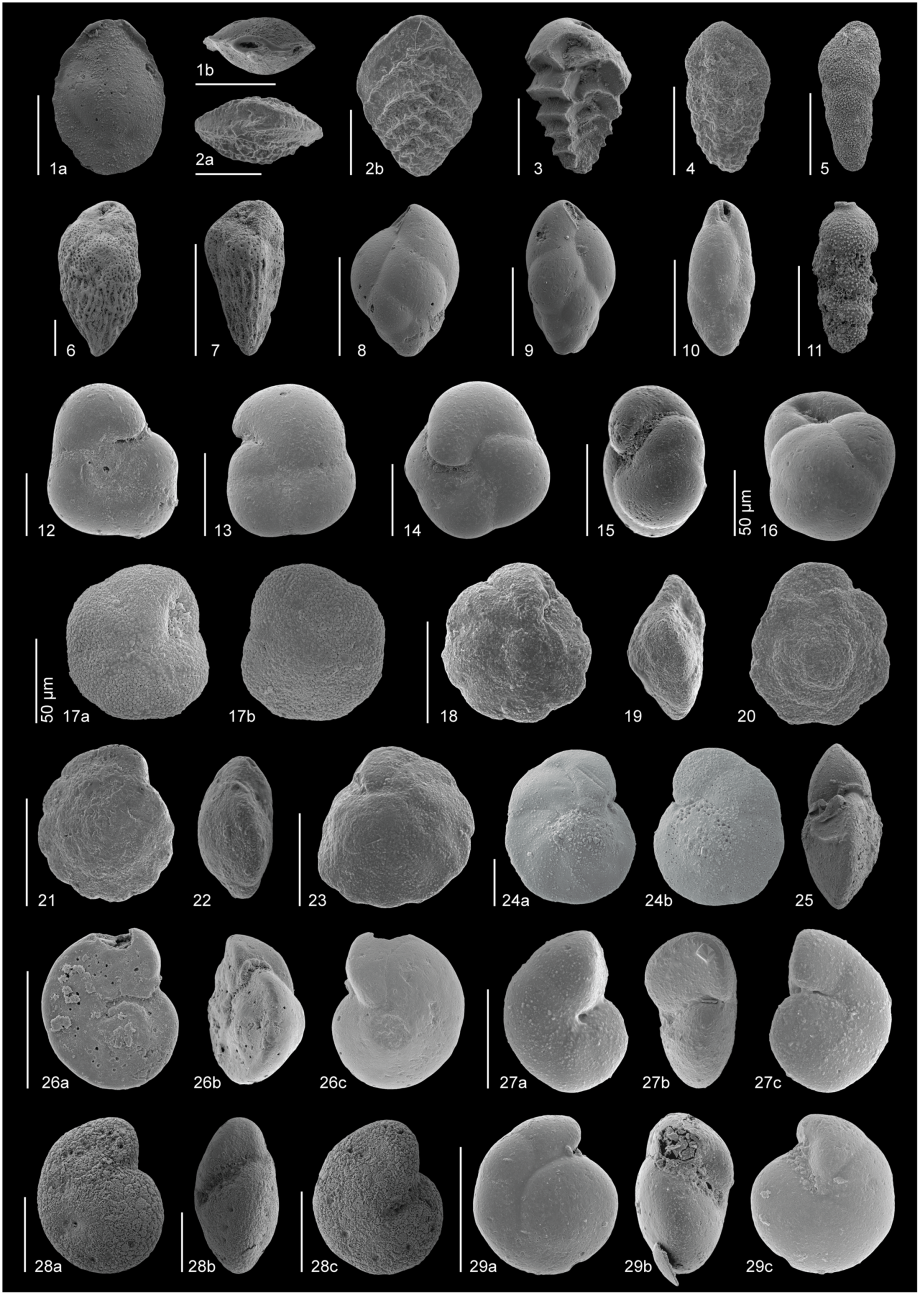
SEM images of some abundant Ypresian (lower Eocene) species. All scale bars represent 100 μm, unless otherwise indicated. 1, *Seabrookia rugosa* (sample 1258-14-4, 44–46 cm): a) lateral, b) apertural. 2, *Aragonia aragonensis* (sample 550-29-1, 8–10 cm): a) apertural, b) lateral. 3, *Tappanina selmensis* (sample 1258-14-4, 14–16 cm). 4, *Bolivinoides decoratus* (sample 550-29-4, 15–17 cm). 5, *Bulimina elongata* (sample 550-29-1, 52–54 cm). 6, *Bulimina semicostata* (sample 1051-48-1, 37–39 cm). 7, *Bulimina virginiana* (sample 1051-48-2, 75–77 cm). 8, *Bulimina* sp. 1 (sample 1258-14-4, 14–16 cm). 9, *Bulimina* sp. 1 (sample 1258-14-4, 14–16 cm). 10, *Bulimina kugleri* (sample 550-30-1, 106.5–108 cm). 11, *Siphogenerinoides brevispinosa* (sample 1258-14-CC, 5–7 cm). 12, *Quadrimorphina profunda* (sample 401-13-2, 43–45 cm): umbilical. 13, *Quadrimorphina profunda* (sample 550-29-5, 126.5–129 cm): ventral. 14, *Abyssamina poagi* (sample 550-30-1, 106.5–108 cm): umbilical. 15, *Abyssamina poagi* (sample 401-13-3, 43–45 cm): apertural. 16, *Globocassidulina subglobosa* (sample 550-29-6, 57–59 cm). 17, *Epistominella exigua* (sample 401-13-4, 130–132 cm): a) ventral, b) dorsal. 18, *Nuttallides umbonifera* (sample 550-29-5, 15–17 cm): ventral. 19, *Nuttallides umbonifera* (sample 550-29-5, 15–17 cm): apertural. 20, *Nuttallides umbonifera* (sample 550-29-1, 8–10 cm): dorsal. 21, *Osangularia* sp. 1 (sample 550-29-5, 15–17 cm): dorsal. 22, *Osangularia* sp. 1 (sample 550-29-4, 65–67 cm): apertural. 23, *Osangularia* sp. 1 (sample 550-29-2, 53–55 cm): ventral. 24, *Cibicidoides praemundulus* (sample 865-10-2, 4–6 cm): a) umbilical, b) dorsal. 25, *Cibicidoides praemundulus* (sample 865-10-2, 4–6 cm): apertural. 26, *Cibicidoides proprius* (sample 401-13-4, 130–132 cm): a) dorsal, b) apertural, c) ventral. 27, *Gyroidinoides depressus* (sample 865-10-2, 60–62 cm): a) ventral, b) apertural, c) dorsal. 28, *Gyroidinoides planulatus* (sample 550-29-1, 52–54 cm): a) dorsal, b) apertural, c) ventral. 29, *Gyroidinoides complanata* (sample 401-13-4, 130–132 cm): a) dorsal, b) apertural, c) ventral.

**Fig 6:**
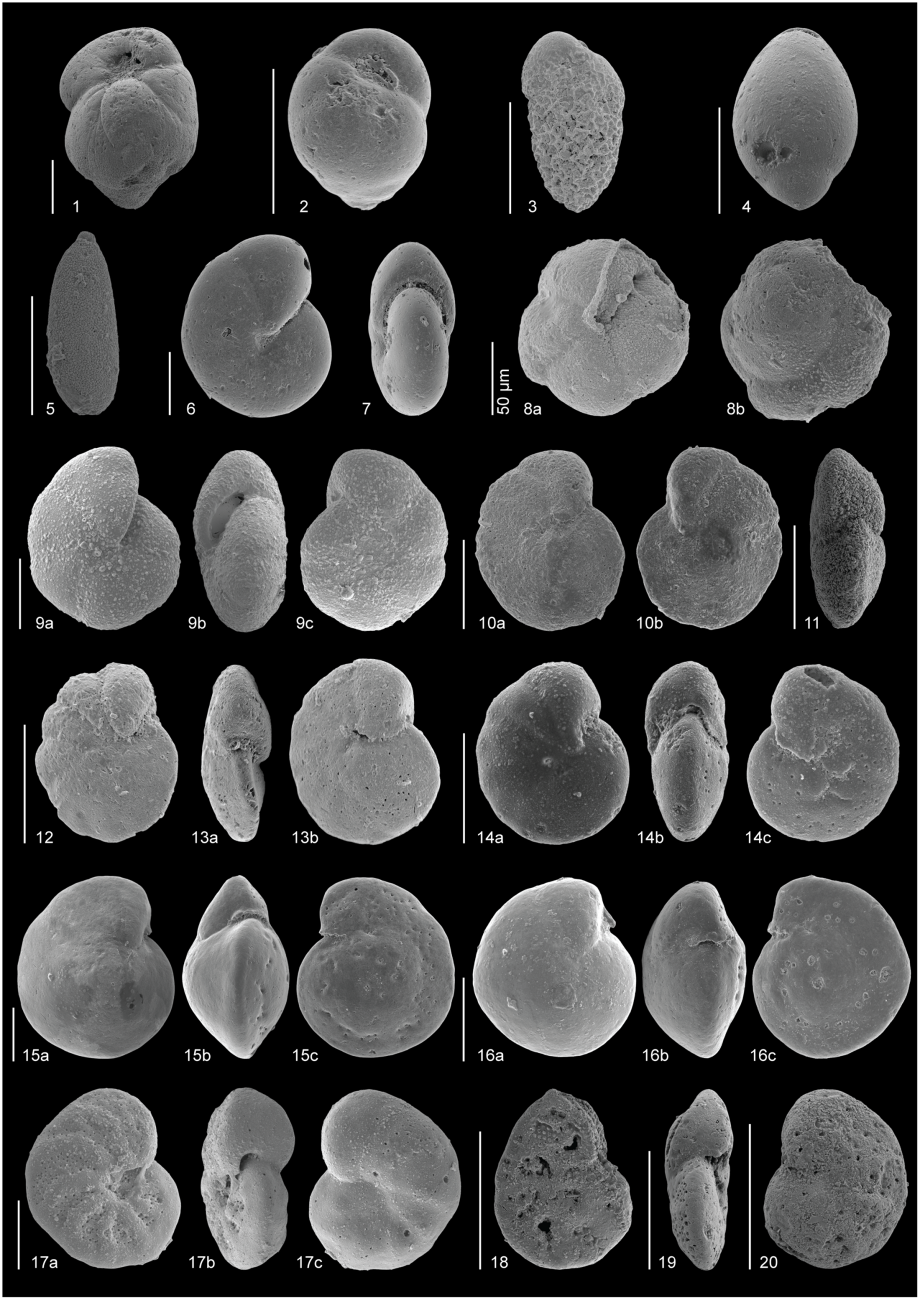
SEM images of some common Ypresian (lower Eocene) species. All scale bars represent 100 μm, unless otherwise indicated. 1, *Buliminella beaumonti* (sample 1258-14-2, 76–78 cm). 2, *Turrilina brevispira* (sample 401-13-2, 75–76 cm). 3, *Bolivina huneri* (sample 401-13-4, 130–132 cm). 4–5, Polymorphinids: 4, *Ellipsoglandulina* sp. (sample 1258-14-3, 104–106 cm), 5, *Pyrulinoides acuminatus* (sample 401-13-1, 125–126 cm). 6, *Nonion havanense* (sample 401-13-2, 75–76 cm): lateral. 7, *Nonion havanense* (sample 401-13-2, 75–76 cm): apertural. 8, *Alabaminella weddellensis* (sample 401-13-4, 130–132 cm): a) ventral, b) dorsal. 9, *Clinapertina inflata* (sample 865-10-2, 60–62 cm): a) ventral, b) apertural, c) dorsal. 10, *Cibicidoides micrus* (sample 550-29-5, 115–117 cm): a) dorsal, b) ventral. 11, *Cibicidoides micrus* (sample 550-29-6, 2–4 cm): apertural. 12, *Cibicidoides ungerianus* (sample 401-13-1, 88.5–90 cm): ventral. 13, *Cibicidoides ungerianus* (sample 401-13-2, 130–132 cm): a) apertural, b) dorsal. 14, *Anomalinoides* cf. *acutus* (sample 690C-15-3, 16–18 cm): a) ventral, b) apertural, c) dorsal. 15, *Cibicidoides eocaenus perlucidus* (sample 550-29-5, 95–97 cm): a) umbilical, b) apertural, c) dorsal. 16, *Cibicidoides eocaenus tuxpamensis* (sample 550-29-1, 110–112 cm): a) umbilical, b) apertural, c) dorsal. 17, *Hanzawaia ammophila* (sample 865-10-3, 60–62 cm): a) ventral, b) apertural, c) dorsal. 18, *Hanzawaia mantaensis* (sample 1051-48-1, 37–39 cm): dorsal. 19, *Hanzawaia mantaensis* (sample 1051-48-2, 75–77 cm): apertural. 20, *Hanzawaia mantaensis* (sample 1051-48-2, 75–77 cm): ventral.

The taxonomy of some of the most common early Eocene species (Figs 4–6), which have mostly been included in distinct informal taxonomic groups, is shown below. Full names including authors are given in the taxonomic list (S1 Appendix).

#### 3.1.1 *Bolivinoides* cf. *decoratus* group

This group includes species assigned to the genera *Bolivinoides* and *Bolivina* (Fig 5.4). Both genera are characterised by a biserial arrangement of broad and low chambers, but they differ mostly by the outline of their test. *Bolivina* has an ovoid to triangular outline, whereas *Bolivinoides* is rhomboidal in outline.

The *Bolivinoides* cf. *decoratus* group is based on the species originally identified as *Bolivina decorata* Jones, and includes specimens that were assigned to *Bolivinoides decorata* (at Site 865), *Bolivinoides decoratus* (at Sites 550, 690, 1051 and 1258) and *Bolivinoides* cf. *decoratus* (at Sites 689, 690, 1262 and 1263). According to the type description, *B*. *decorata* has an elongated test, broad at the apertural end and tapering to rounded point at the aboral extremity, and a surface ornamented with prominent, oblong tubercles arranged in oblique rows [112].

#### 3.1.2 *Bolivina huneri* group

As in the previous group, specimens assigned to the genera *Bolivina* and *Bolivinoides* are included in the *B*. *huneri* group (Fig 6.3). Its representative species, *Bolivina huneri*, was originally described by Howe [113], who described the small, elongated and only slightly compressed test, peripherically broadly rounded, with sides nearly parallel in the adult stage, and a wall ornamented with numerous, delicate and irregularly anastomosing costae, finely perforated.

D’haenens et al. [60] suggested the occurrence of intermediate forms between *B*. *huneri* and *Bolivinoides crenulata*. The latter species has a test about twice as long as broad, tapering with the greatest width near the apertural end, and is characterised by a wall ornamented by distinct, longitudinal ridges and a crenulated base of chambers, forming a pattern of irregularly rounded depressions. As far as we know, these transitional forms have been reported only at Site 401 during the early Eocene (biozones NP10-11) [60], and more studies are needed in order to determine whether they are rare and do not cover the full time range studied here, or if they have a more extended record. We have included in this group specimens named *Bolivina huneri* (at Site 550), *Bolivinoides huneri* (at Sites 1262 and 1263) and *Bolivinoides crenulata*/*Bolivina huneri* (at Site 401).

The species included in the *B*. *huneri* and *B*. cf. *decoratus* groups are somewhat similar, but can be distinguished based on the typical ornamentation of their tests. Although the ornamentation may be slightly blurred in some cases, *B*. *decoratus* does not show the pattern of irregularly rounded depressions as in *B*. *crenulata*, neither the irregularly anastomosing costae of *B*. *huneri*. For this reason, we allocated these species to different groups.

#### 3.1.3 *Fursenkoina fusiformis* group

This group includes species origninally recognised as *Virgulina fusiformis* by Cushman [114]. The type specimen is characterised by a small, somewhat compressed and fusiform test with the greatest width at about the middle portion of the test, and a bluntly pointed end. The arrangement of the inflated chambers in the type specimen is initially triserial, biserial in the later portion of the test. The sutures are very slightly depressed, and the aperture is elliptical, very small, at the upper end of the test.

The name of the genus *Virgulina* was deemed invalid because it was used to define a genus of a trematode worm, and *Fursenkoina* was proposed as the new name for this genus [115]. Species in this group have been assigned to the genus *Stainforthia*, possibly based on the arrangement of the earlier chambers. *Fursenkoina* is described as biserial throughout [116], but this is in disagreement with the original description of *V*. *fusiformis*. *Stainforthia* has chambers that are triserially arranged in the early stage, at least in the microspheric generation, followed by a twisted biserial arrangement [116]. Specimens identified in the literature as *Fursenkoina fusiformis* and *Stainforthia fusiformis* were included in this group.

#### 3.1.4 *Bulimina elongata* group

This group includes species with a triserial, long and slender, smooth test, including *Bulimina elongata* and *B*. *thanetensis* (Fig 5.5). These two species mainly differ in the longitudinal twist of the test in *B*. *thanetensis*. Close examination of several paratypes of *B*. *thanetensis* (CC 58889, CC 58890 and CC 58891, NMNH), however, showed that this twist is not clearly present in some specimens, which thus can be easily confused with *B*. *elongata*. *Bulimina thanetensis* may have become extinct during the early Ypresian, slightly after the start of the PETM (e.g., [14, 117]), whereas *B*. *elongata* continued into the early Eocene. Holbourn et al. [17], however, reported an extended stratigraphic range of *B*. *thanetensis* further into the Eocene.

The holotype of *B*. *thanetensis* (CC 35855, NMNH) is long and slender, with a markedly twisted test. The first chambers are very small, increasing slowly in size towards the middle part of the test, where they start to increase in size more rapidly, and are more inflated, thus the sutures are also more marked. The holotype slightly differs from deep-sea forms in its more rounded, squat chambers, and less-prominent sutures [17]. Typical long and slender tests of *B*. *thanetensis* have been found at Maud Rise (Site 690, e.g., [14, 17]), whereas the specimens called *B*. cf. *thanetensis* in Egypt are stouter (e.g., [117, 118]), and resemble those depicted by Tjalsma and Lohmann [11].

Neither the holotypes nor other primary types of *B*. *elongata* are deposited at the AMNH or NMNH, but we observed diverse Eocene specimens identified as *B*. *elongata* (CC 59037, CC 59030, CC 59031, CC 59026, CC 59036, CC 59028, CC 9545 and CC 59025, NMNH). We found a large variety in the width of the test, and in the length-width ratio. Only five specimens from slides CC 59031 and CC 59026 show truly long and slender tests with nearly parallel lateral sides, slightly inflated chambers and slightly depressed sutures. We also checked some hypotypes and plesiotypes from younger time intervals, but these specimens have a more ovate form and flush sutures, in contrast with the Eocene specimens. Nørvang [119] suggested that a trochospiral initial end of *B*. *elongata* occurs in the microspheric form, and thus these specimens do not correspond to the genera *Caucasina* as suggested by others [120].

Based on the specimen figured in D’haenens et al. [60], specimens here called *Bulimina* cf. *simplex* (originally named *Bulimina thanetensis*) at Site 401 (S3 Table) were included into this group because they have slightly parallel lateral sides (as in *B*. *elongata*), but do not have a truly long test. However, they do not have the bulging and protruding chambers which are typical for *B*. *simplex* (see *B*. *simplex* group).

#### 3.1.5 *Bulimina simplex* group

This group includes buliminids characterised by a very simple, smooth and straight test, rounded in cross section (e.g., *Bulimina simplex*, *B*. *bradburyi* and *B*. *tuxpamensis*), and without ornamentation or other specific features (Fig 4.14 and 4.15). These species are commonly confused in the literature due to the lack of clearly distinguishable features. Holbourn et al. [17] suggested that *B*. *bradburyi* and *B*. *tuxpamensis* are morphologically close, and that they may be conspecific.

According to the original description of *B*. *simplex*, the chambers from the first three spires are flat, and the last ones bulging and protruding [121]. On the contrary, *B*. *bradburyi* has inflated chambers [122], and they are broader than in *B*. *simplex*. In contrast to the sutures in *B*. *tuxpamensis*, in *B*. *bradburyi* they are distinct and depressed. *Bulimina tuxpamensis* has a very stout test, regular in outline, with wide and limbate, not depressed sutures [123]. This author pointed out that some specimens of *B*. *tuxpamensis* tend to become slightly three sided, but in our observations this species can be easily distinguished from buliminids with truly triangular cross-section, such as *Bulimina prolixa* and *Pyramidina rudita* (see below).

We noted the above differences between *B*. *bradburyi* and *B*. *tuxpamensis* during the examination of the paratypes of the first species (CC 38962, CC 559449, NMNH) and the plesiotypes of the second one (CC 23852, CC 62507 and USNM 623763, NMNH). However, the plesiotypes of *B*. *simplex* (CC 56845, CC 42645 and CC 24625, NMNH) are in general more slender than *B*. *bradburyi* and *B*. *tuxpamensis*, and show a broad variety of test-shapes. Some have a narrow test at the initial end, with greatest width at the apertural end, whereas others have a more fusiform test. The shape of the chambers varies, from square-rounded and inflated, to longer and more inflated.

#### 3.1.6 Triangular buliminid group

The group contains triserial species with blunt-edged triangular cross-section, without costae, e.g., *Bulimina prolixa* and *Pyramidina rudita*. Other Ypresian buliminid species such as *B*. *semicostata*, *B*. *virginiana*, *B*. *farafraensis* and *B*. *alazanensis* also have bluntly-edged triangular cross-sections, but they can be easily distinguished by their costate ornamentation.

Based on the original description of Cushman and Parker [124], *B*. *prolixa* is longer and narrower than *P*. *rudita*, and its sutures are slightly depressed. We examined several paratypes of *B*. *prolixa* (CC 32707 and CC 32708, NMNH). The specimens have a slender test with triangular cross-section, although they may vary from a typical buliminid shape (largest width close to last chamber) to somewhat fusiform (largest width closer to mid-test).

In contrast, *P*. *rudita* has a rougher surface but smaller test, with distinct, depressed and sigmoidal sutures [124]. This species is characterised by distinctly concave sides, as observed in several specimens from two slides at the NMNH (CC 32719 and CC 32723). Georgescu et al. [125] emended this species based on the pore mounds of the test and assigned it to the genus *Pseudouvigerina*. According to these authors, this genus includes turrilinids with pore mound ornamentation, but their illustrated specimens have a more elevated and pyramidal apertural face than the specimens in Ypresian samples. Moreover, *P*. *cristata* [126], the type species of *Pseudouvigerina*, has an aperture surrounded by a neck (e.g., [127]), in contrast with the loop-shaped aperture of the genera *Bulimina* and *Pyramidina*. The original description by Cushman [128] points out that *Pseudouvigerina* is characterised by a biserial early stage, becoming triserial later in its development, whereas Loeblich and Tappan [116] mention that the test is triserial throughout, as also seen in the micro-CT scan of *Pseudouvigerina* sp. in Speijer et al. [129]. We did not observe an early biserial stage or an aperture surrounded by a neck in our specimens, neither in the SEM images of *Pyramidina rudita* hypotypes in Georgescu et al. [125], and therefore disagree with Georgescu et al. [125], and retain *P*. *rudita* in *Pyramidina*.

#### 3.1.7 Costate buliminid group

This group includes *Bulimina semicostata* and *B*. *jarvisi*, which have a test with coarse perforations and irregularly-shaped, in some cases anastomosing costae (Fig 5.6). Van Morkhoven et al. [9] pointed out that these species are difficult to distinguish, and Tjalsma and Lohmann [11] suggested that transitional forms may occur in the middle Eocene (biozone P14).

The typical *B*. *jarvisi* is longer, and its greatest diameter occurs approximately at two thirds of the distance from the proloculus, whereas *B*. *semicostata* has a blunter triangular cross-section, a greatest diameter at the penultimate chamber, and an imperforate area extending from the aperture to the final chambers [9]. Based on our observations of the holotype of *B*. *jarvisi* (CC 23128, NMNH) and cotypes of *B*. *semicostata* (CC 51871 and CC 59482, NMNH), *B*. *jarvisi* is narrower and longer than *B*. *semicostata*, and has more inflated chambers and more depressed sutures, in agreement with the original description of Cushman and Parker [130]. The costae in *B*. *jarvisi* proportionally cover a larger area of the test than in *B*. *semicostata*. This ornamentation is finer in *B*. *jarvisi* than in *B*. *semicostata* [17].

Additional species that may be related to this group include *B*. *asperoaculeata* and *B*. *glomarchallengeri*. *Bulimina asperoaculeata* (originally spelled as *aspero-aculeata*) shows less distinctive costae and deeper sutures than *B*. *jarvisi* [131]. The microspheric form of *B*. *semicostata* may be confused with *B*. *glomarchallengeri*, which also slightly resembles the juvenile specimens of *B*. *callahani*, but *B*. *glomarchallengeri* has more inflated chambers. According to Olshanetskiy [132], *B*. *glomarchallengeri* had its first appearance during the late Eocene, later than the range of *B*. *semicostata*.

Other Ypresian buliminids with costate tests (e.g., *B*. *virginiana*, *B*. *farafraensis* and *B*. *alazanensis*) are not included in this group because they can be easily distinguished from *B*. *semicostata* or *B*. *jarvisi* based on the general morphology of their test. For example, the hypotype of *B*. *virginiana* (USNM 139271, NMNH) has a small, narrow test, clearly longer than wide, triangular in cross-section with concave sides and fine costae over the whole test. Some specimens of *B*. *virginiana* from ODP Site 1051 and DSDP Site 550 have flat rather than concave sides. Both *B*. *alazanensis* and *B*. *farafraensis* have a somewhat larger width/length ratio, sometimes with a small spinose projection at the initial end, but the former species has prominent, continuous and more pronounced longitudinal costae, whereas *B*. *farafraensis* (junior synonym of *B*. *aksuatica* according to Deprez et al., [98]) shows numerous long, well-defined costae. Alegret and Ortiz [117] pointed out that *B*. *farafraensis* may show more irregular costae than the holotype, ocassionally branching. According to LeRoy [133], *B*. *farafraensis* can be distinguished by its rounded triangular cross-section and uninterrupted longitudinal costae, which we clearly observed on the holotype (CC 58013, NMNH).

#### 3.1.8 Fusiform buliminid group

This group has a complex record in the taxonomic literature, and includes buliminids with a typical fusiform shape, smooth test, inflated chambers and depressed sutures, resembling *Praebulimina reussi* (more inflated form) and *Bulimina kugleri* (slender form; Fig 5.10). According to Hofker [134], the genera *Praebulimina* and *Bulimina* are distinguished by the complexity of the inner tooth-plate structure linked to the aperture in *Bulimina*, whereas the toothplate in *Praebulimina* is rather simple. This author thought that *Praebulimina* might be considered ancestral to *Bulimina*, if the toothplate is considered the most typical characteristic of these genera. It has been suggested that *P*. *reussi* (with its typical subterminal, comma-shaped aperture) was common in the Maastrichtian and became extinct at or close to the Cretaceous/Paleogene boundary globally [135, 136], although Sprong et al. [137] reported it from Danian strata. The subtle differences between the genera *Praebulimina* and *Bulimina*, however, have not been clearly described in the literature, and not been observed by all authors.

The species *B*. *kugleri* is characterised by a marked fusiform shape, with long inflated chambers, and the greatest width in the middle part of the test, with an aperture characterised by a high, arched, curved opening at the base of the inner margin of the last chamber [138]. The holotype of this species (CC 38199, NMNH) clearly shows these features, but we found considerable variability in the fusiform shape in the nineteen paratypes observed (CC 38257, NMNH), as also observed by Thomas [139]. Some of these specimens have the greatest width in the middle part of the test, but with a broader end part, so that the general form is closer to that of a typical buliminid. Additionally, some paratypes have a smaller width-to-length ratio than the holotype and specimens from the Southern Ocean Sites 689 and 690 [139]. This author pointed out that the specimens from the Southern Ocean closely resemble *B*. *kugleri*, but that they are smaller than the type specimens.

Chambers of *P*. *reussi* rapidly flare from the pointed initial part, and the test has its broadest part in the last whorl [140]. *Praebulimina reussi* has a subterminal, comma-shaped aperture. We observed three plesiotypes, one of which (CC 39602, NMNH) has a markedly pointed initial part with small chambers that increase gradually in size, but the chambers rapidly increase in size at approximately one third of the test, resulting in very inflated chambers. The initial part of the other two plesiotypes (CC 22433 and CC 51863, NMNH) is less pointed, the chambers do not abruptly increase in size, and the greatest width occurs in the middle part of the test. We observed that the last chambers of *P*. *reussi* are occasionally elongate and inflated, resembling *B*. *kugleri*. Similarly, Alegret and Thomas [10] suggested that *P*. *reussi* may vary in elongation of the test, ranging from low and globular to elongate and fusiform. In the latter case, these two species may be distinguished because *B*. *kugleri* does not show the small, low and compressed early chambers of *P*. *reussi*.

The identification of these species is problematic, and they have been confused with each other, or misidentified in the literature. For example, Pardo et al. [141] may have misidentified *P*. *reussi* as the species *Praebulimina carseyae* in their Maastrichtian material [142]. The specimens determined by White [21] as *B*. *ovula* Reuss were later renamed *P*. *reussi* (Morrow) (e.g., [16, 30]) because *Bulimina ovula* Reuss was an invalid name, being a homonym of *B*. *ovula* d’Orbigny [140]. There thus has been considerable confusion between specimens named *B*. *ovula* Reuss and *B*. *ovula* d’Orbigny, the latter being an extant form with a much more inflated test. Another homonym is *Bulimina ovula* Terquem, which is now placed in the genus *Buliminella* because it has more than 3 chambers per whorl. Some authors consider *Bulimina ventricosa* [143] as synonym with *P*. *reussi* (e.g., [144]).

In addition to *P*. *reussi* and *B*. *kugleri*, the fusiform buliminid group includes similar Ypresian specimens that have been assigned different names, such as *Bulimina* sp. 1 (Fig 5.8 and 5.9), *Bulimina kugleri* (*ovula*), *Bulimina trihedra*, *Fursenkoina* sp. 2 and *Fursenkoina* sp. 3.

#### 3.1.9 *Globocassidulina subglobosa* group

This group is integrated by the homonym species *Globocassidulina subglobosa* (Fig 5.16), which is recognised by its large, thick and subglobular test, with few chambers and an obliquely-set loop-like aperture on the ventral face of the terminal segment [145].

The group includes specimens identified as *Globocassidulina globosa* (Hanken) at Site 752 by Nomura [93], which according to the SEM image in that publication ([93]: Plate 5, Figure 16) correspond to *G*. *subglobosa*.

#### 3.1.10 *Pullenia jarvisi* group

We included into this group the species *Pullenia jarvisi*, *P*. *subcarinata* and *P*. *americana*, although their original descriptions indicate they are different species. *Pullenia jarvisi* has a planispiral test, which is completely involute, biumbilicate and somewhat depressed, with a lobulate outline and about five slightly inflated chambers, separated by depressed sutures. The aperture consists of a low slit extending from one umbilicus to the other, with a strongly convex apertural face [146]. *Pullenia subcarinata* has a planispiral convex test with a non-lobate outline, slightly keeled, and convex umbilicus [147]. This species shows six chambers, its sutures are not depressed, and the upper part of the last chamber is not convex. The type specimen of *P*. *americana*, which is actually a junior synonym of *Pullenia quinqueloba* Cushman (not Reuss), has a planispiral, completely involute, much compressed and slightly umbilicate test, peripherally rounded. *Pullenia americana* has 5 to 6 somewhat inflated chambers, separated by slightly depressed and curved sutures, and an elongate aperture at the base of the apertural face, low at the sides and considerably higher in the middle [146]. We think that *P*. *subcarinata* and *P*. *americana* have commonly been confused and misidentified in the literature, despite these differences. For example, the SEM images of *P*. *subcarinata* in Nomura and Takata [37] resemble *P*. *jarvisi* because of their slightly lobate outline, somewhat depressed sutures and a last whorl with five chambers. Additionally, the upper part of the last chamber is not acute, as in the type figure of *P*. *subcarinata*. On the other hand, we expected to observe a higher apertural face than depicted in the SEM image of the specimens named *P*. *americana* by Nomura [93], and we argue that those specimens are more similar to *P*. *jarvisi*. Due to inconsistencies in the identification of these specimens, we decided to include them into a single group, but other flat *Pullenia* species such as *P*. *quinqueloba* (Reuss) and *P*. *salisburyi* are excluded as they may be distinguished from this group mostly by their higher apertural face. Likewise, synonyms of the species that we considered misidentified should be detached from this group (e.g. *P*. *quinqueloba*).

In addition, we found that many specimens assigned to *P*. *jarvisi* in Arreguín-Rodríguez and Alegret [63], and *Pullenia* sp. 1 in Alegret and Thomas [148] do not belong in the genus *Pullenia*, because they are not truly planispiral, but should be assigned to *Clinapertina subplanispira*. Our data from DSDP Site 550 have been modified accordingly in the S5 Table, and the percentages have been recalculated.

#### 3.1.11 *Abyssamina incisa*-*poagi* group

This group includes *Abyssamina incisa* and *A*. *poagi* (Fig 5.14 and 5.15). Both species are distinctly trochospiral, with the last chamber extending over one side. They closely resemble each other, as both species have been described as having four chambers in the last whorl and a lateral chamber expansion on the ventral side [149]. Tjalsma and Lohmann [11] figured specimens of *Abyssamina poagi* with three chambers.

According to Schnitker and Tjalsma [149], *A*. *incisa* has an accentuated surface relief, and much greater inflation and length of the lobate extension of the last chamber, although very rare morphologically intermediate specimens may occur. We observed some of these intermediate or transitional specimens (e.g., in samples from DSDP Site 550), with the last chamber more pronounced than in the holotype of *A*. *poagi* (USNM 305101, NMNH) but less than in the holotype of *A*. *incisa* (USNM 305100, NMNH), and with the degree of depression of the sutures intermediate between both holotypes.

*Abyssamina quadrata* is somewhat similar to the species of this group, but it can be easily distinguished by its almost planispiral test. *Abyssamina quadrata* has slightly depressed sutures, as does *A*. *poagi* [149], and has four chambers in the last whorl, but is characterised by a quadrate test that is spheroidal in cross-section. Comparing the holotypes, we observed that the last chamber is more inflated in *A*. *quadrata* (USNM 305103, NMNH) than in *A*. *poagi*, but it does not look as pronounced as in *A*. *incisa*, because all chambers in *A*. *quadrata* are inflated. The globular outline of *A*. *quadrata* is clearly observed in the images shown by Holbourn et al. [17]. *Abyssamina poagi* differs from *A*. *quadrata* in its longer and narrower chambers, which tend to overlap in the umbilical region, by its more strongly curved umbilical sutures and by its shorter, wider aperture [9]. Schnitker and Tjalsma [149] pointed out that *A*. *quadrata* slightly resembles small and rounded specimens of *Pullenia jarvisi*, but the aperture in *A*. *quadrata* is asymmetrical, whereas it runs from umbo to umbo in *P*. *jarvisi*.

*Quadrimorphina profunda* also resembles the abyssaminid species, although this species has a low trochospiral test, compressed laterally, with 3^1^/2–4^1^/_2_ chambers in the last whorl. Its involute side greatly resembles a four-chambered *Abyssamina*. The main differences with the *Abyssamina* species are that *Q*. *profunda* is trochospiral, with a strongly evolute side (absent in the abyssaminids) that exposes earlier whorls, it has a peripheral aperture with a tendency for extending somewhat onto the evolute side, and it does not show an extension through the umbilical side, as in the abyssaminids. Thus, we did not include it into this group. The figured *A*. *poagi* specimen in Holbourn et al. [17] might, in fact, be a misidentified *Q*. *profunda*, in view of the shape of its last chamber extending over the evolute side.

#### 3.1.12 *Eilohedra weddellensis* group

*Eilohedra weddellensis* and *Alabaminella weddellensis* are generic assignments used for the species originally described as *Eponides weddellensis* (Fig 6.8). According to Earland [150], *Ep*. *weddellensis* has a minute biconvex test, with a rounded peripheral edge and an aperture characterised by a minute slit on the inner edge of the final chamber on the ventral side. This species has five chambers in the last whorl, separated by distinct, flush sutures on the dorsal side, and depressed on the ventral side.

The taxonomy of this species is not fully resolved, thus we are not able to designate the best fitting generic assignment, but here we point out the main differences among these genera. Specimens of the genus *Eponides* have a biconvex test with angular to carinate periphery. Neither *Eilohedra* nor *Alabaminella* show such a feature, instead they have a rounded periphery. Moreover, *Alabaminella* has a biconvex test, whereas *Eilohedra* has a flattened umbilical side and a convex spiral side [116], and they differ in the presence of an apertural plate-like lip in *Alabaminella* [151], and the thightly coiled test of *Eilohedra* [152].

#### 3.1.13 *Anomalinoides spissiformis* group

This group includes morphologically similar species that have been commonly confused in the literature, such as *Anomalinoides spissiformis*, *A*. *praespissiformis* and *A*. *praeacutus* (also called *A*. *praeacuta*) (Fig 4.7). The original description of *A*. *praespissiformis* states that it closely resembles *A*. *spissiformis*: the former is characterised by a smaller size, fewer chambers and more strongly curved sutures [153]. Our observations suggest that the size and number of chambers may vary within a species, thus these criteria do not appear to be sufficient to distinguish between species. For example, Tjalsma and Lohmann [11] mention that the specimens identified as *A*. *spissiformis* are smaller and more tightly coiled than the holotype. The curvature of the sutures may be a better criterion to distinguish these species, and we leave this possibility open for future studies.

According to the type description of *A*. *praeacutus*, its periphery is rounded, but the figure of the apertural view in the original description of Vasilenko [154] shows a more acute periphery on the first part of the last whorl. This feature is used by some to distinguish this species from the other two, although there is no agreement in the literature. For example, the specimens identified as *A*. *praeacutus* and *A*. *praespissiformis* by Nomura and Takata [37] and D’haenens et al. [60], respectively, resemble *A*. *spissiformis*. Due to the lack of consistent identification of these species, we included them in one group.

There is also confusion with the species *A*. *ammonoides*, which has been commonly identified in the Cretaceous and Paleocene (e.g., [10]), whereas morphologically similar forms have been identified as *A*. *spissiformis* in Eocene material (e.g., [155]). Due to the strong similarity between these two species, Arreguín-Rodríguez and Alegret [63] called their specimens from Site 550 *Anomalinoides ammonoides/spissiformis* (Fig 4.8 and 4.9). We suggest that these species may be synonyms, with the name *A*. *ammonoides* commonly being used for Cretaceous and Paleocene specimens, and *A*. *spissiformis* for Eocene specimens.

#### 3.1.14 *Anomalinoides capitatus* group

Specimens called *Anomalinoides capitatus*, *A*. *capitatus*/*danicus*, and *A*. *rubiginosus* are included into this group. The species commonly named *Anomalinoides danicus* (originally described as *Cibicides danica*) is a junior synonym of *Anomalinoides rubiginosus* [9, 10], characterised by a trochospiral, closely coiled test, rounded to somewhat lobulate on the periphery, chambers and sutures rather indistinct, 9 to 10 chambers in the last whorl and a very coarsely perforated wall on both sides. Van Morkhoven et al. [9] and Bolli et al. [7] pointed out that *A*. *rubiginosus* ranges from the Upper Cretaceous to the upper Paleocene, and Alegret et al. [38, 39] suggested that this species became extinct at the PETM. Speijer [99] and Holbourn et al. [17], however, mention a range for this species up into the lower Eocene.

*Anomalinoides capitatus* differs from *A*. *rubiginosus* in the imperforate periphery of the early chambers, fewer chambers (5–6) in the final whorl, which increase gradually in size, and in the marked sutures varying from thin and depressed in the final chambers to thick and raised in the earlier ones. Van Morkhoven et al. [9] considered that *A*. *rubiginosus*, *A*. *capitatus*, *A*. *semicribratus*, and *A*. *globosus* represent a chronocline from the Late Cretaceous to the present. Thus, the specimens called *A*. *capitatus*/*danicus* by Nomura [93] may correspond to transitional forms. Tjalsma and Lohmann [11] recognised some of these transitional forms in the upper Paleocene.

#### 3.1.15 Small *Cibicidoides*/*Anomalinoides* group

This group includes small trochospiral, somewhat compressed specimens assigned to the genera *Cibicidoides* and *Anomalinoides*, typically with an unequally biconvex to nearly planoconvex test with numerous (usually 12–15) chambers in the last whorl. After careful examination of the data from the study sites, we included *Cibicidoides micrus*, *Anomalinoides* cf. *acutus*, *Cibicidoides subcarinatus* and *C*. *ungerianus* into this group (Fig 6.10–6.14).

According to the description of Bermúdez [156], *C*. *micrus* has some protruding shell growth, usually a continuous, circular protuberance, at the center of the dorsal surface. We checked the holotype (CC 62431, NMNH) and two paratypes (CC 62432, NMNH) of *C*. *micrus*, and observed a depressed spiral suture that delimits a circular protuberance formed by a small edge-like extension at the base of the chambers. This feature is more clearly seen in the holotype, but difficult to observe in one of the paratypes, and it was not observed in the specimens identified by Arreguín-Rodríguez and Alegret [63] at Site 550.

After reviewing our material, we recognise that specimens called *C*. *micrus* at DSDP Site 550 and ODP Site 1051 closely resemble those called *A*. cf. *acutus* at ODP Sites 1262, 1263 and 690 [30, 62] and *Anomalinoides* sp. B in Cretaceous-Paleogene sediments at ODP Site 1262 [157], but these do not fully agree with the type description of *A*. *acutus* [158]. Furthermore, in view of the images of D’haenens et al. [60] and Nomura and Takata [37], we suggest that the specimens named *C*. *ungerianus* and *C*. *subcarinatus* respectively, are more similar to *C*. *micrus* or *A*. cf. *acutus* than to the type figures and descriptions of these taxa. *Cibicidoides ungerianus* has an acute and carinate periphery, but the specimens illustrated in D’haenens et al. [60] do not show a carinate periphery. *Cibicidoides subcarinatus* has an equally biconvex test with a subcarinate periphery and limbate, comma-shaped sutures, whereas the specimens figured by Nomura and Takata [37] show a more planoconvex test with comma-shaped but slightly depressed sutures.

#### 3.1.16 *Cibicidoides* with an umbo group

Species of this group are distinguished from other biconvex *Cibicidoides* by the elevations of shell material on the dorsal side, and the occurrence of an umbo on the ventral side. *Cibicidoides alleni* and *C*. *dayi* are included in this group.

The general morphology of both species is similar, but according to Alegret and Thomas [10] *C*. *dayi* has a strongly depressed spiral suture around a prominent umbo instead of a true elevation of shell material at the base of the chambers on the dorsal side; in addition, its umbo on the ventral side is rather indistinct. In the photographs of type specimens of *C*. *dayi* (AMNH-F1-19908 and AMNH-F1-19909, available at http://foraminifera.eu/amnh.php), an umbo on the ventral side may be observed only on specimens with a marked spiral suture, whereas the umbo is indistinct in other specimens.

Specimens identified as *Anomalinoides trinitatensis* by Takeda and Kaiho [36] are included in this group because they closely resemble the species included in this group (*C*. *alleni* and *C*. *dayi*). In the original description of Nuttall [159], *A*. *trinitatensis* also shows a growth of shell material on the dorsal side and a boss on the ventral side, but it has a more planoconvex test. However, specimens in Takeda and Kaiho [36] differ from *A*. *trinitatensis*, in having fewer but more inflated chambers, than those in the type figure, and there is no umbo on the ventral side.

*Cibicidoides micrus* also has a depressed spiral suture on the dorsal side, but it is not included into this group because of its smaller size and flatter test, and it was included in the group of small *Cibicidoides/Anomalinoides*.

#### 3.1.17 *Cibicidoides mundulus* group

This group consists of trochospiral species with a morphology similar to that of species such as *Cibicidoides mundulus*, *C*. *praemundulus*, *C*. *pseudoperlucidus* and *C*. *proprius* (Fig 5.24–5.26). All have a planoconvex or unequally biconvex to lenticular test, with a plug on the umbilical side and a coarsely perforate wall.

*Cibicidoides mundulus* and *C*. *praemundulus* have lenticular tests with an imperforate band along the periphery, 3 whorls with 10–12 chambers in the last one, and a plug or umbo. According to Van Morkhoven et al. [9], they differ because *C*. *praemundulus* is smaller and more compressed, and does not have pores concentrated along the spiral sutures as in *C*. *mundulus*. Van Morkhoven et al. [9] and Holbourn et al. [17] showed that the degree of curvature in the sutures, the pattern of pores and the test size of *C*. *mundulus* are variable, and they considered *C*. *kullenbergi* a junior synonym of *C*. *mundulus*.

The species *C*. *proprius* is more planoconvex, and *C*. *pseudoperlucidus* is biconvex but with a somewhat flattened ventral side. Brotzen [131] described that *C*. *proprius* may vary in convexity, from planoconvex to biconvex. These two species have a slightly lobate, acute periphery, and a finely perforate wall. *Cibicidoides mundulus* and *C*. *praemundulus* and these two species have a plug or boss, and have 3 whorls, but *C*. *pseudoperlucidus* and *C*. *proprius* usually have fewer chambers in the last whorl than the other two species. Berggren and Aubert [160] suggested that *C*. *proprius* and *C*. *alleni* might be considered as synonyms, arguing that the variation in convexity of the test (*C*. *proprius*-planoconvex, *C*. *alleni*-biconvex) is not a differentiating feature of these two species. However, Alegret and Thomas [10] documented that *C*. *alleni* also differs from *C*. *proprius* in the calcite ridge along the spiral side, and in the lack of an acute periphery as in the latter species. For the purpose of this study, we consider these two species (*C*. *alleni* and *C*. *proprius*) sufficiently distinct to be separated into different groups (*C*. *alleni* is included in the *Cibicidoides* with an umbo group, and *C*. *proprius* in the *C*. *mundulus* group).

Specimens called *Cibicidoides eocaenus* in D’haenens et al. [60] were re-named as *Cibicidoides howelli* (S3–S5 Tables). *Cibicidoides howelli* may have become extinct during the PETM (e.g., [38]). Alegret and Thomas [10] suggested that 2 paratypes of *C*. *howelli* (CC 38529), deposited at the NMNH, should be included in *C*. *proprius*, because they lack the rounded last portion of the test and have an acute periphery. Based on the SEM images in D’haenens et al. [60], we suggest that these specimens (originally called *C*. *eocaenus*) should be included in *C*. *proprius*, due to their resemblance to the paratypes of *C*. *howelli* described by Alegret and Thomas [10], and thus are incorporated in the *C*. *mundulus* group.

#### 3.1.18 *Cibicidoides eocaenus* group

The species *Cibicidoides eocaenus*, *C*. *tuxpamensis* and *C*. *perlucidus* are included in this group. The test of *C*. *eocaenus* is small, nearly circular and unequally biconvex (may vary from planoconvex to biconvex), its umbilical side is somewhat cone-shaped, and the spiral side is somewhat less convex. The chambers (12–15) are inflated, separated by curved and limbate sutures. Additionally, *C*. *eocaenus* has a distinct spiral suture line and frequently a prominent umbilical umbo [9]. In spite of the latter feature, *C*. *eocaenus* is not included in the *Cibicidoides* with an umbo group because of its characteristic cone-shaped umbilical side, which makes them easy to distinguish.

Van Morkhoven et al. [9] compared topotype specimens and suggested that the species *C*. *eocaenus* and *C*. *tuxpamensis* may be conspecific. The latter species is very similar to *C*. *perlucidus*, characterised by its umbilical plug and acute periphery, but they differ because *C*. *tuxpamensis* has a more rounded periphery. According to these authors, there are gradational variations between these two species, which may be considered as ecophenotypic variants and the names *perlucidus* and *tuxpamensis* may be used to refer to the periphery of each morphotype. Following this criterion, Arreguín-Rodríguez and Alegret [63] used the names *C*. *eocaenus perlucidus* and *C*. *eocaenus tuxpamensis* to designate specimens with an acute and rounded periphery respectively at Site 550 (Fig 6.15 and 6.16). These names have also been applied in the classification of benthic foraminifera at Sites 1051 and 1258 (this study), and these species have been included here into the *C*. *eocaenus* group.

#### 3.1.19 Flat *Gyroidinoides* group

This group includes *Gyroidinoides* species characterised by compressed, flat to slightly biconvex tests and a rounded periphery such as *Gyroidinoides depressus*, *G*. *planulatus* (both alternatively assigned to *Valvalabamina*, e.g., [98, 99, 118]) and *G*. *complanata* (Fig 5.27–5.29). The small differences in e.g., the shape of the sutures, the degree of inflation of the chambers, and the number of chambers in the last whorl make these species difficult to distinguish.

*Gyroidinoides depressus* may have somewhat inflated later chambers and depressed sutures, whereas chambers in *G*. *planulatus* and *G*. *complanata* are not inflated, thus their sutures are not depressed. The latter two species may differ in the shape of the sutures on the ventral side, which are slightly curved in *G*. *planulatus* [161] and straight and nearly radial in *G*. *complanata* [162].

#### 3.1.20 *Nuttallides umbonifera* group

The original description of *Nuttallides umbonifera* defines a biconvex test with a boss in the umbo on the ventral side [163] (Fig 5.18–5.20), but specimens with a small boss, or even without a boss, have been commonly assigned to this species [120]. We reviewed the holotype of *N*. *umbonifera* (USNM 26162, NMNH), which has an unequally biconvex test, which is more convex on the ventral side, with an acute and slightly lobulate periphery [17]. On the dorsal side, the holotype is evolute, showing oblique to slightly curved sutures. On its ventral side, it shows an umbo, the chambers are trapezoidal in shape, and the sutures are oblique to curved, and depressed. The aperture is a slit that extends from the periphery to the umbo, with a fold perpendicular to the base of the last chamber.

Additionally, we observed specimens that resemble *N*. *umbonifera* (with an umbo), but in which the first chambers of the last whorl do not have a clearly trapezoidal shape on the ventral side, as in the type specimens, and with more chambers. These specimens have been called *Osangularia* sp. 1 at DSDP Site 550 [63] and at ODP Site 1051 (this study), although they are difficult to distinguish from the ‘typical’ *N*. *umbonifera* (Fig 5.21–5.23). According to Loeblich and Tappan [116], the aperture of *Osangularia* is T-shaped [164], but *Nuttallides* shows a ventral aperture at the base of the chamber, with a lip, extending from the umbilical boss to the periphery, formed parallel to the plane of coiling and bordered marginally by a small fold of the apertural face [165]. The apertural features, however, are not clearly seen in many specimens, especially small and/or poorly preserved ones, so that identification may be difficult. The variations in number and shape of chambers of *N*. *umbonifera* and the so-called *Osangularia* sp. 1 may represent interspecific variability of the same species (i.e., *N*. *umbonifera*).

The genus *Nuttallinella* may be distinguished from *Nuttallides* based on the presence of an umbilicus—rather than a boss—on the ventral side [166]. Cretaceous specimens similar to *N*. *umbonifera*, however, have been identified as *Nuttallinella ripleyensis* or *Nuttallinella* sp. at ODP Site 1262 [157]. These species have slightly less lobulate tests than *N*. *umbonifera*, and their sutures on the dorsal side are less oblique. Neither of these two species are included in this group because they are not present in the early Eocene.

Considering the general morphology of the test, and based on our observations as well as on the images of the Ypresian specimens in the literature, we suggest that specimens identified as *Nuttallides umbonifera*? (e.g., [14]), *Nuttallides* sp. 2 [37], *Osangularia* sp. 1 [63], *Osangularia* sp. [36] and *Eponides elevatus* [36] should be included in this group. They may be related species, represent intraspecific variability, or include several, morphologically not well-defined species.

#### 3.1.21 *Hanzawaia mantaensis*

In the studied material, this species has been found to be common only at ODP Site 1051. We examined several type specimens of *Hanzawaia mantaensis* at the AMNH (No. 19954), and were able to identify the holotype shown in the type figure, which is stored in the same slide as several syntypes (as *Anomalinoides mantaensis* at http://foraminifera.eu/amnh.php).

The holotype has a planoconvex test with an acute periphery. The dorsal side is flat and evolute. It shows numerous (usually 10) narrow chambers in the last whorl, separated by curved, limbate sutures. The ventral side is convex and involute, and the sutures are curved and depressed. According to the original description [167] and to Holbourn et al. [17], this species shows a boss on the ventral side (clearly seen in the holotype), but the syntypes do not have a boss. This feature seems to be a pronounced, large proloculus (possibly with a slightly thickened wall) rather than a true boss of solid calcite.

The specimens identified at ODP Site 1051 do not exactly agree in all features with the holotype, but are more similar to the smaller syntypes in the slide, with a somewhat more convex ventral side, somewhat wider chambers, less raised sutures and a less pronounced umbilicus on the dorsal side, although the proloculus is pronounced (Fig 6.18–6.20). Considering these characteristics, we suggest that this syntype is a juvenile form of *H*. *mantaensis*, and we consider the specimens from Site 1051 also as juvenile forms.

### 3.2 Early Eocene benthic foraminiferal assemblages

#### 3.2.1 The lower Ypresian

**Fig 7:**
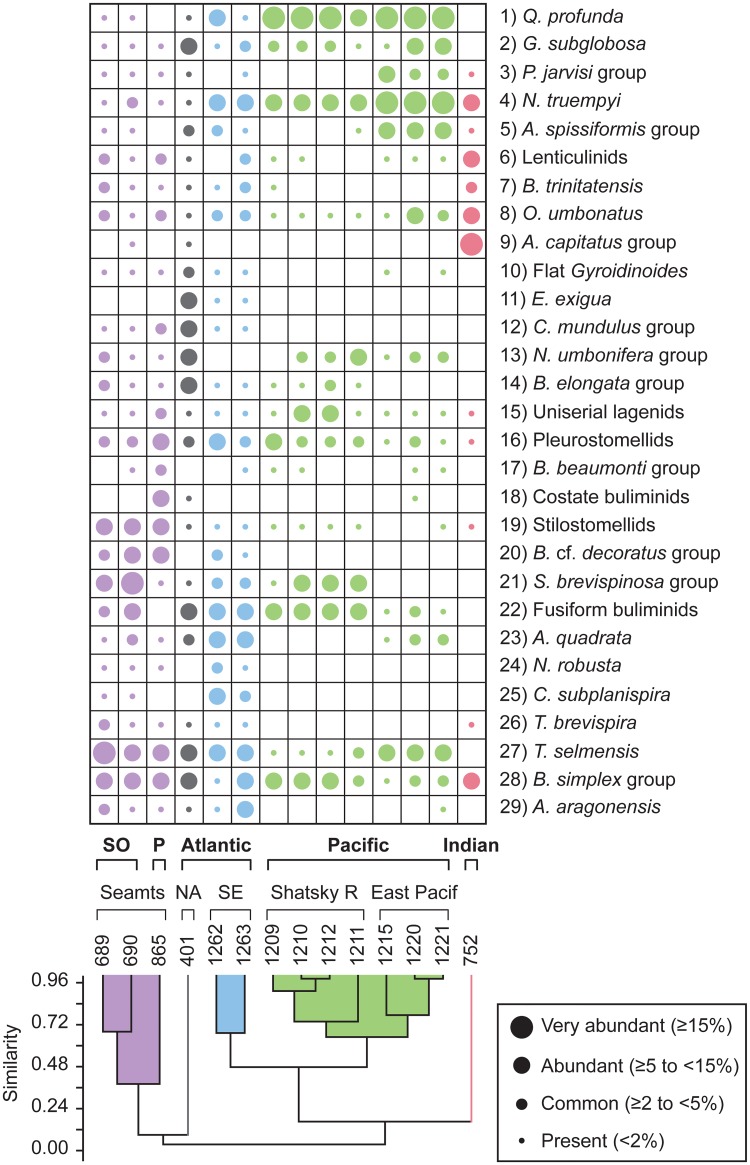
Q-mode dendrogram for ODP/DSDP sites based on relative abundance of selected lower Ypresian species. Cophenetic coefficient = 0.9019. Abbreviations: SO = Southern Ocean; P = Pacific; Seamts = seamounts; NA = North Atlantic; SE = South East; Shatksy R = Shatsky Rise; East Pacif = East Pacific Rise.

Data on the lower Ypresian include 29 species or species groups with a relative abundance ≥2%, of which 24 (>82%) are abundant or very abundant, and only 5 (~17%) show a common relative abundance. These 29 taxa are represented in 14 ocean drilling sites from the Pacific, Atlantic and Indian Oceans (Figs 1 and 7). The species *N*. *truempyi* and *Oridorsalis umbonatus*, and the *B*. *simplex* group, pleurostomellids and uniserial lagenids are ubiquitous (i.e., taxa with a global distribution), whereas *Bulimina trinitatensis* and the *A*. *spissiformis* group, the *P*. *jarvisi* group, lenticulinids and stilostomellids are present in all regions, although not at all sites (Table 4).

Three clusters (based on faunal content) are recognised in two separate branches in the dendrogram (Fig 7). One cluster (green in Fig 7) groups together all Pacific sites (except for Site 865), another cluster (blue) includes the South Atlantic sites, and the third cluster (purple) groups sites which have a seamount setting, including Pacific Site 865 and Southern Ocean Sites 689 and 690. Separate branches hold Indian Site 752 (red) and North Atlantic Site 401 (grey). The groups of sites and individual branches are characterised by the abundance of species or groups of taxa (Fig 7). For instance, taxa 1–5 (Fig 7) seem to mainly represent Pacific assemblages, taxa 6–9 (Fig 7) are more common at Indian Site 752, whereas taxa 10–14 (Fig 7) characterise the assemblage at North Atlantic Site 401. The seamount assemblages are mostly represented by taxa 16–29 (Fig 7), somewhat similar to the SE Atlantic assemblages where these taxa are present but generally less abundant. The difference between those assemblages lies in the higher abundance of *A*. *quadrata*, *Nonionella robusta*, *C*. *subplanispira* or *A*. *aragonensis* at the SE Atlantic sites (taxa 23–25, and 29; Fig 7). In addition, the species *N*. *truempyi* (taxon 4) and *Q*. *profunda* (taxon 1) are abundant at the SE Atlantic sites (especially deepest Site 1262), but common or present at the seamount sites.

**Fig 8:**
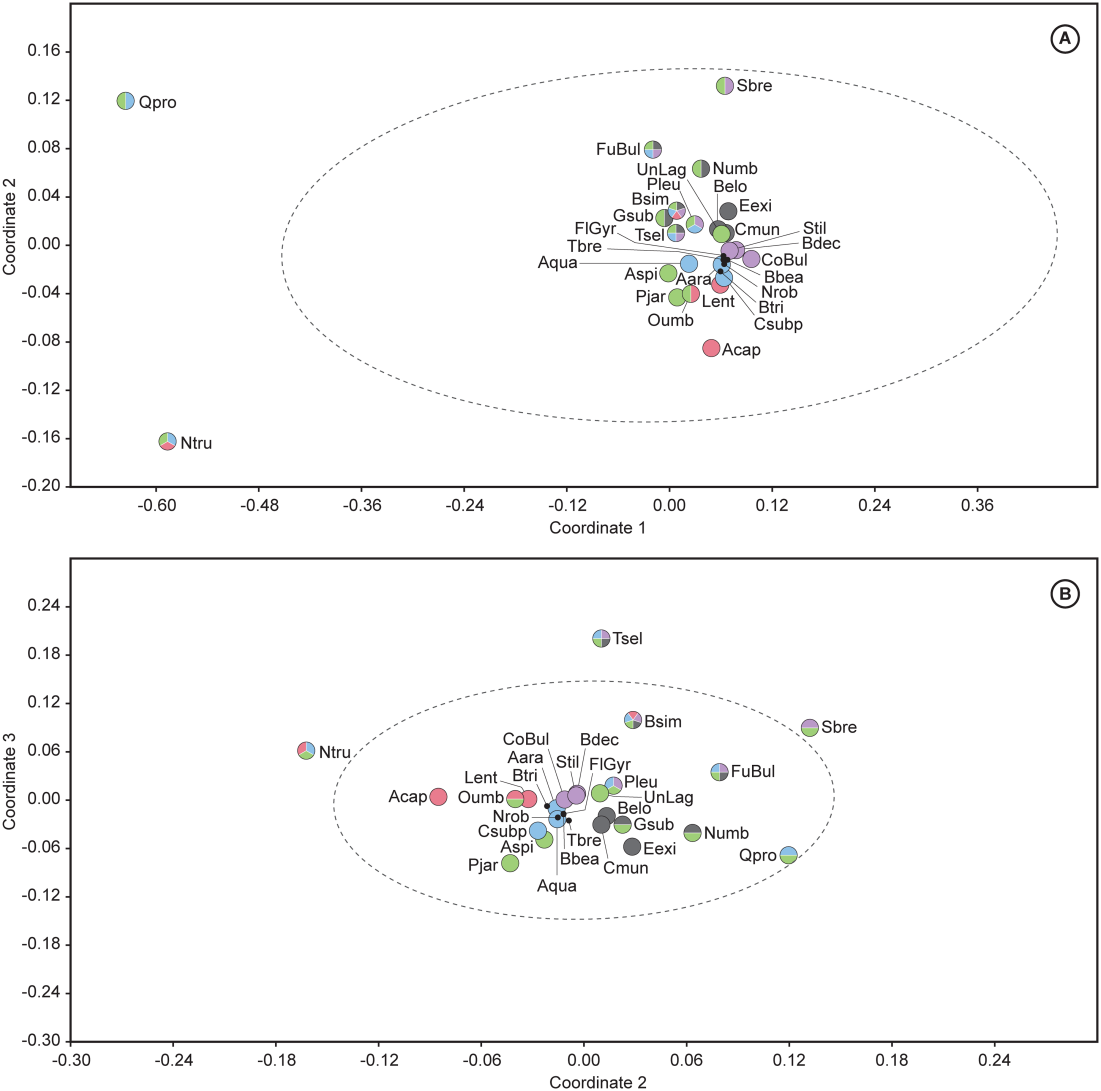
Non-metric multidimensional scaling plots (stress = 0.1158) performed using lower Ypresian species. The difference among the lower Ypresian species based on their relative abundance is shown according to coordinates 1–2 (A) and coordinates 2–3 (B). The abundance of the species located outside the 95% confidence ellipse is significantly distinct (higher) in comparison with the species inside the ellipse. Common species are marked with black dots, and abundant/very abundant species are indicated with colored circles. The color of the circle represents the sites where such species are abundant. Color reference: green-Pacific sites, light blue-SE Atlantic sites, red-Indian site, grey-NA site, and purple-seamount sites. Abbreviations found in Table 4.

The NMDS plots (coordinates 1–2 in Fig 8A, and coordinates 2–3 in Fig 8B) show the species distributed according to their abundance rank, thus species closer to each other indicate similar abundances. The taxa *Q*. *profunda* and *N*. *truempyi* are markedly more abundant than all other species included in the 95% confidence ellipse (Fig 8A). *Quadrimorphina profunda* is very abundant in almost all Pacific sites and abundant in the deepest SE Atlantic site (Site 1262). *Nuttallides truempyi* is very abundant in the East Pacific Rise sites, and abundant at Shatsky Rise (Pacific), SE Atlantic and Indian sites. The species distribution across coordinates 2 and 3 (Fig 8B) shows that *Tappanina selmensis* and *Siphogenerinoides brevispinosa* are highly significant in the lower Ypresian. *Tappanina selmensis* is very abundant at Southern Ocean Site 689, and abundant at the other seamount sites, Atlantic sites (NA and SE) and at the East Pacific Rise sites. On the other hand, *S*. *brevispinosa* is very abundant at Site 690 (Southern Ocean) and abundant at the other Southern Ocean site, as well as at most Shatsky Rise sites (except for the shallowest Site 1209). Inside the confidence ellipse the distribution of the species is mostly associated to the geographic regions where they are abundant, at least considering those species abundant in one region only (i.e., marked with one color only in Fig 8).

#### 3.2.2 The middle Ypresian

The middle Ypresian, for which we have data for fewer sites than for the lower Ypresian and no data for the East Pacific and Shatsky Rise sites, still shows a higher overall species richness (number of species with abundance ≥2%) than the lower Ypresian, with 42 species or taxa groups. The proportion between abundant (or very abundant) and common species is more balanced than in the lower Ypresian, with 25 taxa (>59%) abundant or very abundant, and 17 (~40%) common. The species *N*. *truempyi*, *O*. *umbonatus* and *B*. *trinitatensis*, and those included in the *B*. *simplex* group, *G*. *subglobosa* group, lenticulinids, pleurostomellids, polymorphinids, stilostomellids and uniserial lagenids are ubiquitous, whereas *A*. *aragonensis*, the *A*. *spissiformis* group and the *C*. *eocaenus* group are present in all regions, but not at all sites (Table 5).

**Fig 9:**
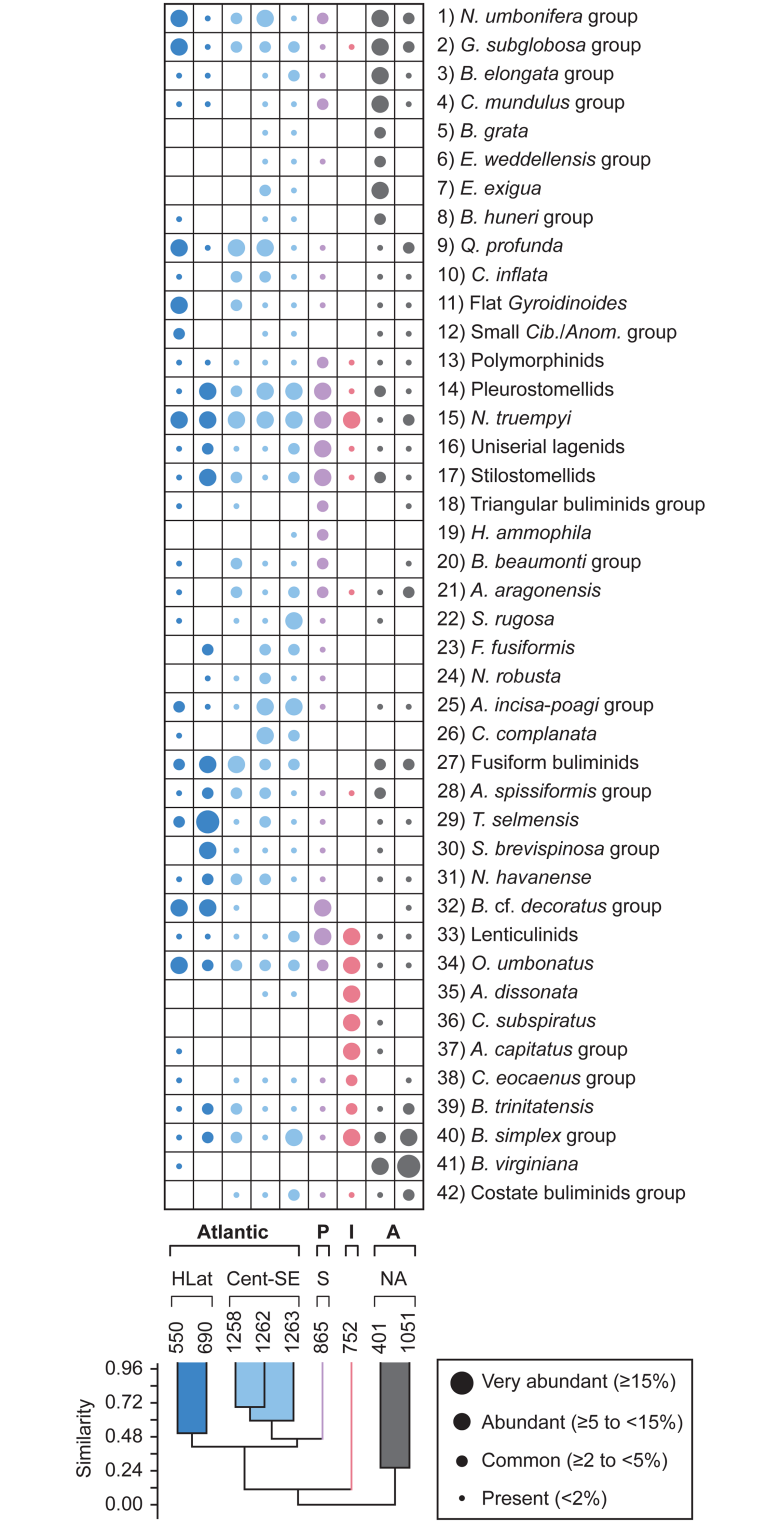
Q-mode dendrogram for ODP/DSDP sites based on relative abundance of selected middle Ypresian species. Cophenetic coefficient = 0.9306. Abbreviations: P = Pacific; I = Indian; A = Atlantic; HLat = High latitude; Cent-SE = Central-South East; S = seamounts; NA = North Atlantic.

The dendrogram shows three clusters and two separate branches (Fig 9). One branch corresponds to Pacific seamount Site 865 (purple in Fig 9), and one to Indian Ocean Site 752 (red), whereas all three clusters contain Atlantic sites. Sites 401 and 1051 form the North Atlantic cluster (grey), which is markedly distinctive. North Atlantic Site 550 (also in the N Atlantic, though at a greater paleodepth) is more similar to Southern Ocean Site 690, and falls in the cluster ‘high latitude’ (dark blue), which resembles the Central-SE Atlantic cluster (light blue) and Site 865 (purple) in faunal content.

Taxa 1–8 as well as taxa 39–42 (Fig 9) mostly define the North Atlantic assemblages, being the first group of taxa more characteristic at Site 401 and the second group more distinctive at Site 1051. Taxa 33–38 (Fig 9) represent the Indian Ocean assemblages, whereas taxa 16–21 (Fig 9) are typical of the seamount Pacific assemblages. The high latitude Atlantic cluster is characterised by taxa 27–32, as well as by taxa 11 and 12 (Fig 9). The Central-SE Atlantic sites are distinguished by taxa 22–26 (Fig 9) as well as by taxon 10, whereas other species such as *Q*. *profunda*, pleurostomellids and *N*. *truempyi* (taxa 9 and 14–15; Fig 9) seem to be equally representative of various regions.

**Fig 10:**
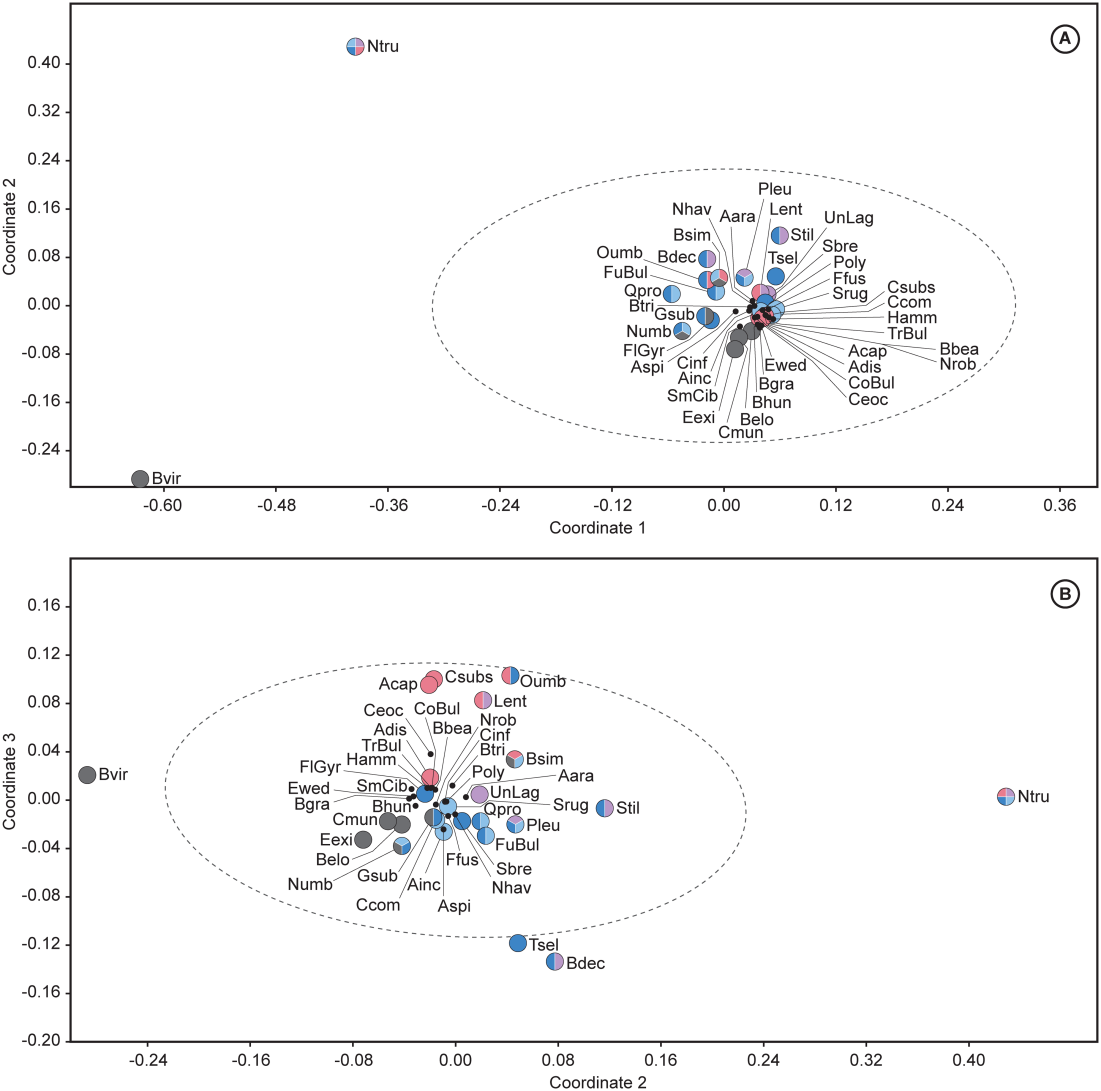
Non-metric multidimensional scaling plots (stress = 0.132) performed using middle Ypresian species. The difference among the middle Ypresian species based on their relative abundance is shown according to coordinates 1–2 (A) and coordinates 2–3 (B). The abundance of the species located outside the 95% confidence ellipse is significantly distinct (higher) in comparison with the species inside the ellipse. Common species are marked with black dots, and abundant/very abundant species are indicated with colored circles. The color of the circle represents the sites where such species are abundant. Color reference: light blue-SE Atlantic sites, dark blue-high latitude Atlantic sites, red-Indian site, grey-NA sites, and purple-seamount site. Abbreviations found in Table 5.

*Nuttallides truempyi* and *B*. *virginiana* are the most significant species across the middle Ypresian, based on their higher abundance ranks compared to all other species located within the 95% confidence ellipse, which includes taxa with similar abundances (Fig 10A). *Nuttallides truempyi* is abundant at all studied sites except for North Atlantic Sites 1051 and 401, whereas *B*. *virginiana* is typical of these two sites, being very abundant at the shallower Site 1051 and abundant at Site 401. *Tappanina selmensis* and the *B*. cf. *decoratus* group also may be important species in the middle Ypresian interval (Fig 10B). The first species is very abundant at Site 690 (Southern Ocean, higher latitude cluster), and the latter is abundant at both Atlantic high latitude sites (550 and 690) and at the Pacific seamount Site 865. Abundant species from North Atlantic, Indian and Central-SE regions are easily recognised as groups (considering those species abundant in one region only, i.e., marked with one color in Fig 10), whereas abundant species from high latitude sites (550 and 690) are more spread out inside the confidence ellipse.

## 4. Discussion

### 4.1 The lower Ypresian fauna

The lower Ypresian assemblages have been described as typical post-extinction faunas (e.g., [31, 36, 37, 139]), including fewer species (in this case, species with a relative abundance ≥2%), and higher dominance of abundant species (>5%). This is as expected, because this interval is directly above the Paleocene-Eocene boundary (covering up to ∽0.8 m.y. after the P-E boundary), thus includes the peak PETM environmental disturbance directly postdating the severe extinction of deep-sea benthic foraminifera. The environmental disturbance includes increased temperature, low oxygen levels at some locations, and ocean acidification. Its effects on benthic foraminiferal fauna persisted for at least 260 kyr after the extinction, as suggested by low diversity assemblages at Maud Rise [30]. The decreased diversity probably was the combined result of the global extinction and local or temporary disappearance of species (e.g., [8]). The concomitant dominance of abundant species likely reflects the proliferation of opportunistic species in perturbed environments (e.g., [168]).

The relative abundance of the taxa in the lower Ypresian assemblages (data compilation from 14 sites; Fig 7) appears to be linked mainly to geographic regions (Pacific, Atlantic and Indian Oceans), and to a lesser extent to specific environmental settings (seamounts). Non-seamount Pacific sites were located in truly pelagic, open ocean settings far away from coastal influence (Fig 1), and contain more abundant taxa that are generally considered oligotrophic (such as *Q*. *profunda* and *N*. *truempyi*; e.g., [31]) than the Walvis Ridge Atlantic sites, with the latter closer to continental margins. Proximity to continental margins may be related to a higher amount of food reaching the seafloor, due to coastal upwelling along continental margins and higher input of nutrients by rivers (e.g., [169]), and also due to lateral transport of refractory organic matter (e.g., [170]).

The fact that the ‘seamount cluster’ includes sites at great distance from each other (Atlantic and Pacific Oceans) suggests that these assemblages may have been controlled by parameters specific to the seamount setting (e.g., active current patterns), independent of their geographic location. Such assemblages are strongly dominated by cylindrical infaunal taxa (such as stilostomellids), which seem to prefer relatively high organic carbon flux regularly delivered to the seafloor in non-seamount settings [171]. However, at seamount ecosystems, these taxa may have developed a suspension feeding strategy as they adapted to a specific food supply brought in or concentrated by current systems around the seamounts (e.g., [43]). Such currents are enhanced around the steep topography of the seamounts, causing removal of fine-grained food particles from benthic communities in some places, and concentration in other parts by trophic focusing (e.g., [172, 173]).

North Atlantic Site 401 (paleodepth ~1900 m) does not cluster with other Atlantic sites, and has an about equal abundance of infaunal and epifaunal taxa (Table 4), in contrast to most other sites. This site is characterised by a high abundance of epifaunal taxa (in comparison to other sites) such as *Epistominella exigua* (indicative of a pulsed food supply [107, 174, 175]), the *C*. *mundulus* group, and the *N*. *umbonifera* group, which are less abundant at other sites. This assemblage might reflect a more variable food supply at these fairly high northern latitudes, possibly with high seasonality, or higher levels of oxygenation than at other sites (see below, 4.3). For this time interval, we have no data from other North Atlantic sites to test hypotheses about the parameters controlling the benthic assemblages.

*Quadrimorphina profunda*, *N*. *truempyi*, *T*. *selmensis* and *S*. *brevispinosa* are the most notable species, as inferred from the NMDS plots (Fig 8). *Nuttallides truempyi* and the deep-water species *Q*. *profunda* may indicate lower food supply (e.g., [15, 31, 36]). Inside the 95% confidence ellipse, abundant species characterising the Pacific assemblages are located relatively closer to the oligotrophic species *N*. *truempyi* and *Q*. *profunda* than the rest of species typical from other regions (Fig 8A), which agrees with the fact that Pacific sites are located in open ocean settings (far from continental margins), with lower food supply to the seafloor. Moreover, *N*. *truempyi* may also reflect a potential resilience to surviving in carbonate-corrosive waters [31, 38, 39].

On the other hand, *T*. *selmensis* and *S*. *brevispinosa* may have behaved as opportunistic taxa, blooming in response to environmental perturbations associated to the PETM. Both species tolerate stressed/disturbed environments (e.g., [38, 39, 139, 176]) and possibly indicate a high food supply (e.g. [177]), although this is debated for *T*. *selmensis* (see e.g., [31, 178]). Other buliminids such as the *B*. *simplex* group and the fusiform buliminid group are located closer to *T*. *selmensis* and *S*. *brevispinosa* in the NMDS plot, but inside the confidence ellipse (Fig 8B). Sprong et al. [137] suggested that *B*. *kugleri* (included in the fusiform buliminid group) may have been an opportunistic species, reported from a wide paleodepth range and dependent on food supply, and commonly abundant just after the Paleocene-Eocene extinction [14, 139]. Additionally, Takeda and Kaiho [36] suggested that *Q*. *profunda* is an opportunistic species that may have become more tolerant to stressed conditions after the PETM, thus accounting for its location relatively close to the other opportunistic species (Fig 8B). Following this line of reasoning, we suggest that the *N*. *umbonifera* group may also have had an opportunistic behavior, considering that *N*. *umbonifera* and *Osangularia* sp. 1 (both included in the *N*. *umbonifera* group) peaked in abundance together with other opportunistic taxa across the H2 event at DSDP Site 550 [63].

### 4.2 The middle Ypresian fauna

Assemblages in the middle Ypresian interval have a higher diversity (i.e., higher number of species) than those from the lower Ypresian, and higher equitability, i.e., a similar number of abundant and common taxa despite the smaller number of studied sites (9), and the occurrence of several lesser hyperthermal events (see e.g., [179]). The higher diversity of these assemblages may reflect long-term recovery after the PETM extinction through evolution, migration, and return of ‘Lazarus’ taxa (e.g., *Siphonodosaria subspinosa*, *Strictocostella pseudoscripta* [8]). This sustained recovery suggests that the effects of the Eocene hyperthermals on the benthic fauna on a global scale were considerably less than the effect of the PETM, despite local short-term, reversible declines in diversity and changes in assemblage composition during the early Eocene hyperthermals [43, 60, 62, 63].

As in the lower Ypresian, the sites generally cluster largely by geographical location rather than bathymetry, with a North Atlantic and an Atlantic cluster, and separate branches for Indian and Pacific Oceans. The exception appears to be the clustering of North Atlantic Site 550 with Southern Ocean Site 690 (high latitude cluster), whereas Sites 550 and 401 are clustering far apart, though they are in relatively close geographic proximity (with Site 550 at greater depth; Figs 1 and 3). Sites 401 and 550 share high abundances of the *N*. *umbonifera* group and the *G*. *subglobosa* group, but Site 550 does not have abundant *B*. *virginiana*, and neither other species such as *E*. *exigua*, the *B*. *elongata* group or the *C*. *mundulus* group, probably due to its greater depth (see below). Despite their seamount setting location, Site 690 is separated from Site 865 in the middle Ypresian (in contrast to the lower Ypresian interval) (Fig 9). Site 690 is characterised by higher abundances of the opportunistic *T*. *selmensis* and the *S*. *brevispinosa* group than Site 865 (although these sites share high abundances of stilostomellids), possibly indicating that Site 690 and Site 550 (which with it clusters) were more susceptible to environmental perturbations during hyperthermal events, with more pronounced effects at great depths (Site 550) and higher latitudes (Site 690). This relation between fluctuations in abundance of opportunistic taxa and hyperthermals is inferred, for example, from major assemblage changes documented during the ETM2 and H2 events (including variations in relative abundances and decreased number of specimens) at Site 550 [63]. Furthermore, the high susceptibility of these two sites may be related to the abyssal, oligotrophic setting at Site 550, which makes it very sensitive to trophic changes, and by the seamount setting at Site 690, making the site sensitive to hydrographic changes probably enhanced by its higher latitude location.

The middle Ypresian assemblage in the Indian Ocean differs from the other assemblages, as in the lower Ypresian, possibly because the site is much shallower (~1000 m paleodepth), with a higher food supply and more stable seawater carbonate saturation.

According to the NMDS plot (Fig 10), the most distinguished species during the middle Ypresian interval are *B*. *virginiana*, *N*. *truempyi*, *T*. *selmensis* and the *B*. cf. *decoratus* group, with the first species showing palaeogeographic and paleobathymetric restrictions. *Bulimina virginiana* has been documented from shallower depths along the Northeast coast of the United States (e.g., [176, 180, 181]), and its abundant occurrence at North Atlantic Sites 1051 (~1500 m paleodepth) and 401 (~1900 m paleodepth) may indicate that it migrated from shallower depths after the PETM extinction. At NW Atlantic Site 550 (~3900 m paleodepth), *B*. *virginiana* is present but not abundant, suggesting that it may have not been able to thrive at sites deeper than ~2000 m. Furthermore, *B*. *virginiana* was not recognised at any of our other study sites, suggesting that it may have been restricted geographically, occurring in the North Atlantic only.

Abundant *B*. *virginiana* might indicate higher food supply at North Atlantic Sites 401 and 1051, considering that it is a buliminid taxon that lived at shallower, more eutrophic depths. This interpretation is supported by the opposite location of *N*. *truempyi* and *B*. *virginiana* in the NMDS plot (Fig 10), which may indicate distinct ecological preferences with *N*. *truempyi* reflecting oligotrophic conditions [31, 182]. This hypothesis is supported by the location of uniserial taxa relatively closer to *N*. *truempyi* than to other species (Fig 10B), because stilostomellids may indicate overall oligotrophic conditions but fairly active current systems (as at Site 865), leading to trophic focusing and decoupling between productivity and arrival of food to the seafloor [43]. *Nuttallides truempyi* may also indicate carbonate corrosive waters (e.g., [31]), which could be supported by the location of lenticulinids, a group that is resistant to dissolution [183], and other infaunal taxa such as *O*. *umbonatus*, the *B*. *simplex* group, uniserial lagenids, pleurostomellids, *Q*. *profunda*, or the fusiform buliminid group, somewhat close to *N*. *truempyi*. However, we did not find evidence for CaCO_3_-corrosiveness at the studied sites during the middle Ypresian interval (see section 4.3).

The *B*. cf. *decoratus* group might be interpreted as reflecting an abundant food supply, since it is a buliminid taxon (*sensu lato*), but its abundance in assemblages with abundant oligotrophic taxa at Site 550 [63] suggests that this group is tolerant to meso-oligotrophic conditions. Likewise, Boscolo Galazzo et al. [184, 185] argued that the species *B*. *crenulata* and *B*. *huneri* (which resemble *B*. *decoratus*) might indicate a lower food flux than other buliminids. The proximity with the opportunistic *T*. *selmensis* [178] in the NMDS plot (Fig 10B) suggests that the *B*. cf. *decoratus* group might have had an opportunistic behavior. These taxa, like *T*. *selmensis*, are biserial with an ornamented wall.

### 4.3 Common features in the Ypresian time intervals

A common feature of benthic foraminiferal assemblages across both Ypresian intervals is the marked and widespread dominance of infaunal taxa [14, 29], including taxa placed in the buliminid group *sensu lato* (triserial and biserial forms), in the uniserial groups (stilostomellids, unserial lagenids), or the pleurostomellids. The dominance of infaunal groups, which has been linked to some combination of high food—low oxygen conditions (TROX model, [100, 101]) in the present oceans, is not easy to explain during the warm early Eocene. In the modern oceans, food supply to the seafloor is generally linked to high primary productivity in surface waters. However, the transfer of food from the surface to the seafloor is not reflected by a simple logarithmic function, as traditionally thought (e.g., [186, 187]), but it varies strongly by location and ecosystem structure (e.g., [188, 189]). Over longer time periods and across climate change events, one might expect that the link between surface primary productivity and food supply to the benthos (bentho-pelagic coupling) has not necessarily always been strong and may have been broken, for instance through processes of trophic focusing on seamounts [43], carbonate dissolution in bottom waters [88], or changes in the amount of remineralization of organic matter, with higher rates expected at higher temperatures (e.g., [108, 109, 185]).

Despite these complexities, food supply to the deep sea overall is expected to have been lower in warmer than in cooler periods of Earth history (e.g., [15]), due to a combination of factors including increased stratification in warmer oceans, which limits upwelling of nutrient-enriched deeper waters, thus primary productivity (e.g., [95]), and the increased remineralization of primary-produced material due to higher metabolic rates or remineralizers, which limits export productivity (e.g., [108, 109, 185]). This hypothesis is supported by the observation that delivery of food to the seafloor may have declined strongly during hyperthermal events such as ETM2 [60, 62].

The high abundance of infaunal taxa in the warm Paleogene oceans has been linked to lower oxygen conditions rather than to a high food supply [190, 191], due to the lower solubility of oxygen at higher temperatures. However, moderately-low oxygen conditions (rather than anoxia) do not appear to strongly affect living deep-sea benthic foraminifera (e.g., [101, 107]. In fact, some foraminifera have been observed to be able to calcify under anoxic conditions [192]. The lower oxygen hypothesis is, however, supported by geochemical evidence that the deep sea may have been less oxygenated in the early Paleogene in general [193–195]. Oxygen minimum zones in the Paleogene may have been more extensive, thus covering more of the seafloor, with more pronounced deoxygenation during hyperthermal events [196–199], at least at Atlantic sites [197]. Despite the observation that low oxygen conditions do not seem to affect modern living benthic foraminifera in overall well oxygenated cool oceans, the overall, long-term lowered oxygen conditions of the Paleogene may have affected the composition of benthic assemblages. This may not have been a direct effect, but could have operated through giving a competitive advantage to infaunal taxa using nitrate as electron acceptor through denitrification, as seen in many extant species, especially deep-infaunal taxa (e.g., [200–203]. This denitrification process could have been more pronounced at high nitrate levels in the deep waters, e.g. as produced by high rates of remineralization.

In addition, the occurrence of multiple short-lived hyperthermal events during the early Eocene (e.g., [179]), associated with widespread carbonate dissolution in the deep sea, may have resulted in pore waters being more carbonate-saturated than bottom waters, at least in some areas in the Atlantic Ocean [88]. These conditions would give competitive advantage to calcareous infaunal taxa that lived and calcified buried in the sediment, in contact with the more saturated pore waters rather than bottom waters. However, we do not see evidence for the long-term occurrence of widespread, highly corrosive bottom waters at the depths of our sites, because non-calcareous agglutinated benthic foraminifera are not common in any of the studied samples (lower and middle Ypresian), or in the South Atlantic samples studied by Müller-Merz and Oberhänsli [24], and because the carbonate content is generally high at the studied sites (except during the hyperthermals at some locations, e.g., [37, 82]). In addition, reconstructions of the position of the Calcium Carbonate Compensation Depth (CCD) show that it was shallower than today, but not by more than several hundred meters, leaving most of our sites above the CCD (with the exception of the PETM and possibly ETM2) [204].

Another common feature in the assemblages across both time intervals is the fact that neither the species (and/or groups of species) nor the number of taxa with a global distribution (i.e., ubiquitous species) differ significantly: only 4 taxa that were not ubiquitous in the lower Ypresian became ubiquitous in the middle Ypresian, and 1 group from the lower Ypresian was no longer widely distributed in the upper interval. We suggest that the palaeogeographic distribution of benthic assemblages did not change significantly within the early Eocene (lower and middle Ypresian), although we have data on fewer sites for the middle Ypresian interval (specifically lacking data on the equatorial Pacific and Shatsky Rise, with the exception of seamount Site 865). Our present dataset indicates that the relative abundance of benthic foraminifera in the Ypresian was linked mainly to geographic regions (Pacific, Atlantic and Indian Oceans), and to a lesser extent to specific environmental settings (seamounts). Sites on depth transects (East Pacific Rise, Shatsky Rise, Walvis Ridge) generally group closely together, whereas sites at similar depths in different regions (e.g., Sites 401-North Atlantic, 1209-Pacific and 690-Southern Ocean, all at ~1800–2000 m) do not cluster together (Fig 7). This observation suggests that paleobathymetry (within the studied range) was a less important determining factor than geography for this time interval, which is in contrast with the cosmopolitan Velasco fauna. Note that we are not certain why the one assemblage from the Indian Ocean is clearly distinct from the Atlantic and Pacific clusters, as it differs in both geography and a shallower paleodepth (1000 m) (Fig 3). We argue that the overall similarity between assemblages along a depth transect, studied by the same set of authors (Shatsky Rise: Takeda and Kaiho [36]; equatorial Pacific: Nomura and Takata [37]; Walvis Ridge: Thomas and Shackleton [34], Jennions et al. [62]; Maud Rise: Thomas [14]) is probably not due to different approaches to taxonomy. These geographic differences persist in our taxonomic compilation (morphological groups rather than specific taxa), which aimed at correcting for such effects and making this global compilation possible.

Our assessment of benthic foraminifera across the two Ypresian intervals provides new insights into the main structure and composition of early Eocene global assemblages. Most published compilations have mainly focused on the major extinction across the Paleocene-Eocene boundary (e.g., [11, 15, 26, 27, 31]), documenting the transition from the long-lived Cretaceous-Paleocene (Velasco) fauna, which showed transient assemblage changes across the Cretaceous/Paleogene impact event (e.g., Alegret et al. [205], and references therein), to the Paleogene fauna, which persisted from the PETM to the Eocene/Oligocene turnover. According to those studies, the Eocene post-extinction fauna was characterised by low-diversity and high dominance of some species like *N*. *truempyi*, abyssaminids and/or buliminids (e.g., [31, 36, 139]). Our analysis of the lower Ypresian interval confirms this basic pattern, i.e., we confirm that the lower Ypresian globally contains fewer common species, and recognise *N*. *truempyi* and *Q*. *profunda* (usually included in the abyssaminid group) as some of the most important species. We also confirm the abundance of buliminid taxa (Figs 7 and 8). In addition, we note that both Ypresian assemblages contain abundant pleurostomellids and stilostomellids (at least in some regions), which are quantitatively rare and unimportant within the preceding Velasco fauna [160]. In contrast to our Ypresian records, these authors noted that several agglutinated taxa are typical of the Velasco fauna. On the other hand, taxa such as *N*. *truempyi* and buliminids remain equally abundant in both faunas (Velasco and Ypresian). Furthermore, the abyssal Barbados fauna (2500–3000 m paleodepth) from the middle Eocene-Oligocene contrasts to both Ypresian assemblages by its decreasing abundance of *N*. *truempyi* (which disappeared at the end of the Eocene together with *Abyssamina* species), lower abundance of buliminids, and dominance of *G*. *subglobosa* [206]. Therefore, a sucession of faunas such as Velasco-Ypresian (also called Walvis Ridge fauna, see next section)-Barbados may cover the transition of deep-sea benthic foraminiferal assemblages from the Cretaceous through the Oligocene.

### 4.4 Walvis Ridge fauna

Benthic foraminiferal assemblages from Walvis Ridge across the lower and middle Ypresian intervals may be considered as representative deep-sea fauna (Figs 7 and 9), recovering from a major perturbation (PETM) during a warm period with superimposed hyperthermal events (early Eocene). Therefore, we propose the name ‘Walvis Ridge fauna’ for future reference to the early Eocene (Ypresian) deep-sea benthic foraminiferal assemblages.

Specifically, the most common taxa that characterise the Walvis Ridge fauna include *N*. *truempyi*, *Q*. *profunda*, *T*. *selmensis*, *A*. *aragonensis*, *O*. *umbonatus*, *N*. *robusta*, pleurostomellids, lenticulinids, and those species comprised into the informal taxonomic groups called fusiform buliminids, *B*. *simplex*, *G*. *subglobosa*, and *A*. *spissiformis*. Other common taxa included in this fauna are *C*. *subplanispira*, *C*. *complanata*, *C*. *inflata*, *A*. *quadrata*, *E*. *exigua*, *S*. *rugosa*, *B*. *trinitatensis*, uniserial lagenids, stilostomellids, and the species included in some informal groups such as *N*. *umbonifera*, *N*. *havanense*, *A*. *incisa*-*poagi*, *B*. cf. *decoratus*, *B*. *elongata*, costate buliminds, *F*. *fusiformis*, and *S*. *brevispinosa*. Species such as *T*. *brevispira*, *B*. *grata*, *H*. *ammophila*, *A*. *dissonata*, polymorphinids, and those included in the informal groups called *P*. *jarvisi*, flat *Gyroidinoides*, *C*. *eocaenus*, *C*. *mundulus*, small *Cibicidoides*/*Anomalinoides*, *E*. *weddellensis*, *B*. *beaumonti*, and *B*. *huneri* are considered as accessory taxa in the Walvis Ridge fauna.

Not all species included into this fauna shown a global distribution at lower bathyal-abyssal paleodepths (1500–3500 m), but similar assemblages can be recognised in other Atlantic regions and in the equatorial Pacific during the lower-middle Ypresian (∽56–50.7 Ma [25]).

## 5. Conclusions

Our study synthesizes and evaluates, for the first time, early Eocene (Ypresian) benthic foraminiferal assemblages living in a greenhouse world with superimposed, short-lived warming events, after a significant extinction. Thus, this taxonomic and paleoecologic compilation constitutes a tool for reconciling different taxonomic concepts and provides an analysis of benthic foraminiferal diversity variability in space and time, and recovery and diversification from PETM extinction.

Variations in early Eocene benthic foraminiferal assemblages at the studied sites in the Pacific, Atlantic and Indian Oceans are mainly correlated to differences in palaeogeography (proximity to continental margins, seamount settings, or open-ocean pelagic settings), and to a lesser degree to paleobathymetry.

The lower Ypresian assemblages are characterised by lower diversity and equitability, i.e., high dominance of abundant species (or groups of taxa) that reflect a perturbed fauna, which resulted from the global extinction associated to the PETM. The most abundant species in this interval may have had an opportunistic behavior and were tolerant to disturbed environments. In the middle Ypresian interval, benthic foraminiferal assemblages reflect a recovering fauna, as expressed in higher diversity and increasing numbers of common taxa, and less affected by environmental perturbations despite the repeated occurrence of hyperthermal events of lesser magnitude than the PETM. The increased diversity and equitability resulted from migration from shallower waters, return of Lazarus taxa from refugia, and evolution of new taxa. We propose the name ‘Walvis Ridge fauna’ for future reference to the early Eocene (Ypresian) deep-sea benthic foraminiferal assemblages, which lived in a greenhouse world with superimposed hyperthermal events, during a phase of recovery from the PETM extinction.

## Supporting information

S1 Appendix Taxonomic list. Most common benthic foraminifera during the early Eocene and other species mentioned in the text.
(DOCX)

S1 Fig Number of species vs. number of specimens in samples from ODP Sites 1051 and 1258. (TIF)

S1 Table Quantitative data of benthic foraminifera in the studied samples from ODP Site 1051 and 1258. (XLSX)

S2 Table Quantitative data of benthic foraminifera in the studied samples from ODP Site 1262 and 1263. (XLSX)

S3 Table Quantitative data of benthic foraminifera in the studied samples from DSDP site 401. (XLSX)

S4 Table Benthic foraminifera across the lower Ypresian interval. (XLSX)

S5 Table Benthic foraminifera across the middle Ypresian interval. (XLSX)

S6 Table Number of samples, specimens and species, and size fraction studied in each site. (XLSX)

## References

[1] Snider LJ, Burnet BR, Hessler RR. The composition and distribution of meiofauna and nanobiota in a central North Pacific deep-sea area. Deep-Sea Research Part I 1984; 31: 1225–1249.

[2] Gooday AJ, Levin LA, Linke P, Heeger T. The role of benthic foraminifera in deep-sea food webs and carbon cycling In: Rowe GT, Pariente V, editors. Deep-Sea Food Chains and the Global Carbon Cycle. Dordrecht: Kluwer Academic Publishers; 1992 pp. 63–91.

[3] Gooday AJ, Bett BJ, Shires R, Lambshead PJD. Deep-sea benthic foraminiferal species diversity in the NE Atlantic and NW Arabian Sea: a synthesis. Deep-Sea Research Part II 1998; 45: 165–201.

[4] Verity PG, Smetacek V, Smayda T. Status, trends and the future of the marine pelagic ecosystem. Environmental Conservation 2002; 29: 207–237. 10.1017/S0376892902000139

[5] Katz ME, Wright JD, Katz DR, Miller KG, Pak DK, Shackleton NJ, et al Early Cenozoic benthic foraminiferal isotopes: species reliability and interspecies correction factors. Paleoceanography 2003; 18: 1024 10.1029/2002PA000798

[6] Katz ME, Cramer BS, Franzese A, Hönisch B, Miller KG, Rosenthal Y, et al Traditional and emerging geochemical proxies in Foraminifera. Journal of Foraminiferal Research 2010; 40: 165–192.

[7] Bolli HM, Beckmann JP, Saunders JB. Benthic foraminiferal biostratigraphy of the south Caribbean region. Cambridge UK: Cambridge University Press; 1994.

[8] Hayward BW, Kawagata S, Sabaa AT, Grenfell HR, Van Kerckhoven L, Johnson K, et al The last global extinction (Mid-Pleistocene) of deep-sea benthic foraminifera (Chrysalogoniidae, Ellipsoidinidae, Glandulonodosariidae, Plectofrondiculariidae, Pleurostomellidae, Stilostomellidae), their Late Cretaceous-Cenozoic history and taxonomy Cushman foundation for foraminiferal research, Special volume 43 Lawrence USA: Allen Press; 2012.

[9] Van Morkhoven FPCM, Berggren WA, Edwards AS. Cenozoic cosmopolitan deep-water benthic foraminifera. France: Bulletin des Centres de Recherche Exploration Production Elf-Aquitaine Memoir 11; 1986.

[10] Alegret L, Thomas E. Upper Cretaceous and lower Paleogene benthic foraminifera from northeastern Mexico. Micropaleontology 2001; 47: 269–316.

[11] Tjalsma RC, Lohmann GP. Paleocene-Eocene bathyal and abyssal benthic foraminifera from the Atlantic Ocean. Micropaleontology Special Publication 1983; 4: 1–89.

[12] Berggren WA, Miller KG. Cenozoic bathyal and abyssal benthic foraminiferal zonations. Micropaleontology 1989; 35: 308–320. 10.2307/1485674

[13] Miller KG, Katz ME, Berggren WA. Cenozoic deep-sea benthic foraminifera: a tale of three turnovers In: Takayanagi Y, Saito T, editors. Studies in benthic foraminifera, BENTHOS 90. Sendai Japan: Tokai University Press; 1992 pp. 67–75.

[14] Thomas E. Late Cretaceous through Neogene deep-sea benthic foraminifers (Maud Rise, Weddell Sea, Antarctica). Proceedings of the Ocean Drilling Program, Scientific Results 1990; 113: 571–594.

[15] Thomas E. Cenozoic mass extinctions in the deep sea; what disturbs the largest habitat on Earth? In: Monechi S, Coccioni R, Rampino M, editors. Large ecosystem perturbations: causes and consequences. USA: Geological Society of America Special Paper 424; 2007 pp. 1–24.

[16] Thomas E, Zachos JC, Bralower TJ. Deep-sea environments on a warm Earth: latest Paleocene-early Eocene In: Huber B, MacLeod K, Wing S, editors. Warm climates in Earth history. Cambridge UK: Cambridge University Press; 2000 pp. 132–160.

[17] Holbourn A, Henderson AS, MacLeod N. Atlas of benthic foraminifera. UK: Wiley-Blackwell Publishing; 2013.

[18] Cushman JA. Some new foraminifera from the Velasco Shale of Mexico. Contributions from the Cushman Laboratory for Foraminiferal Research 1925; 1: 18–23.

[19] Cushman JA. The foraminifera of the Velasco shale of the Tampico embayment. Bulletin of the American Association of Petroleum Geologists 1926; 10: 581–612.

[20] White MP. Some index foraminifera of the Tampico Embayment area of Mexico Part I. Journal of Paleontology 1928; 2: 177–215.

[21] White MP. Some index foraminifera of the Tampico Embayment area of Mexico Part II. Journal of Paleontology 1928; 2: 280–317.

[22] White MP. Some index foraminifera of the Tampico Embayment area of Mexico Part III. Journal of Paleontology 1929; 3: 30–58.

[23] Schnitker D. Cenozoic deep-water benthic foraminifera, Bay of Biscay. In: Montadert L, Roberts DG, et al., editors. Initial reports of the Deep-Sea Drilling Project 48; Washington: U.S. Government Printing Office; 1979. pp. 377–414.

[24] Müller-Merz E, Oberhänsli H. Eocene bathyal and abyssal benthic foraminifera from a South Atlantic transect at 20–30°S. Palaeogeography, Palaeoclimatology, Palaeoecology 1991; 83: 117–171.

[25] Vandenberghe N, Hilgen FJ, Speijer RP. The Paleogene period In: Gradstein FM, Ogg JG, Schmitz MD, Ogg GM, editors. The Geologic Time Scale 2012. Elsevier; 2012 pp. 855–921.

[26] Beckmann JP. Distribution of benthic foraminifera at the Cretaceous-Tertiary boundary of Trinidad (West Indies). In: Rosenkrantz A, Brotzen F, editors. International Geological Congress, Report 21st session. Norden, Copenhagen; 1960. pp. 57–69.

[27] Von Hillebrandt A. Das Paläozän und seine Foraminiferenfauna im Becken von Reichenhall und Salzburg. Munich, Germany: Bayerische Akademie der Wissenschaften, 1962.

[28] Boersma A. Oligocene and other Tertiary benthic foraminifers from a depth traverse down Walvis Ridge, Deep Sea Drilling Project Leg 74, Southeast Atlantic. In: Moore TC Jr, Rabinowitz PD, et al., editors. Initial Reports of the Deep Sea Drilling Project 75, Washington: U.S. Government Printing Office; 1984. pp. 1273–1300.

[29] Thomas E. Development of Cenozoic deep-sea benthic foraminiferal faunas in Antarctic waters In: Crame JA, editor. Origins and evolution of the Antartic biota. London, UK: Geological Society London Special Publication 47; 1989 pp. 283–296. 10.1144/GSL.SP.1989.047.01.21

[30] Thomas E. Late Cretaceous-early Eocene mass extinctions in the deep sea. In: Sharpton VL, Ward PD, editors. Global catastrophes in Earth history; An interdisciplinary conference on impacts, volcanism, and mass mortality. USA: Geological Society of America Special Paper 247; 1990. pp. 481–495.

[31] Thomas E. The biogeography of the late Paleocene benthic foraminiferal extinction In: Aubry MP, Lucas S, Berggren WA, editors. Late Paleocene-early Eocene biotic and climatic events in the marine and terrestrial records. New York, USA: Columbia University Press; 1998 pp. 214–243.

[32] Katz ME, Miller KG. Early Paleogene benthic foraminiferal assemblages and stable isotopes in the Southern Ocean. In: Ciesielski PF, Kristoffersen Y, et al., editors. Proceedings of the Ocean Drilling Program Scientific Results 114. Texas, USA: College Station, ODP; 1991. pp. 481–512.

[33] Coccioni R, Di Leo RC, Galeotti S, Monechi S. Integrated biostratigraphy and benthic foraminiferal faunal turnover across the Paleocene-Eocene boundary at Trabakua Pass section, northern Spain. Palaeopelagos 1994; 4: 87–100.

[34] Thomas E, Shackleton NJ. The Palaeocene-Eocene benthic foraminiferal extinction and stable isotope anomalies In: Knox RWO’B, Corfield RM, Dunay RE, editors. Correlation of the Early Paleogene in Northwest Europe. London, UK: Geological Society London Special Publication 101; 1996 pp. 401–441.

[35] Galeotti S, Kaminski MA, Coccioni R, Speijer RP. High-resolution deep-water agglutinated foraminiferal record across the Paleocene/Eocene transition in the Contessa Road section (central Italy) In: Bubik M, Kaminski MA, editors. Proceedings of the Sixth International Workshop on Agglutinated Foraminifera. Krakow, Poland: Grzybowski Foundation Special Publication 8; 2004 pp. 83–103.

[36] Takeda K, Kaiho K. Faunal turnovers in central Pacific benthic foraminifera during the Paleocene-Eocene thermal maximum. Palaeogeography, Palaeoclimatology, Palaeoecology 2007; 251: 175–197. 10.1016/j.palaeo.2007.02.026.

[37] Nomura R, Takata H. Data report: Paleocene/Eocene benthic foraminifers, ODP Leg 199 Sites 1215, 1220 and 1221, equatorial central Pacific Ocean. In: Wilson PA, Lyle M, et al., editors. Proceedings of the Ocean Drilling Program, Scientific Results 199. Texas, USA: College Station, ODP; 2005. pp. 1–34.

[38] Alegret L, Ortiz S, Molina E. Extinction and recovery of benthic foraminifera across the Paleocene-Eocene Thermal Maximum at the Alamedilla section (Southern Spain). Palaeogeography, Palaeoclimatology, Palaeoecology 2009; 279: 186–200.

[39] Alegret L, Ortiz S, Orue-Etxebarria X, Bernaola G, Baceta JI, Monechi S, et al The Paleocene-Eocene Thermal Maximum: new data from the microfossil turnover at the Zumaia section, Spain. Palaios 2009; 24: 318–328.

[40] Alegret L, Ortiz S, Arenillas I, Molina E. What happens when the ocean is overheated? The foraminiferal response across the Paleocene-Eocene Thermal Maximum at the Alamedilla section (Spain). Geological Society of America Bulletin 2010; 122: 1616–1624. 10.1130/B30055.1

[41] Giusberti L, Coccioni R, Sprovieri M, Tateo F. Perturbation at the sea floor during the Paleocene–Eocene Thermal Maximum: evidence from benthic foraminifera at Contessa Road, Italy. Marine Micropaleontology 2009; 70: 102–119.

[42] Giusberti L, Boscolo Galazzo F, Thomas E. Variability in climate and productivity during the Paleocene–Eocene Thermal Maximum in the western Tethys (Forada section). Climate of the Past 2016; 12: 213–240. 10.5194/cp-12-213-2016

[43] Arreguín-Rodríguez GJ, Alegret L, Thomas E. Late Paleocene-middle Eocene benthic foraminifera on a Pacific seamount (Allison Guyot, ODP Site 865): Greenhouse climate and superimposed hyperthermal events. Paleoceanography 2016; 31: 346–364. 10.1002/2015PA002837

[44] Kennett JP, Stott LD. Abrupt deep-sea warming, palaeoceanographic changes and benthic extinctions at the end of the Palaeocene. Nature 1991; 353: 225–229.

[45] McInerney FA, Wing S. The Paleocene–Eocene Thermal Maximum: a perturbation of carbon cycle, climate, and biosphere with implications for the future. Annual Review of Earth and Planetary Sciences 2011; 39: 489–516.

[46] Dunkley-Jones T, Lunt DJ, Schmidt DN, Ridgwell A, Sluijs A, Valdez PJ, et al Climate model and proxy data constraints on ocean warming across the Paleocene-Eocene Thermal Maximum. Earth-Science Reviews 2013; 125: 123–145.

[47] Thomas E, Zachos JC. Was the late Paleocene thermal maximum a unique event? GFF 2000; 122: 169–170.

[48] Cramer BS, Wright JD, Kent DV, Aubry MP. Orbital climate forcing of δ^13^C excursions in the late Paleocene–early Eocene (chrons C24n–C25n). Paleoceanography 2003; 18(4): 1097 10.1029/2003PA000909.

[49] Zachos JC, Dickens GR, Zeebe RE. An early Cenozoic perspective on greenhouse warming and carbon-cycle dynamics. Nature 2008; 451: 279–283. 10.1038/nature06588 18202643

[50] Zachos JC, McCarren H, Murphy B, Röhl U, Westerhold T. Tempo and scale of late Paleocene and early Eocene carbon isotope cycles: implications for the origin of hyperthermals. Earth and Planetary Science Letters 2010; 299: 242–249.

[51] Littler K, Röhl U, Westerhold T, Zachos JC. A high-resolution benthic stable-isotope record for the South Atlantic: implications for orbital-scale changes in Late Paleocene–Early Eocene climate and carbon cycling. Earth and Planetary Science Letters 2014; 401: 18–30.

[52] Lauretano V, Littler K, Polling M, Zachos JC, Lourens LJ. Frequency, magnitude and character of hyperthermal events at the onset of the Early Eocene Climatic Optimum. Climate of the Past 2015; 11: 1313–1324. 10.5194/cp-11-1313-2015

[53] Lourens L, Sluijs A, Kroon D, Zachos JC, Thomas E, Röhl U, et al Astronomical modulation of late Palaeocene to early Eocene global warming events. Nature 2005; 435: 1083–1087. 10.1038/nature03814 15944716

[54] Nicolo MJ, Dickens GR, Hollis CJ, Zachos JC. Multiple early Eocene hyperthermals: their sedimentary expression on the New Zealand continental margin and in the deep sea. Geology 2007; 35: 699–702. 10.1130/G23648A.1.

[55] Stap L, Lourens L, Sluijs A, Thomas E. Patterns and magnitude of deep sea carbonate dissolution during Eocene Thermal Maximum 2 and H2, Walvis Ridge, southeastern Atlantic Ocean. Paleoceanography 2009; 24: PA1211 10.1029/2008PA001655

[56] Stap L, Lourens LJ, Thomas E, Sluijs A, Bohaty S, Zachos JC. High-resolution deep-sea carbon and oxygen isotope records of Eocene Thermal Maximum 2 and H2 and implications for the origin of early Paleogene hyperthermal events. Geology 2010; 38: 607–610. 10.1130/G30777.1

[57] Stap L, Lourens L, van Dijk A, Schouten S, Thomas E. Coherent pattern and timing of the carbon isotope excursion and warming during Eocene Thermal Maximum 2 as recorded in planktic and benthic foraminifera. Geochemistry, Geophyics, Geosystems 2010; 11: Q11011 10.1029/2010GC003097

[58] Leon-Rodriguez L, Dickens GR. Constraints on ocean acidification associated with rapid and massive carbon injections: the early Paleogene record at ocean drilling program site 1215, equatorial Pacific Ocean. Palaeogeography, Palaeoclimatology, Palaeoecology 2010; 298: 409–420.

[59] Payros A, Ortiz S, Alegret L, Orue-Etxebarria X, Apellaniz E, Molina E. An early Lutetian carbon-cycle perturbation: insights from the Gorrondatxe section (western Pyrenees, Bay of Biscay). Paleoceanography 2012; 27(2): PA2213 10.1029/2012PA002300.

[60] D’haenens S, Bornemann A, Stassen P, Speijer RP. Multiple early Eocene benthic foraminiferal assemblage and δ^13^C fluctuations at DSDP Site 401 (Bay of Biscay—NE Atlantic). Marine Micropaleontology 2012; 88–89: 15–35. 10.1016/j.marmicro.2012.02.006.

[61] Stassen P, Steurbaut E, Morsi AMM, Schulte P, Speijer RP. Biotic impact of Eocene Thermal Maximum 2 in a shelf setting (Dababiya, Egypt). Austrian Journal of Earth Sciences 2012; 105/1: 154–160.

[62] Jennions SM, Thomas E, Schmidt DN, Lunt D, Ridgwell A. Changes in benthic ecosystems and ocean circulation in the Southeast Atlantic across Eocene Thermal Maximum 2. Paleoceanography 2015; 30: 1059–1077. 10.1002/2015PA002821

[63] Arreguín-Rodríguez GJ, Alegret L. Deep-sea benthic foraminiferal turnover across early Eocene hyperthermal events at Northeast Atlantic DSDP Site 550. Palaeogeography, Palaeoclimatology, Palaeoecology 2016; 451: 62–72. 10.1016/j.palaeo.2016.03.010.

[64] Martini E. Standard Tertiary and Quarternary calcareous nannoplankton zonation. In: Farinacci A, editor. Proceedings of the 2nd Planktonic Conference. Rome, Italy: Tecnoscienza; 1971. pp. 739–785.

[65] Bukry D. Low-latitude biostratigraphic zonation. In: Edgar NT, Saunders JB, et al., editors. Initial Reports of the Deep Sea Drilling Project 15. Washington: U.S. Government Printing Office; 1973. pp. 685–703.

[66] Bukry D. Coccolith and silicoflagellate stratigraphy, northwestern Pacific Ocean, Deep Sea Drilling Project Leg 32. In: Larson RL, Moberly R, et al., editors. Initial Reports of the Deep Sea Drilling Project 32. Washington: U.S. Government Printing Office; 1975. pp. 677–701.

[67] Berggren WA, Kent DV, Swisher CC III, Aubry MP. A revised Cenozoic geochronology and chronostratigraphy In: Berggren WA, Kent DV, Swisher CC III, Aubry MP, Hardenbol J, editors. Geochronology, time scales and global stratigraphic correlation. USA: SEPM (Society for Sedimentary Geology) Special Publication 54; 1995 pp. 129–212.

[68] Berggren WA, Pearson PN. A revised tropical to subtropical Paleogene planktonic foraminiferal zonation. Journal of Foraminiferal Research 2005; 35(4): 279–298.

[69] Hay WW, DeConto RM, Wold CN, Wilson KM, Voigt S, Schulz M, et al Alternative global Cretaceous paleogeography In: Barrera E, Jonhson CC, editors. Evolution of the Cretaceous Ocean–Climate System. Boulder, Colorado: Geological Society of America Special Paper 332; 1999 pp. 1–47.

[70] Sager WW, Winterer EL, Firth JV, Arnaud HM, Baker PE, Baudin F, et al. Site 865. In: Sager WW, Winterer EL, Firth JV, et al., editors. Proceedings of the Ocean Drilling Program, Initial Reports 143. Texas, USA: College Station, ODP; 1993. pp. 111–180.

[71] Bralower TJ, Zachos JC, Thomas E, Parrow M, Paull CK, Kelly DC, et al Late Paleocene to Eocene paleoceanography of the equatorial Pacific Ocean: Stable isotopes recorded at Ocean Drilling Program Site 865, Allison Guyot. Paleoceanography 1995; 10: 841–865. 10.1029/95PA01143

[72] Bralower TJ, Premoli Silva I, Malone MJ, Arthur MA, Averyt K, Bown PR, et al. Site 1209. In: Bralower TJ, Premoli Silva I, Malone MJ, et al., editors. Proceedings of the Ocean Drilling Program, Initial Reports 198. Texas, USA: College Station, ODP; 2002. pp. 1–102.

[73] Bralower TJ, Premoli Silva I, Malone MJ, Arthur MA, Averyt K, Bown PR, et al. Leg 198 Summary. In: Bralower TJ, Premoli Silva I, Malone MJ, et al., editors. Proceedings of the Ocean Drilling Program, Initial Reports 198. Texas, USA: College Station, ODP; 2002. pp. 1–148.

[74] Bralower TJ, Premoli Silva I, Malone MJ, Arthur MA, Averyt K, Bown PR, et al. Site 1210. In: Bralower TJ, Premoli Silva I, Malone MJ, et al., editors. Proceedings of the Ocean Drilling Program, Initial Reports 198. Texas, USA: College Station, ODP; 2002. pp. 1–89.

[75] Bralower TJ, Premoli Silva I, Malone MJ, Arthur MA, Averyt K, Bown PR, et al. Site 1211. In: Bralower TJ, Premoli Silva I, Malone MJ, et al., editors. Proceedings of the Ocean Drilling Program, Initial Reports 198. Texas, USA: College Station, ODP; 2002. pp. 1–81.

[76] Bralower TJ, Premoli Silva I, Malone MJ, Arthur MA, Averyt K, Bown PR, et al. Site 1212. In: Bralower TJ, Premoli Silva I, Malone MJ, et al., editors. Proceedings of the Ocean Drilling Program, Initial Reports 198. Texas, USA: College Station, ODP; 2002. pp. 1–79.

[77] Lyle MW, Wilson PA, Janecek TR, Backman J, Busch WH, Coxall HK, et al. Site 1215. In: Lyle MW, Wilson PA, Janecek TR, et al., editors. Proceedings of the Ocean Drilling Program, Initial Reports 199. Texas, USA: College Station, ODP; 2002. pp. 1–60.

[78] Lyle MW, Wilson PA, Janecek TR, Backman J, Busch WH, Coxall HK, et al. Site 1220. In: Lyle MW, Wilson PA, Janecek TR, et al., editors. Proceedings of the Ocean Drilling Program, Initial Reports 199. Texas, USA: College Station, ODP; 2002. pp. 1–93.

[79] Lyle MW, Wilson PA, Janecek TR, Backman J, Busch WH, Coxall HK, et al. Site 1221. In: Lyle MW, Wilson PA, Janecek TR, et al., editors. Proceedings of the Ocean Drilling Program, Initial Reports 199. Texas, USA: College Station, ODP; 2002. pp. 1–66.

[80] Montadert L, Roberts DG, Auffret GA, Bock WD, Dupeuble PA, Hailwood EA, et al. Site 401. In: Montadert L, Roberts DG, et al., editors. Initial reports of the Deep Sea Drilling Project 48. Washington: U.S. Government Printing Office; 1979. pp. 71–123.

[81] De Graciansky PC, Poag CW, Cunningham RJr, Loubere P, Masson DG, Mazzullo JM, et al. Site 550. In: De Graciansky PC, Poag CW, et al., editors. Initial reports of the Deep Sea Drilling Project 80. Washington: U.S. Government Printing Office; 1985. pp. 251–355.

[82] D’haenens S, Bornemann A, Claeys P, Röhl U, Steurbaut E, Speijer RP. A transient deep-sea circulation switch during Eocene Thermal Maximum 2. Paleoceanography 2014; 29: 370–388. 10.1002/2013PA002567

[83] Norris RD, Kroon D, Klaus A, Alexander IT, Bardot LP, Barker CE, et al. Site 1051. In: Norris RD, Kroon D, Klaus A, et al., editors. Proceedings of the Ocean Drilling Program, Initial Reports 171B. Texas, USA: College Station, ODP; 1998. pp. 171–239.

[84] Erbacher J, Mosher DC, Malone MJ, Berti D, Bice KL, Bostock H, et al. Site 1258. In: Erbacher J, Mosher DC, Malone MJ, et al., editors. Proceedings of the Ocean Drilling Program, Initial Reports 207. Texas, USA: College Station, ODP; 2004. pp. 1–117.

[85] Westerhold T, Röhl U. High resolution cyclostratigraphy of the early Eocene—new insights into the origin of the Cenozoic cooling trend. Climate of the Past 2009; 5: 309–327.

[86] Zachos JC, Kroon D, Blum P, Bowles J, Gaillot P, Hasegawa T, et al. Site 1262. In: Zachos JC, Kroon D, Blum P, et al., editors. Proceedings of the Ocean Drilling Program, Initial Reports 208. Texas, USA: College Station, ODP; 2004. pp. 1–92.

[87] Röhl U, Westerhold T, Monechi S, Thomas E, Zachos JC, Donner B. The third and final early Eocene thermal maximum: Characteristics, timing, and mechanisms of the “X” event. Geological Society of America Abstract Programs 2005; 37(7): 264.

[88] Foster LC, Schmidt DN, Thomas E, Arndt S, Ridgwell A. Surviving rapid climate change in the deep-sea during the Paleogene hyperthermals. Proceedings of the National Academies of Science 2013; 110: 9273–9276. 10.1073/pnas.1300579110 23690593 PMC3677492

[89] Zachos JC, Kroon D, Blum P, Bowles J, Gaillot P, Hasegawa T, et al. Site 1263. In: Zachos JC, Kroon D, Blum P, et al., editors. Proceedings of the Ocean Drilling Program, Initial Reports 208. Texas, USA: College Station, ODP; 2004. pp. 1–87.

[90] Barker PF, Kennett JP, O’Connell S, Berkowitz S, Bryant WR, Burckle LH, et al. Site 689. In: Barker PF, Kennett JP, et al., editors. Proceedings of the Ocean Drilling Program, Initial Reports 113. Texas, USA: College Station, ODP; 1988. pp. 89–181.

[91] Barker PF, Kennett JP, O’Connell S, Berkowitz S, Bryant WR, Burckle LH, et al. Site 690. In: Barker PF, Kennett JP, et al., editors. Proceedings of the Ocean Drilling Program, Initial Reports 113. Texas, USA: College Station, ODP; 1988. pp. 183–292.

[92] Peirce J, Weissel J, Taylor E, Dehn J, Driscoll N, Farrell J, et al. Site 752. In: Peirce J, Weissel J, et al., editors. Proceedings of the Ocean Drilling Program, Initial Reports 121. Texas, USA: College Station, ODP; 1989. pp. 111–169.

[93] Nomura R. Paleoceanography of upper Maestrichtian to Eocene benthic foraminiferal assemblages at sites 752, 753 and 754, eastern Indian Ocean. In: Weissel J, Peirce J, et al., editors. Proceedings of the Ocean Drilling Program, Scientific Results 121. Texas, USA: College Station, ODP; 1991. pp. 3–29.

[94] Patterson RT, Fishbein E. Re-examination of the statistical methods used to determine the number of point counts needed for micropaleontological quantitative research. Journal of Paleontology 1989; 63: 245–248.

[95] Winguth A, Thomas E, Winguth C. Global decline in ocean ventilation, oxygenation and productivity during the Paleocene-Eocene Thermal Maximum—Implications for the benthic extinction. Geology 2012; 40: 263–266.

[96] Bignot G. Middle Eocene benthic foraminifers from Holes 960A and 960C, Central Atlantic Ocean. In: Mascle J, Lohmann GP, Moullade M, editors. Proceedings of the Ocean Drilling Program, Scientific Results 159. Texas, USA: College Station, ODP; 1998. pp. 433–444.

[97] Speijer RP, Schmitz B. A benthic foraminiferal record of Paleocene sea level and trophic/redox conditions at Gebel Aweina, Egypt. Palaeogeography, Palaeoclimatology, Palaeoecology 1998; 137(1–2): 79–101.

[98] Deprez A, Tesseur S, Stassen P, D’haenens S, Steurbaut E, King C, et al Early Eocene environmental development in the northern Peri-Tethys (Aktulagay, Kazakhstan) based on benthic foraminiferal assemblages and stable isotopes (O, C). Marine Micropaleontology 2015; 115: 59–71.

[99] Speijer RP. Extinction and recovery patterns in benthic foraminiferal paleocommunities across the Cretaceous-Paleogene and Paleocene-Eocene boundaries. Geologica Ultraiectina 124, Ph.D. Thesis, Universiteit Utrecht. 1994. https://dspace.library.uu.nl/handle/1874/274537.

[100] Jorissen FJ, Stigter HC, Widmark JGV. A conceptual model explaining benthic foraminiferal microhabitats. Marine Micropaleontology 1995; 26: 3–15.

[101] Jorissen FJ, Fontanier C, Thomas E. Paleoceanographical proxies based on deep-sea benthic foraminiferal assemblage characteristics In: Hillaire-Marcel C, de Vernal A, editors. Proxies in Late Cenozoic Paleoceanography: Pt. 2: Biological tracers and biomarkers. Elsevier; 2007 pp. 263–326.

[102] Corliss BH. Microhabitats of benthic foraminifera within deep-sea sediments. Nature 1985; 314: 435–438.

[103] Corliss BH. Morphology and microhabitat preferences of benthic foraminifera from the northwest Atlantic Ocean. Marine Micropaleontology 1991; 17: 195–236.

[104] Rathburn AE, Corliss BH. The ecology of living (stained) deep-sea benthic foraminifera from the Sulu Sea. Paleoceanography 1994; 9: 87–150.

[105] Jorissen FJ. Benthic foraminiferal successions across Late Quaternary Mediterranean sapropels. Marine Geology 1999; 153: 91–101.

[106] Buzas MA, Culver SJ, Jorissen FJ. A statistical evaluation of the microhabitats of living (stained) infaunal benthic foraminifera. Marine Micropaleontology 1993; 20: 311–320.

[107] Gooday AJ. Benthic foraminifera (Protista) as tools in deep-water paleoceanography: environmental influences on fauna characteristics In: Southward AJ, Tyler PA, Young CM, Fuiman LA, editors. Advances in marine biology 46. UK: Academic Press; 2003 pp. 1–90. 10.1016/s0065-2881(03)46002-1 14601411

[108] John EH, Pearson PN, Coxall HK, Birch H, Wade BS, Foster GL. Warm ocean processes and carbon cycling in the Eocene. Philosophical Transactions of the Royal Society A 2013; 371: 20130099 10.1098/rsta.2013.0099 24043871

[109] Ma Z, Gray E, Thomas E, Murphy B, Zachos JC, Paytan A. Carbon sequestration during the Paleocene-Eocene thermal maximum by an efficient biological pump. Nature Geoscience 2014; 7: 382–388. 10.1038/NGEO2139

[110] Hammer Ø, Harper DAT, Ryan PD. PAST: Paleontological Statistics Software Package for Education and Data Analysis. Palaeontologia Electronica 2001; 4(1): 1–9.

[111] Hammer Ø, Harper DAT. Paleontological Data Analysis. Blackwell Publishing; 2006.

[112] Wright J. A list of the Cretaceous foraminifera of Keady Hill, County Derry. Belfast Naturalists Field Club Proceedings 1886; 1: 330.

[113] Howe HV. Louisiana Cook Mountain Eocene foraminifera. Louisiana Geol. Surv. Bull. 1939; 14: 1–122.

[114] Cushman JA. The foraminifera of the Choctawhatchee Formation of Florida. Florida State Geol. Survey Bulletin 1930; 4: 1–89.

[115] Loeblich AR Jr, Tappan H. Suprageneric classification of the Rhizopodea. Jour. Paleontol. 1961; 35(2): 245–330.

[116] Loeblich AR Jr, Tappan H. Foraminifera genera and their classification. New York, USA: Van Nostrand Reinhold Company Inc.; 1987.

[117] Alegret L, Ortiz S. Global extinction event in benthic foraminifera across the Paleocene/Eocene boundary at the Dababiya Stratotype section. Micropaleontology 2006; 52: 433–447.

[118] Ernst SR, Guasti E, Dupuis C, Speijer RP. Environmental perturbation in the southern Tethys across the Paleocene/Eocene boundary (Dababiya, Egypt): Foraminiferal and clay mineral records. Marine Micropaleontology 2006; 60: 89–111.

[119] Nørvang A. Interior characteristics of Bulimina (Foraminifera). Proceedings of the 23rd International Geological Congress 1968; 415–422.

[120] Jones RW. The Challenger Foraminifera. London, UK: Oxford Science Publications, The Natural History Museum; 1994.

[121] Terquem O. Les foraminifères de l’Eocène des environs de Paris. Paris, France: Société Géologique de France, Mémoires; 1882.

[122] Martin LT. Eocene foraminifera from the type Lodo formation, Fresno County, California. California, USA: Stanford University Press; 1943.

[123] Cole WS. A foraminiferal fauna from the Chapapote formation in Mexico. Bulletins of American Paleontology 1928; 14(53): 199–231.

[124] Cushman JA, Parker FL. Some American Cretaceous Buliminas. Contributions from the Cushman Laboratory for Foraminiferal Research 1935; 11: 96–101.

[125] Georgescu MD, Arz JA, Macauley RV, Kukulski RB, Arenillas I, Perez-Rodriguez I. Late Cretaceous (Santonian-Maastrichtian) serial foraminifera with pore mounds or pore mound-based ornamentation structures. Revista Española de Micropaleontologia 2011; 43(1–2): 109–139.

[126] Marsson T. Die Foraminiferen der weissen Schreibkreide der Insel Rügen. Mitteilungen des Naturwissenschaftlichen vereins für Neu-Vorpommern und Rügen in Greifswald 1878; 10: 115–196.

[127] Frenzel P. Die benthischen Foraminiferen der Rügener Schreibkreide (Unter-Maastrichtium, NE-Deutschland), Neue Paläontologische Abhandlungen. Dresden, Germany: C Press; 2000.

[128] Cushman JA. Some new genera of the foraminifera. Contributions from the Cushman Laboratory for Foraminiferal Research 1927; 2: 77–81.

[129] Speijer RP, Van Loo D, Masschaele B, Vlassenbroeck J, Cnudde V, Jacobs P. Quantifying foraminiferal growth with high-resolution X-ray computed tomography: New opportunities in foraminiferal ontogeny, phylogeny, and paleoceanographic applications. Geosphere 2008; 4(4): 760–763. 10.1130/GES00176.1

[130] Cushman JA, Parker FL. Some American Eocene Buliminas. Contributions from the Cushman Laboratory for Foraminiferal Research 1936; 12: 39–45.

[131] Brotzen F. The Swedish Paleocene and its foraminiferal fauna. Stockholm, Sweden: Norstedt, Sveriges Geologiska Undersökning; 1948.

[132] Olshanetskiy DM. Deep-water benthic foraminifers from the Paleocene and Eocene of the North Pacific Region: Paleontology, biostratigraphy, and paleoceanological reconstructions. Stratigraphy and Geological Correlation 2015; 23(7): 661–715.

[133] LeRoy LW. Biostratigraphy of the Maqfi section, Egypt. New York, USA: Geological Society of America Memoirs; 1953.

[134] Hofker J. The foraminifera of the Siboga Expedition; Part III. Leiden: Siboga Exped., Monogr.; 1951.

[135] Alegret L, Thomas E. Cretaceous/Paleogene boundary bathyal paleoenvironments in the central North Pacific (DSDP [Deep Sea Drilling Project] Site 465), the Northwestern Atlantic (ODP [Ocean Drilling Program] Site 1049), the Gulf of Mexico and the Tethys: The benthic foraminiferal record. Palaeogeography, Palaeoclimatology, Palaeoecology 2005; 224: 53–82.

[136] Alegret L, Thomas E. Benthic foraminifera across the Cretaceous/Paleogene boundary in the Southern Ocean (ODP Site 690): Diversity, food and carbonate saturation. Marine Micropaleontology 2013; 105: 40–51.

[137] Sprong J, Kouwenhoven TJ, Bornemann A, Dupuis C, Speijer RP, Stassen P, et al In search of the latest Danian event in a paleobathymetric transect off Kasserine Island, north-Central Tunisia. Palaeogeography, Palaeoclimatology, Palaeoecology 2013; 379–380: 1–16. 10.1016/j.palaeo.2013.01.018.

[138] Cushman JA, Renz HH. Eocene, Midway, foraminifera from Soldado Rock, Trinidad. Contributions from the Cushman Laboratory for Foraminiferal Research 1942; 18: 1–14.

[139] Thomas E. Extinction and food at the seafloor: A high-resolution benthic foraminiferal record across the Initial Eocene Thermal Maximum, Southern Ocean Site 690 In: Wing SL, Gingerich PD, Schmitz B, Thomas E, editors. Causes and consequences of globally warm climates in the Early Paleogene. Boulder, Colorado: Geological Society of America Special Paper 369; 2003 pp. 319–332.

[140] Morrow AL. Foraminifera and ostracoda from the Upper Cretaceous of Kansas. Journal of Paleontology 1934; 8: 186–205.

[141] Pardo A, Ortiz N, Keller G. Latest Maastrichtian and Cretaceous-Tertiary boundary foraminiferal turnover and environmental changes at Agost, Spain In: McLeod N, Keller G, editors. Cretaceous-Tertiary mass extinctions: Biotic and environmental changes. New York: WW Norton & Company; 1996 pp. 139–171.

[142] Alegret L, Molina E, Thomas E. Benthic foraminiferal turnover across the Cretaceous/Paleogene boundary at Agost (southeastern Spain): paleoenvironmental inferences. Marine Micropaleontology 2003; 48: 251–279.

[143] Brotzen F. Foraminiferen aus dem schwedischen untersten Senon von Eriksdal in Schonen. Stockholm, Sweden: Norstedt, Sveriges Geologiska Undersökning; 1936.

[144] Koutsoukos EAM, De Klasz I. Late Cretaceous foraminiferal biogeography (Families Bolivinidae, Buliminellidae, Gavelinellidae, Siphogenerinoididae, Turrilinidae) in northeastern Brazilian shelf and central West African basins. Cretaceous Research 2000; 21: 381–405.

[145] Brady HB. Notes on some of the reticularian Rhizopoda of the “Challenger” expedition, Part III. Quarterly Journal of Microscopical Science 1881; 21: 31–71.

[146] Cushman JA. Cretaceous foraminifera of the family Chilostomellidae. Contributions from the Cushman Laboratory for Foraminiferal Research 1936; 12: 71–102.

[147] d’Orbigny A. Voyage dans l’Amérique Méridionale: Foraminifères. Paris, France: P. Bertrand; 1839.

[148] Alegret L, Thomas E. Food supply to the seafloor in the Pacific Ocean after the Cretaceous/Paleogene boundary event. Marine Micropaleontology 2009; 73: 105–116.

[149] Schnitker D, Tjalsma RC. New genera and species of benthic foraminifers from Paleocene and Eocene deep-water deposits. Journal of Foraminiferal Research 1980; 10(3): 235–241.

[150] Earland A. Foraminifera; Part IV-Additional records from the Weddell Sea sector from material obtained by the S. Y. ‘Scotia’. Discovery Reports 1936; 13: 1–76.

[151] Saidova KhM. Benthonic foraminifera of the Pacific Ocean. Moscow, Russia: Akademiia Nauk SSSR, Institut Okeanologii im. P.P. Shirshova; 1975.

[152] Lipps JH. Revision of the foraminiferal family Pseudoparrellidae Voloshinova. Tulane Studies in Geology 1965; 3: 117–148.

[153] Cushman JA, Bermúdez PJ. Additional species of Paleocene foraminifera from the Madruga formation of Cuba. Contributions from the Cushman Laboratory for Foraminiferal Research 1948; 24: 85–89.

[154] Vasilenko VP. Paleocene foraminifera of the central part of the Dnieper-Donets Basin. In: Microfauna of the USSR Collection 4. Leningrad: Trudy VNIGRI; 1950. pp. 177–224.

[155] Ortiz S, Thomas E. Lower-middle Eocene benthic foraminifera from the Fortuna Section (Betic Cordillera, southeastern Spain). Micropaleontology 2006; 52(2): 97–150.

[156] Bermúdez PJ. Tertiary smaller foraminifera of the Dominican Republic. Cushman Laboratory for Foraminiferal Research Special Publication 1949; 25: 1–322.

[157] Alegret L, Thomas E. Deep-sea environments across the Cretaceous/Paleogene boundary in the eastern South Atlantic Ocean (ODP Leg 208, Walvis Ridge). Marine Micropaleontology 2007; 64: 1–17.

[158] Plummer HJ. Foraminifera of the Midway formation in Texas. Austin, Texas: Texas University Press; 1927.

[159] Nuttall WLF. Tertiary foraminifera from the Naparima region of Trinidad (British West Indies). Quarterly Journal of the Geological Society 1928; 84: 57–116.

[160] Berggren WA, Aubert J. Paleocene benthonic foraminiferal biostratigraphy, paleobiogeography and paleoecology of Atlantic-Tethyan regions: Midway-type fauna. Palaeogeography, Palaeoclimatology, Palaeoecology 1975; 18: 73–192.

[161] Cushman JA, Renz HH. New Oligocene-Miocene foraminifera from Venezuela. Contributions from the Cushman Laboratory for Foraminiferal Research 1941; 17: 1–27.

[162] Cushman JA, Stainforth RM. The foraminifera of the Cipero marl formation of Trinidad, British West Indies. Cushman Laboratory for Foraminiferal Research Special Publication 1945; 14: 1–75.

[163] Cushman JA. Some new Recent foraminifera from the Tropical Pacific. Contributions from the Cushman Laboratory for Foraminiferal Research 1933; 9: 77–95.

[164] Brotzen F. Flintrännans och Trindelrännans geologi (Öresund). Stockholm, Sweden: Norstedt, Sveriges Geologiska Undersökning; 1940.

[165] Belford DJ. The genera Nuttallides Finlay, 1939, and *Nuttallina*, n. gen. Contributions from the Cushman Foundation for Foraminiferal Research 1958; 9: 93–98.

[166] Belford DJ. *Nuttallinella*, new name for *Nuttallina* Belford, 1958 (non *Nuttallina* Dall, 1871). Contributions from the Cushman Foundation for Foraminiferal Research 1959; 10: 20.

[167] Galloway JJ, Morrey M. A lower Tertiary foraminiferal fauna from Manta, Ecuador. Bulletins of American Paleontology 1929; 15(55): 1–56.

[168] Erwin DH. The end and the beginning: recovery from mass extinctions. Trends in Ecology and Evolution 1998; 13: 344–349. 21238338 10.1016/s0169-5347(98)01436-0

[169] Levin LA. Oxygen minimum zone benthos: Adaptation and community response to hypoxia. Oceanography and Marine Biology: An Annual Review 2003; 41: 1–45.

[170] Fontanier C, Jorissen FJ, Chaillou G, Anschutz P, Grémare A, Griveaud C. Live foraminiferal faunas from a 2800 m deep lower canyon station from the Bay of Biscay: Faunal response to focusing of refractory organic matter. Deep-Sea Research I 2005; 52: 1189–1227.

[171] Kender S, McClymont EL, Elmore AC, Emanuele D, Leng MJ, Elderfield H. Mid Pleistocene foraminiferal mass extinction coupled with phytoplankton evolution. Nature Communications 2016; 7: 11970 10.1038/ncomms11970 27311937 PMC4915025

[172] Heinz P, Ruepp D, Hemleben C. Benthic foraminifera assemblages at Great Meteor Seamount. Marine Biology 2004; 144: 985–998.

[173] Genin A. Bio-physical coupling in the formation of zooplankton and fish aggregations over abrupt topographies. Journal of Marine Systems 2004; 50: 3–20.

[174] Thomas E, Booth L, Maslin M, Shackleton NJ. Northeastern Atlantic benthic foraminifera during the last 45,000 years: changes in productivity seen from the bottom up. Paleoceanography 1995; 10: 545–562.

[175] Diz P, Barker S. Approaches and constraints to the reconstruction of palaeoproductivity from Cape Basin abyssal benthic foraminifera (South Atlantic). Journal of Micropalaeontology 2016; 35: 195–204. 10.1144/jmpaleo2015-045

[176] Stassen P, Thomas E, Speijer RP. Paleocene–Eocene Thermal Maximum environmental change in the New Jersey Coastal Plain: benthic foraminiferal biotic events. Marine Micropaleontology 2015; 115: 1–23.

[177] Deprez A, Jehle S, Bornemann A, Speijer RP. Pronounced biotic and environmental change across the latest Danian warming event (LDE) at Shatsky Rise, Pacific Ocean (ODP Site 1210). Marine Micropaleontology 2017; 137: 31–45. 10.1016/j.marmicro.2017.10.001

[178] Steineck PL, Thomas E. The latest Paleocene crisis in the deep sea: Ostracode succession at Maud Rise, Southern Ocean. Geology 1996; 24(7): 583–586.

[179] Westerhold T, Röhl U, Frederichs T, Agnini C, Raffi I, Zachos JC, et al Astronomical calibration of the Ypresian time scale: Implications for seafloor spreading rates and the chaotic behaviour of the solar system? Climate of the Past 2017; 13: 1129–1152. 10.5194/cp-13-1129-2017.

[180] Olsson RK, Miller KG, Browning JV, Wright JD, Cramer BS. Sequence stratigraphy and sea-level change across the Cretaceous-Tertiary boundary on the New Jersey passive margin In: Koeberl C, MacLeod KG, editors. Catastrophic Events and Mass Extinctions: Impacts and Beyond. Boulder, Colorado: Geological Society of America Special Paper 356; 2002 pp. 97–108.

[181] Self-Trail J, Robinson MM, Bralower TJ, Sessa JA, Hajek EA, Kump LR, et al Shallow marine response to global climate change during the Paleocene-Eocene Thermal Maximum, Salisbury Embayment, USA. Paleoceanography 2017; 32: 710–728. 10.1002/2017PA003096

[182] Widmark JGV, Speijer RP. Benthic foraminiferal ecomarker species of the terminal Cretaceous (late Maastrichtian) deep-sea Tethys. Marine Micropaleontology 1997; 31: 135–155.

[183] Nguyen TMP, Petrizzo MR, Speijer RP. Experimental dissolution of a fossil foraminiferal assemblage (Paleocene-Eocene Thermal Maximum, Dababiya, Egypt): Implications for a paleoenvironmental reconstructions. Marine Micropaleontology 2009; 73: 241–258. 10.1016/j.marmicro.2009.10.005

[184] Boscolo Galazzo F, Giusberti L, Luciani V, Thomas E. Paleoenvironmental changes during the Middle Eocene Climatic Optimum (MECO) and its aftermath: the benthic foraminiferal record from the Alano section (NE Italy). Palaeogeography, Palaeoclimatology, Palaeoecology 2013; 378: 22–35.

[185] Boscolo Galazzo F, Thomas E, Giusberti L. Benthic foraminiferal response to the Middle Eocene Climatic Optimum (MECO) in the South-Eastern Atlantic (ODP Site 1263). Palaeogeography, Palaeoclimatology, Palaeoecology 2015; 417: 432–444.

[186] Martin JH, Knauer GA, Karl DM, Broenkow WW. VERTEX: Carbon cycling in the North-East Pacific. Deep-Sea Research 1987; 34(2): 267–285.

[187] Herguera JC, Berger WH. Paleoproductivity from benthic foraminifera abundance: Glacial to postglacial change in the west-equatorial Pacific. Geology 1991; 19: 1173–1176.

[188] Henson S, Sanders R, Masden E. Global patterns in efficiency of particulate organic carbon export and transfer to the deep ocean. Global Biogeochemical Cycles 2012; 26: GB1028 10.1029/2011GB004099

[189] Arndt S, Jørgensen BB, LaRowe DE, Middelburg JJ, Pancost RD, Regnier P. Quantifying the degradation of organic matter in marine sediments: A review and synthesis. Earth-Science Reviews 2013; 123: 53–86. 10.1016/j.earscirev.2013.02.008

[190] Kaiho K. Benthic foraminiferal dissolved-oxygen index and dissolved oxygen levels in the modern ocean. Geology 1994; 8: 719–722.

[191] Norris RD, Turner SK, Hull PM, Ridgwell A. Marine ecosystem responses to Cenozoic global change. Science 2013; 341(6145): 492–498. 10.1126/science.1240543 23908226

[192] Nardelli MP, Barras C, Metzger E, Mouret A, Filipsson HL, Jorissen F, et al Experimental evidence for foraminiferal calcification under anoxia. Biogeosciences 2014; 11: 4029–4038. 10.5194/bg-11-4029-2014

[193] Dickson AJ, Cohen AS, Coe AL. Seawater oxygenation during the Paleocene-Eocene Thermal Maximum. Geology 2012; 40(7): 639–642.

[194] Dickson AJ, Cohen AS. A molybdenum isotope record of Eocene Thermal Maximum 2: Implications for global ocean redox during the early Eocene. Paleoceanography 2012; 27: PA3230 10.1029/2012PA002346

[195] Zhou X, Thomas E, Winguth AME, Ridgwell A, Scher H, Hoogakker BAA, et al Expanded oxygen minimum zones during the late Paleocene—early Eocene: hints from multi-proxy comparison and ocean modeling. Paleoceanography 2016; 31(12): 1532–1546. 10.1002/2016PA003020

[196] Chun COJ, Delaney ML, Zachos JC. Paleoredox changes across the Paleocene-Eocene thermal maximum, Walvis Ridge (ODP Sites 1262, 1263, and 1266): Evidence from Mn and U enrichment factors. Paleoceanography 2010; 25: PA4202 10.1029/2009PA001861

[197] Pälike C, Delaney ML, Zachos J. Deep-sea redox across the Paleocene-Eocene thermal maximum. Geochemistry, Geophysics, Geosystems 2014; 15: 1038–1053. 10.1002/2013GC005074

[198] Zhou X, Thomas E, Rickaby REM, Winguth AME, Lu Z. I/Ca evidence for global upper ocean deoxygenation during the Paleocene-Eocene thermal maximum (PETM). Paleoceanography 2014; 29: 964–975. 10.1002/2014PA002702

[199] Post JE, Thomas E, Heaney PJ. Jianshuiite in oceanic manganese nodules at the Paleocene-Eocene boundary. American Mineralogist 2016; 101: 407–414.

[200] Risgaard-Petersen N, Langezaal AM, Ingvardsen S, Schmid MC, Jetten MSM, Op den Camp HJM, et al Evidence for complete denitrification in a benthic foraminifer. Nature 2006; 443: 93–96. 10.1038/nature05070 16957731

[201] Piña-Ochoa E, Hoegslund S, Geslin E, Cedhagen T, Revsbech NP, Nielsen LP, et al Widespread occurrence of nitrate storage and denitrification among Foraminifera and Gromiida. Proceedings of the National Academy of Sciences 2010; 107: 1148–1153. 10.1073/pnas.0908440107 PMC2824274 20080540

[202] Glock N, Schönfeld J, Eisenhauer A, Hensen C, Mallon J, Sommer S. The role of benthic foraminifera in the benthic nitrogen cycle of the Peruvian oxygen minimum zone. Biogeosciences 2013; 10: 4767–4783. 10.5194/bg-10-4767-2013

[203] Langlet D, Baal C, Geslin E, Metzger E, Zuschin M, Riedel B, et al Foraminiferal species responses to in situ, experimentally induced anoxia in the Adriatic Sea. Biogeosciences 2014; 11: 1775–1797. 10.5194/bg-11-1775-2014

[204] Zeebe RE. History of seawater carbonate chemistry, atmopsheric CO_2_, and ocean acidification. Annual Review of Earth and Planetary Sciences 2012; 40: 141–165.

[205] Alegret L, Thomas E, Lohmann K. End-Cretaceous marine mass extinction not caused by productivity collapse. Proceedings of the National Academy of Sciences 2012; 109(3): 728–732. 10.1073/pnas.1110601109 PMC3271934 22207626

[206] Wood KC, Miller KG, Lohmann GP. Middle Eocene to Oligocene benthic foraminifera from the Oceanic Formation, Barbados. Micropaleontology 1985; 31(2): 181–197.

