# Supplementary material for: Early Eocene deep-sea benthic foraminiferal faunas: Recovery from the Paleocene Eocene Thermal Maximum extinction in a greenhouse world
Source: PLoS One. 2018 Feb 23;13(2):e0193167. doi: 10.1371/journal.pone.0193167 (PMC5825042; doi:10.1371/journal.pone.0193167)
Supplement: S1 Appendix — Most common benthic foraminifera during the early Eocene and other species mentioned in the text. (DOCX) [file pone.0193167.s001.docx]

**S1 Appendix**

Taxonomic list of most common benthic foraminifera during the early Eocene and other species mentioned in the text.

*Abyssamina incisa* Schnitker and Tjalsma 1980

*Abyssamina poagi* Schnitker and Tjalsma 1980

*Abyssamina quadrata* Schnitker and Tjalsma 1980

*Alabamina dissonata* (Cushman and Renz) = *Pulvinulinella atlantisae* Cushman var. *dissonata* Cushman and Renz 1948

*Alabaminella* *weddellensis* (Earland) = *Eponides weddellensis* Earland 1936

*Anomalinoides acutus* (Plummer) = *Anomalina ammonoides* (Reuss) var. *acuta* Plummer 1927

*Anomalinoides ammonoides* (Reuss) = *Rotalina ammonoides* Reuss 1844

*Anomalinoides capitatus* (Gümbel) = *Rotalia capitata* Gümbel 1868

*Anomalinoides danicus* (Brotzen) = *Cibicides danica* Brotzen 1940

*Anomalinoides praeacutus* (Vasilenko) = *Anomalina praeacuta* Vasilenko 1950

*Anomalinoides praespissiformis* (Cushman and Bermudez) = *Anomalina praespissiformis* Cushman & Bermudez 1948

*Anomalinoides rubiginosus* (Cushman) = *Anomalina rubiginosa* Cushman, 1926

*Anomalinoides spissiformis* (Cushman and Stainforth) = *Anomalina alazanensis* Nuttall, var. *spissiformis* Cushman and Stainforth 1945

*Anomalinoides trinitatensis* (Nuttall) = *Truncatulina trinitatensis* Nutall 1928

*Aragonia aragonensis* (Nuttall) = *Textularia aragonensis* Nuttall 1930

*Bolivina huneri* (Howe) = *Bolivina huneri* Howe 1939

*Bolivinoides crenulata* (Cushman) = *Bolivina crenulata* Cushman 1936a

*Bolivinoides decoratus* (Jones) = *Bolivina decorata* Jones 1886

*Bulimina beaumonti* Cushman and Renz 1946

*Bulimina bradburyi* Martin, 1943

*Bulimina elongata* d'Orbigny 1846

*Bulimina jarvisi* Cushman and Parker 1936

*Bulimina kugleri* Cushman and Renz 1942

*Bulimina ovula* d'Orbigny 1839

*Bulimina prolixa* Cushman and Parker 1935

*Bulimina semicostata* Nuttall 1930

*Bulimina simplex* Terquem 1882

*Bulimina thanetensis* Cushman and Parker 1947

*Bulimina trihedra* Cushman 1926

*Bulimina trinitatensis* Cushman and Jarvis 1928

*Bulimina tuxpamensis* Cole 1928

*Bulimina virginiana* (Cushman) = *Angulogerina virginiana* Cushman 1944

*Buliminella beaumonti* Cushman and Renz 1946

*Buliminella grata* Parker and Bermudez 1937

*Cibicidoides alleni* (Plummer) = *Truncatulina alleni* Plummer 1926

*Cibicidoides dayi* (White) = *Planulina dayi* White 1928

*Cibicidoides eocaenus* (Gümbel) = *Rotalia* *eocaena* Gümbel 1868

*Cibicidoides howelli* (Toulmin) = *Cibicides howelli* Toulmin 1941

*Cibicidoides micrus* (Bermúdez) = *Cibicides micrus* Bermúdez 1949

*Cibicidoides mundulus* (Brady, Parker & Jones) = *Truncatulina mundula* Brady, Parker & Jones 1888

*Cibicidoides perlucidus* (Nuttall) = *Cibicides perlucida* Nuttall 1932

*Cibicidoides praemundulus* Berggren and Miller 1986

*Cibicidoides proprius* Brotzen 1948

*Cibicidoides pseudoperlucidus* (Bykova) = *Gemellides* *pseudoperlucidus* Bykova, in Vasilenko 1954

*Cibicidoides subcarinatus* (Cushman and Deaderick) = *Cibicides subcarinatus* Cushman & Deaderick 1944

*Cibicidoides subspiratus* (Nuttall) = *Cibicides subspirata* Nuttall 1930

*Cibicidoides tuxpamensis* (Cole) = *Cibicides tuxpamensis* Cole 1928

*Cibicidoides ungerianus* (d’ Orbigny) = *Rotalina ungeriana* d’Orbigny 1846

*Clinapertina complanata* Tjalsma and Lohmann 1983

*Clinapertina inflata* Tjalsma and Lohmann 1983

*Clinapertina subplanispira* Tjalsma and Lohmann 1983

*Eilohedra weddellensis* (Earland) = *Eponides weddellensis* Earland 1936

*Epistominella exigua* (Brady) = *Pulvinulina exigua* Brady 1884

*Eponides elevatus* (Plummer) = *Truncatulina elevata* Plummer 1927

*Fursenkoina fusiformis* (Cushman) = *Virgulina fusiformis* Cushman 1930

*Globocassidulina subglobosa* (Brady) = *Cassidulina subglobosa* Brady 1881

*Gyroidinoides depressus* (Alth) = *Rotalina depressa* Alth 1850

*Gyroidinoides planulatus* (Cushman and Renz) = *Gyroidina planulata* Cushman and Renz 1941

*Gyroidinoides complanata* (Cushman and Stainforth) = *Gyroidina complanata* Cushman & Stainforth 1945

*Hanzawaia ammophila* (Guembel) = *Rotalia ammophila* Guembel 1868

*Hanzawaia mantaensis* (Galloway and Morrey) = *Anomalina mantaensis* Galloway and Morrey, 1929

*Nonion havanense* Cushman and Bermudez 1937

*Nonionella robusta* Plummer 1931

*Nuttallides truempyi* (Nuttall) = *Eponides trümpyi* Nuttall 1930

*Nuttallides umbonifera* (Cushman) = *Pulvinulinella umbonifera* Cushman 1933

*Oridorsalis umbonatus* (Reuss) = *Rotalina umbonata* Reuss 1851

*Praebulimina reussi* Morrow 1964

*Pullenia americana* Cushman, 1936

*Pullenia jarvisi* Cushman 1936

*Pullenia subcarinata* (d'Orbigny) = *Nonionina subcarinata* Orbigny 1839

*Pyramidina rudita* (Cushman and Parker) = *Bulimina rudita* Cushman & Parker 1936

*Quadrimorphina profunda* Schnitker and Tjalsma 1980

*Seabrookia rugosa* Watanabe 1989

*Siphogenerinoides brevispinosa* Cushman 1939

*Stainforthia fusiformis* (Cushman) = *Virgulina fusiformis* Cushman 1930

*Tappanina selmensis* (Cushman) emended Brotzen 1948 = *Bolivinita selmensis* Cushman 1933

*Turrilina brevispira* Ten Dam 1944

*Valvalabamina depressa* (Alth) = *Rotalina depressa* Alth 1850
