# Supplementary figures and images for: Early Eocene deep-sea benthic foraminiferal faunas: Recovery from the Paleocene Eocene Thermal Maximum extinction in a greenhouse world
Source: PLoS One. 2018 Feb 23;13(2):e0193167. doi: 10.1371/journal.pone.0193167 (PMC5825042; doi:10.1371/journal.pone.0193167)

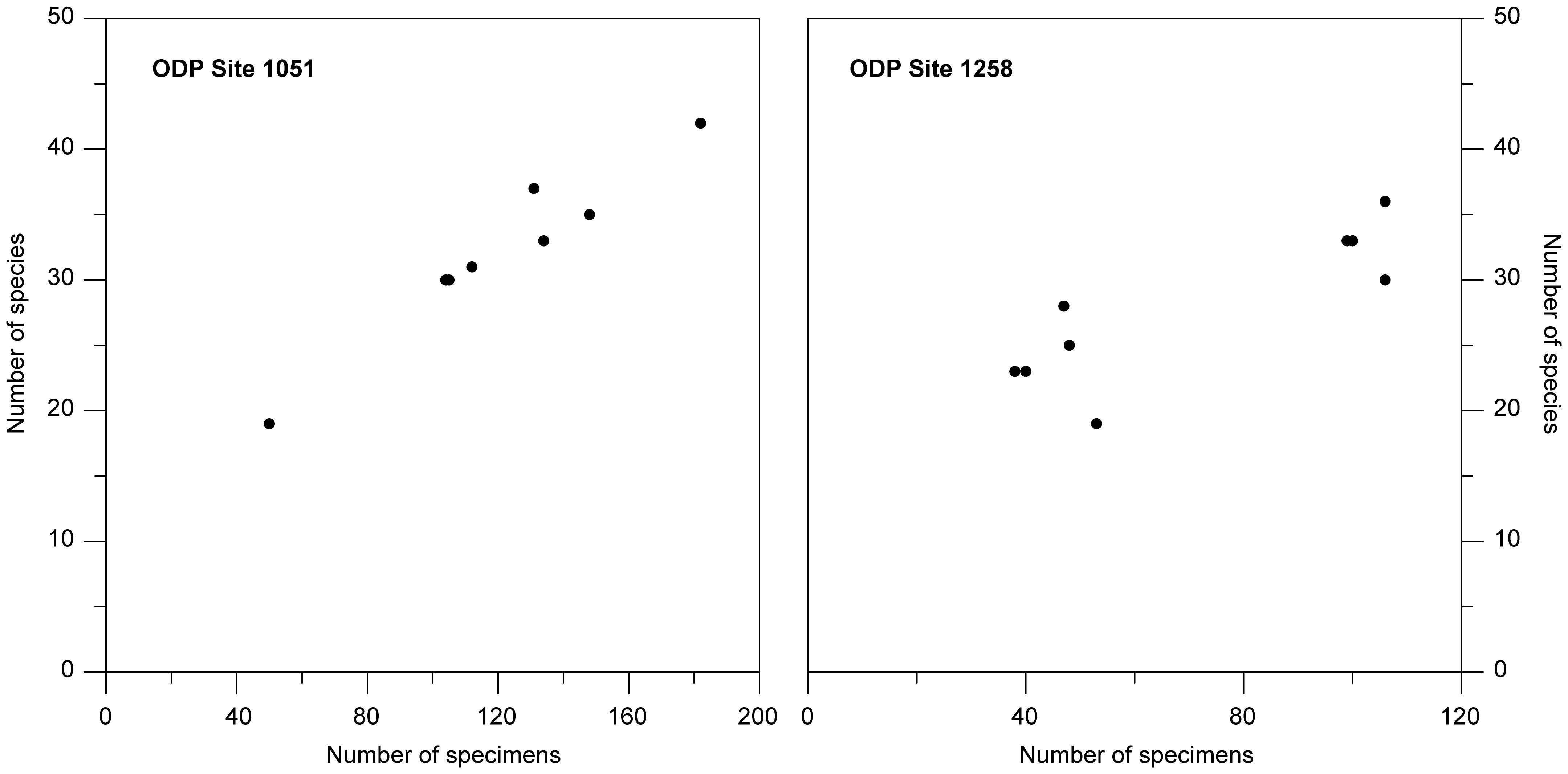

Supplement: S1 Fig — (TIF) [file pone.0193167.s002.tif]
